# Fine Mapping and Characterization of Candidate Genes that Control Resistance to *Cercospora sojina* K. Hara in Two Soybean Germplasm Accessions

**DOI:** 10.1371/journal.pone.0126753

**Published:** 2015-05-19

**Authors:** Anh-Tung Pham, Donna K. Harris, James Buck, Aaron Hoskins, Jonathan Serrano, Hussein Abdel-Haleem, Perry Cregan, Qijian Song, H. Roger Boerma, Zenglu Li

**Affiliations:** 1 Center for Applied Genetic Technologies & Department of Crop and Soil Sciences, University of Georgia, Athens, Georgia, United States of America; 2 Department of Plant Pathology, University of Georgia, Griffin, Georgia, United States of America; 3 Soybean Genomics and Improvement Laboratory, United States Department of Agriculture-Agricultural Research Service, Beltsville, Maryland, United States of America; University of Guelph, CANADA

## Abstract

Frogeye leaf spot (FLS), caused by the fungus *Cercospora sojina* K. Hara, may cause a significant yield loss to soybean growers in regions with a warm and humid climate. Two soybean accessions, PI 594891 and PI 594774, were identified to carry a high level of resistance similar to that conditioned by the *Rcs3* gene in 'Davis'. Previously, we reported that the resistance to FLS in these two plant introductions (PIs) was controlled by a novel gene (s) on chromosome 13 that is different from *Rcs3*. To fine-map the novel FLS resistance gene(s) in these two PIs, F_2: 3_ seeds from the crosses between PI 594891 and PI 594774, and the FLS susceptible genotype 'Blackhawk' were genotyped with SNP markers that were designed based on the SoySNP50k iSelect BeadChip data to identify recombinant events and locate candidate genes. Analysis of lines possessing key recombination events helped narrow down the FLS-resistance genomic region in PI 594891 from 3.3 Mb to a 72.6 kb region with five annotated genes. The resistance gene in PI 594774 was fine-mapped into a 540 kb region that encompasses the 72.6 kb region found in PI 594891. Sequencing five candidate genes in PI 594891 identified three genes that have several mutations in the promoter, intron, 5', and 3' UTR regions. qPCR analysis showed a difference in expression levels of these genes in both lines compared to Blackhawk in the presence of *C*. *sojina*. Based on phenotype, genotype and haplotype analysis results, these two soybean accessions might carry different resistance alleles of the same gene or two different gene(s). The identified SNPs were used to develop Kompetitive Allele Specific PCR (KASP) assays to detect the resistance alleles on chromosome 13 from the two PIs for marker-assisted selection.

## Introduction

Frogeye leaf spot (FLS), caused by the fungus *Cercospora sojina* K. Hara, is a soybean disease that can significantly impact soybean yield in warm, humid regions. This disease was first reported in Japan in 1915 and then in the USA in 1924 [1]. The development of FLS depends on the growing conditions of soybean and also weather patterns [2]. Though FLS normally causes negligible to mild damage to soybean plants, it can quickly develop into a devastating epidemic if favored by warm and humid weather [3]. Warm temperatures and high levels of rainfall and humidity induced two severe outbreaks of this disease in Argentina in the growing seasons of 1999/2000 and 2009/2010 [4, 5]. The epidemic of FLS in 1999/2000 affected mainly northwestern Argentina and caused yield losses from 25 to 48% in susceptible cultivars [4]. In the growing season of 2009/2010, favorable weather conditions for FLS and planting of susceptible cultivars resulted in 100% incidence of FLS in the Pampean region [5]. The yield loss caused by FLS in the latter incidence was estimated to be from 4 to 5 million metric tons, making FLS the most expensive disease in the history of soybean production in Argentina. In the USA, FLS also is a threat to soybean growers, with yield losses from 183,000 to 345,000 metric tons from 2006 to 2009 [6]. FLS has been observed more often in the southern USA, but it has been reported recently in the northern regions. Field experiments in the USA indicated that FLS can reduce yield from 17 to 31% if susceptible cultivars are used [7, 8]. A yield loss due to FLS of up to 60% was reported in Nigeria [9]. The most efficient disease management practices are foliar fungicide applications and host plant resistance, although other practices have also been recommended, including seed treatment with fungicides, crop rotation, and biological control using bacteria [10]. However, repeated use of fungicides can lead to reduced sensitivity in target populations. Strains of *C*. *sojina* resistant to strobilurin fungicides, the most commonly used fungicides against this disease, were found in several states in the USA [11, 12]. Thus, development and use of soybean cultivars with resistance to FLS is of great importance.

Studies on pathogenicity of *C*. *sojina* revealed a complex biological feature of this pathosystem. In Brazil and China, 22 and 14 races of *C*. *sojina* were reported, respectively [13, 14]. In the USA, five races of the fungus were classified in 1981 [15], but a study in 2004 showed that there are likely more than 12 different races of *C*. *sojina* found in various states [16]. Based on reactions that a collection of 93 *C*. *sojina* isolates from the USA (71), Brazil (15), and China (7) produced on a set of 38 soybean differential cultivars, Mian et al. (2008) proposed that there were at least 11 races of *C*. *sojina* [17]. Along with the characterization of the causal pathogen, identification and mapping of FLS resistance genes facilitates introgression of the genes into elite breeding lines.

Four single genes conditioning resistance to FLS are currently recognized by the Soybean Genetics Committee, though other resistance genes have been reported. The *Rcs*1 gene that confers resistance to race 1 of *C*. *sojina* was identified in ‘Lincoln’ [18]. A second resistance gene was found in ‘Kent’ and named *Rcs*2 due to its contribution to resistance to race 2 [19]. Soybean line ‘Davis’ carries *Rcs*3, which has been the most durable and robust gene [20]. The *Rcs3* allele not only confers resistance to race 5, but also to all other reported races in the USA as well as to Brazilian isolates [14, 20]. Two additional genes from PI 594891 and PI 594774 were approved by the Soybean Genetics Committee in 2012. These two single dominant resistance alleles had been identified and reported by Hoskins (2011), and were designated by the Soybean Genetics Committee as *Rcs*(PI 594891) and *Rcs*(PI 594774) [21]. Additionally, a dominant gene conferring resistance to Chinese race 7 has been referred to as *Rcs*7 [22], but this gene was not officially recognized by the Soybean Genetics Committee because of insufficient evidence to show that it is not allelic to the *Rcs*1, 2, and 3 genes. Although *Rcs*3 has been effective in soybean production, the complexity of the *C*. *sojina* races and breakdown of the FLS resistance genes have increased the need to identify additional resistance genes and pyramid those in elite germplasm.

Among the reported FLS resistance genes, *Rcs(*PI 594891) and *Rcs*(PI 594774) conferred a high level of resistance similar to that conditioned by the *Rcs*3 gene in 'Davis'. Furthermore, the resistance to FLS in PI 594891 and PI 594774 was controlled by two single dominant genes in close proximityon chromosome (Chr) 13, which is different from the *Rcs*3 allele on Chr18 [21]. The genomic regions harboring the resistance genes from these two PIs were found to be in a 3–4 Mbp interval that contains hundreds of genes. With Blackhawk and these two PIs being genotyped with SoySNP50k iSelect BeadChip [23], an enormous amount of SNP data obtained within the roughly mapped region could be used for fine-mapping. In addition, the utilization of KASP technology (LGC Genomics, Middlesex, UK), a closed-tube SNP detection method, has provided an effective platform to accelerate the genotyping process. The simplicity of the one-step procedure for data collection and small reaction volumes make this technology cost-effective, flexible, and the method of choice for a project that has numerous markers and samples.

The objectives of this study were to: 1) fine-map the genomic region containing *Rcs*(PI 594891) and *Rcs*(PI 594774) loci using the SoySNP50k Infinium chip data; 2) understand whether the two resistance loci located close to each other on the same chromosome with different symptoms (no lesion on PI 594891, small lesion on PI 594474) were different genes or the same resistance gene with different alleles; and 3) develop robust KASP marker assays to detect the *Rcs*(PI 594891/PI 594774) FLS resistance alleles for marker-assisted selection.

## Materials and Methods

### Population development

Crosses between the cultivar Blackhawk and the accessions PI 594891 and PI 594774 were made during the summer of 2003 at the Univ. of Georgia Plant Sciences Farm in Watkinsville, GA. PI 594891 and PI 594774 were found to possess resistance to a broad spectrum of *C*. *sojina* isolates, while Blackhawk is a highly susceptible parent [21]. F_2_ populations derived from F_1_ plants of both crosses were grown in a greenhouse at the Univ. of Georgia, Athens, GA in 2003 or 2004. Each population consisted of 200 F_2_ seed and was initially genotyped with SSR markers to obtain desired genotypes to advance to the F_2: 3_ generation. An additional 36 seed from the same population were used for KASP assay validation. For convenience, the F_2: 3_ populations of Blackhawk x PI 594891 and Blackhawk x PI 594774 were encoded as FLS-594891 and FLS-594774, respectively.

### Fine-mapping procedure

To fine-map the genomic region containing the resistance genes in PI 594891 and PI 594774, 200 F_2_ seeds from the cross of Blackhawk with either PI 594891 or PI 594774 were chipped into ¼ and ¾ seed pieces using razor blades. The ¼ and ¾ seed pieces were loaded into a micro-centrifuge tube and plate well, respectively, which were labelled with the same ID. DNA were extracted from the ¼ seed pieces and used for genotyping the F_2_ generation with SSR markers flanking the genomic region containing the FLS resistance genes. The ¾ seed pieces having PI homozygous alleles at one marker and heterozygous alleles at the other marker were selected and grown to obtain F_2: 3_ seed. At the same time, SNPs polymorphic between Blackhawk and PI 594891/PI 594774 from the genomic region bordered by two flanking SSR markers were identified and used for KASP assay development. The F_2: 3_ seed harvested were chipped again using the method described above. DNA from the ¼ seed chips were genotyped with KASP markers to identify individuals with recombination events in the target region. The ¾ seeds that possessed a recombination event were planted and used for phenotyping with *C*. *sojina* isolates in the greenhouse. Finally, genotype data (recombination breakpoints) and data on the reactions of all F_3_ plants to two *C*. *sojina* isolates were combined to narrow down the genomic regions containing FLS resistance genes in these two PIs.

### FLS Greenhouse Assays

PI 594774, PI 594891, Blackhawk, and F_2_ or F_3_ recombinant plants were evaluated in a greenhouse at the Univ. of Georgia Griffin campus using a protocol described previously [15, 17, 24] with some modifications. Seed were grown individually in 10-cm pots on a greenhouse bench. Seedlings were inoculated at the V2 to V3 growth stage of development [24]. A single trifoliolate of each plant was inoculated with isolates 21 and 23 of *C*. *sojina* by atomizing a conidial suspension of approximately 6.0 × 10^3^ spores mL^-1^. These two isolates were collected from China and classified as race 8 by Mian et al (2008). Because these two isolates belong to the same race, use of both isolates for inoculating F_2: 3_ plants provided confirmation of disease phenotypes. Inoculated plants were placed into a clear plastic bag for 48 h to maintain a high relative humidity. Plants were removed from bags and then placed on a greenhouse bench. Disease ratings were made 14 days after inoculation. The FLS reaction was scored as a qualitative trait (i.e. susceptible vs. resistant) for all plants. Plants were classified as susceptible when lesions were predominately large and spreading with light centers and dark margins. Plants were rated as resistant when they showed no lesions or predominately small lesions without clearly defined centers [17].

The F_2: 3_ recombinant plants were first inoculated with isolate 21 (race 8) of *C*. *sojina* [17]. After the phenotypic evaluation for this isolate was finished, all of the mature leaves with or without lesions in the experimental plants were excised. When the remaining young leaves matured, the plants were inoculated again with isolate 23 (race 8) of *C*. *sojina* [17].

For the haplotype experiment, 45 soybean lines and cultivars with diverse pedigrees were evaluated in the greenhouse at the Univ. of Georgia Griffin campus along with PI 594774, PI 594891, Davis, and Blackhawk. Three seed were planted per pot and four pots were used per genotype. Approximately one week after planting, seedlings were thinned to two plants per pot, which resulted in a total of eight plants per genotype for inoculation and rating. Two weeks after planting, the plants were inoculated with a mixture of isolates 21 and 23 (race 8) of *C*. *sojina*. Two weeks after inoculation, each plant was rated as susceptible, resistant, or immune.

### Genotyping F_2_ seed

Based on the study by Hoskins (2011), the flanking SSR markers Satt114 and Sct_033 on Chr13 were selected for genotyping F_2_ seed from the cross of Blackhawk x PI 594891, and Satt114 and Satt663 were used for the F_2_ seeds of Blackhawk x PI 594774. A ¼ seed chip was removed for DNA extraction from each of the F_2_ seed in both populations using a CTAB (Hexadecyltrimethylammonium bromide) procedure modified from Keim et al. [25]. The PCR reaction was conducted on a 384-well GeneAmp PCR System 9700 (PE-ABI, Foster City, CA) using fluorescent dye-labeled primers, following Diwan and Cregan’s protocol [26]. The marker fragments were analyzed with GeneMarker software (SoftGenetics, State College, PA).

### Sequencing genes identified from the fine-mapping work

Five genes identified from the 72.6 kb region using the FLS-594891 population were considered as candidate FLS resistance genes. The DNA sequences of these genes were obtained from the Phytozome.net website based on the sequence of ‘Williams 82’. Gene-specific primers for each gene were designed using the Primer3Plus program [27]. DNA from Blackhawk and the two PIs was isolated from leaf tissue using the CTAB protocol, and 5–50 ng of DNA were used in PCRs with gene-specific primers for each gene of interest under the following conditions: 95°C for 5 min, followed by 34 cycles of 95°C for 30 s, 60°C for 30 s, and 72°C for 1 min per 1 kb of predicted product size. After PCR, the target sequence amplification was verified on a 1.5% agarose gel by electrophoresis and then sequenced at the Georgia Genomics Facility located on the Univ. of Georgia Athens campus. Sequence traces were assembled and manually evaluated for polymorphisms using Geneious software (version 5.5.7.). Putative polymorphisms were confirmed by an independent sequencing reaction.

### KASP assay development

KASP primer sequences were designed using PrimerExpress (Life Technologies, Carlsbad, CA), and are listed in S1 Table. KASP reactions were 4 μL in volume, with 2 μL of 2X premade KASP master mix (LGC, Middlesex, UK), 0.055 μL of primers mix (Sigma-Aldrich, St. Louis, USA), and 10–40 ng of genomic DNA. After PCR amplification, PCR plates were scanned with a Tecan M1000 Pro Infinite Reader (Tecan Group Ltd., Männedorf, Switzerland). The SNP genotype was determined using Kluster Caller software (LGC Genomics, Middlesex, UK).

To test the association between the SNPs identified in the two candidate genes Glyma13g25340 and Glyma13g25350 and the FLS phenotypes, and to develop robust SNP assays for genotyping and marker-assisted selection, one SNP specific for both PI 594891 and PI 594774 from each of the two candidate genes was selected for KASP assay development, This resulted in a total of four KASP assays. A set of 36 F_2_ seed from either Blackhawk x PI 594891 or Blackhawk x PI 594774 and a panel of 158 cultivars and germplasm lines were genotyped using these KASP assays. The 158 soybean lines in the panel were selected due to the availability of DNA for the genotyping experiment and they were from the list used in a previous experiment in our lab. Due to the limited availability of seeds, only 45 lines were assayed for their reactions to *C*. *sojina*.

### Inoculation for RT-qPCR assays

The FLS phenotyping was conducted in a greenhouse at the Univ. of Georgia, Griffin campus using procedures described by Mian et al. [17] with some modifications. A total of 100 seed for each of the three parents (Blackhawk, PI 594891, and PI 594774) were planted in eight flats which were divided into two sets. Within each flat, 12 10-cm square plastic pots, four for each of the parents (Blackhawk, PI 594891, and PI 594774), were planted using a randomized complete block design. Two to three seeds were planted in each pot and then seedlings were thinned to one plant at the V2-V3 stage [25]. Four flats in the first set were inoculated with *C*. *sojina* isolate 21, while four flats in the second set were mock-inoculated with water as a control. At 3, 6, 11, and 16 days after inoculation (dai), unifoliate leaves from one inoculated plant of each genotype were excised from the stem, placed in sealable plastic bags, frozen in dry ice, and then stored in a -80°C freezer.

### qPCR analysis and cDNA cloning of candidate genes

qRT-PCR was carried out using the LightCycler 480 Real-Time PCR System (Roche, Germany). Specific primers for each candidate gene were designed using Primer3Plus software [28]. PCR products amplified from these primers were sequenced to determine if the correct genes of interest were amplified. Only primer pairs that gave a single amplicon were selected for qRT-PCR. Primer efficiency was determined using five-fold serial dilutions using DNA of a soybean line S03-380RR. RNA was extracted using TRIZOL RNA extraction reagent by following the procedure from the manufacturer (Life Technologies, Carlsbad, CA). The qRT-PCR reactions were conducted with three technical replications in 10 μl reactions for each biological replicate using the GoTag 1-Step RT-qPCR Kit (Promega Corporation, Madison, WI). Following the reverse transcriptase reaction, amplification was conducted at 95°C for 10 min, then 35 cycles of 95°C for 10 s, 60°C for 20 s, and 72°C for 20 s. Soybean gene *cons7* was used as an internal control [28]. The quantification of gene expression was performed using the relative ΔΔCT method [29]. cDNA cloning and sequencing was conducted using the QIAGEN OneStep RT-PCR Kit (Qiagen, Valencia CA) and specific primers for each gene.

### Haplotype analysis

**Fig 1:**
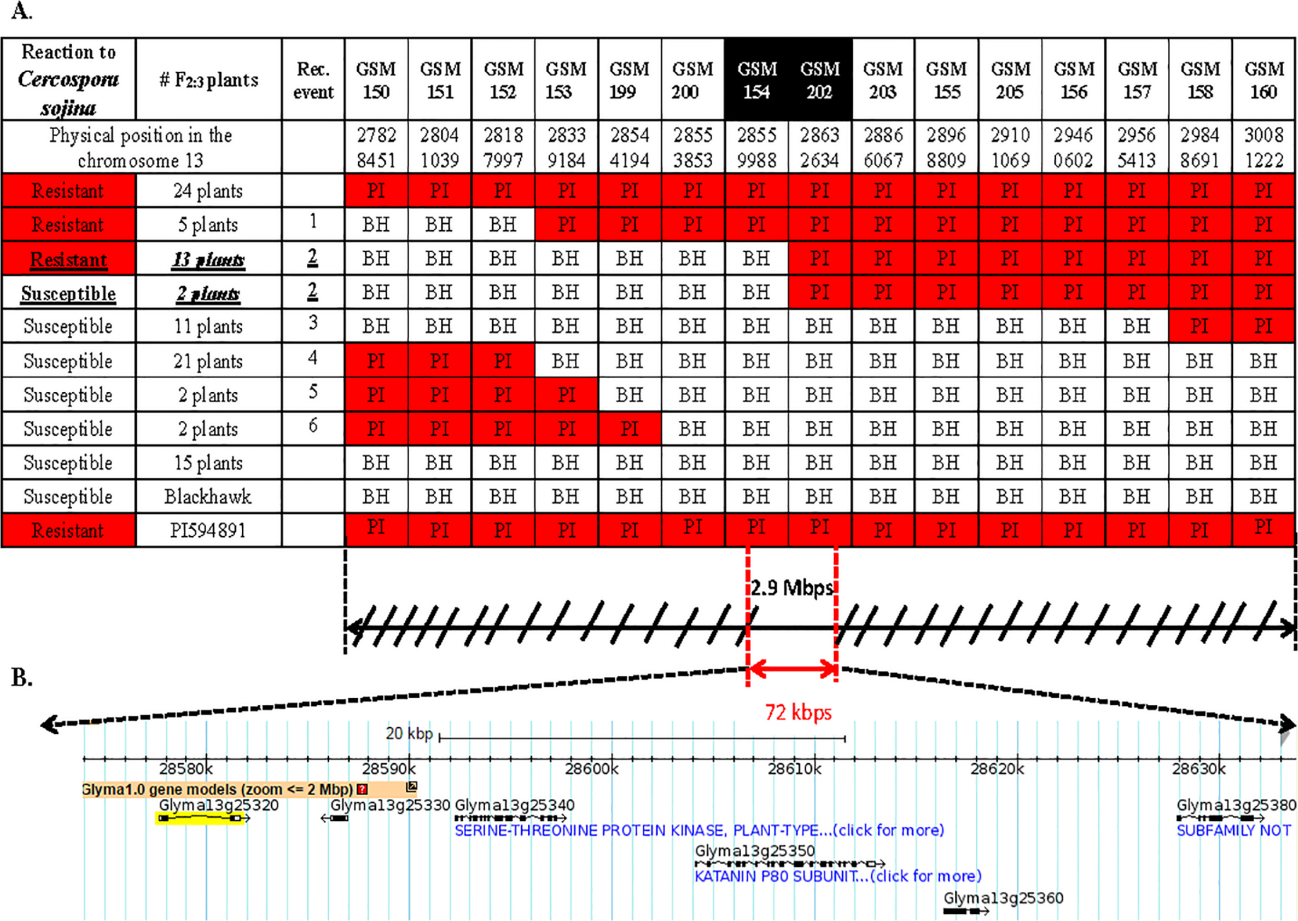
SNP genotypes at 15 SNP loci and the reaction of F_2:3_ plants with recombination events to *Cercospora sojina* (race 8) in a Blackhawk x PI 594891 population. **A.** Genotypes at 15 SNP marker loci and the reactions of F_**2:3**_ plants with recombination events to *Cercospora sojina* in the population of Blackhawk x PI 594891. The 15 plants that were highlighted and underlined had the same recombination breakpoint (No. 2), but opposite phenotypes (BH = Blackhawk allele; PI = PI 594891 allele; Het = heterozygous alleles). **B**. Screen shot of Williams 82 genome (assembly version 1.01) (http://SoyBase.org), displaying a 72.6 kb region on chromosome 13 which contained the locus responsible for the resistance in PI 594891.

**Fig 2:**
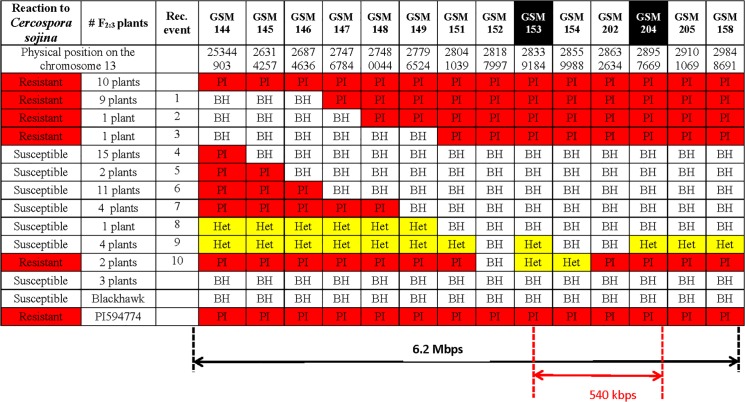
SNP genotypes at the 14 SNP marker loci and the reaction of F_2:3_ plants with recombination events to *Cercospora sojina* (race 8) in a population from Blackhawk x PI 594774. (BH = Blackhawk allele; PI = PI 594774 allele; Het = heterozygous alleles).

To understand the genetic variation at resistance loci, haplotype analysis was performed at the fine-mapped locations of the resistance genes in PI 594774, PI 594891, and Davis (*Rcs3*). PI 594774, PI 594891, Davis, Blackhawk and 45 soybean lines and cultivars with diverse pedigrees were used in the haplotype analysis. The phenotypic data of these lines were obtained in the greenhouse at the Univ. of Georgia Griffin greenhouse facility. SoySNP50K Infinium Chip data were obtained at Soybase (www.soybase.org) for all lines with the exception of G00-3213, G00-3880, N05-7432, N7002, N77-114, and N8001, which were fingerprinted using the SoySNP50K Infinium BeadChip at Michigan State Univ. For PI 594891 and PI 594774, the haplotype windows were defined by the 72.6 kb fine-mapped region (Figs 1 and 2). The haplotype window for Davis was defined by the interval based on the primer sequence of Satt244) and AZ573TA150 markers used to map the *Rcs3* gene on chr16 [30]. Therefore, each interval was based on the estimated physical locations of the SSR or SNP markers in the soybean genome map available at www.soybase.org using the Glyma1.01 map version. Polymorphic SNP markers from the SoySNP50K Infinium BeadChip of all soybean lines and cultivars within the defined intervals were used for haplotype analysis. The graphic haplotype allele visualization of all lines compared to PI 594774, PI 594891, or Davis was performed, and the similarity for a given genotype to the target line was calculated using Flapjack software [31].

## Results

### The 2.9 Mbp genomic region associated with FLS resistance in PI 594891

A total of 192 F_2_ seed derived from Blackhawk x PI594891 was chipped and DNA from these F_2_ seed was used for genotyping with SSR markers Satt114 and Sct_033. Based on the genotyping results, 14 F_2_ seed were found to carry PI 594891 alleles at one marker and to be segregating at the other (PI-HET or HET-PI, with PI and HET standing for homozygous and heterozygous alleles from PI 594891, respectively). There were also nine seed with the genotype BH-HET or HET-BH from this population (BH indicates the Blackhawk alleles). These 23 seed were grown to maturity in the greenhouse, and each of the F_2_ plants produced 30 to170 F_3_ seeds, with a total of 1,544 seeds. All of the seeds were chipped for the next cycle of genotyping.

Comparison of the SoySNP50K Infinium BeadChip data between Blackhawk and PI 594891 in the 2.9 Mb region bordered by the Satt114 and Sct_033 markers identified 91 single nucleotide polymorphisms (SNPs). However, these SNPs were not evenly spaced, and often occurred in clusters of 2–14 SNPs. Of these 91 SNPs, 31 were in the regions unfavorable for designing KASP assays. Eleven SNPs residing in regions suitable for KASP primer design, and spaced approximately every 250 kb throughout the targeted region, were used to develop KASP assays designated as GSM150 to GSM160. After the phenotyping data from the seedlings of recombinant F_2: 3_ seeds were obtained, 13 additional SNP assays were designed in the second round to saturate and narrow down the region containing the resistance gene *Rcs*(PI 594891).

Of 1,544 F_2: 3_ seeds genotyped with markers GSM150 through GSM160, 259 F_2: 3_ seeds were found to carry one or two recombination events. Due to limited greenhouse space, only 157 seeds were selected for planting, inoculation, and evaluation with *C*. *sojina* isolates 21 and 23 in March-April 2013, respectively. In the second evaluation, 55 seed of this population were then grown and phenotyped with the same isolates in May-June 2013. DNA samples extracted from the leaf tissues of these 212 phenotyped F_2: 3_ plants were genotyped with all 24 KASP assays. Finally, the region containing the resistance gene in PI 594891 was narrowed down using the data of 66 F_2: 3_ recombinant plants harboring six recombination break points (Fig 1). The phenotypes of FLS for these F_2: 3_ plants shown in Fig 1 are the reactions to both *C*. *sojina* isolates.

PI 594891 plants and 24 F_2: 3_ plants with the genotype homozygous for PI 594891 alleles in the interval between GSM150 and GSM160 were resistant to FLS, and Blackhawk and 15 plants with the Blackhawk haplotype were observed as susceptible (Fig 1). In total, six recombination break points were found among these 66 F_2: 3_ plants. The region containing the resistance gene(s) to FLS in PI 594891 was fine-mapped in a set of 15 plants that have the same recombination breakpoint (No. 2 in Fig 1) but opposite phenotypes (highlighted in bold and underlined in Fig 1). These 15 F_2: 3_ plants contained a recombination breakpoint between GSM154 and GSM202. However, 13 F_2: 3_ plants with this genotype showed a resistance reaction to *C*. *sojina* isolates 21 and 23, suggesting that the resistance gene(s) did not reside on the left side of GSM154. In contrast, two F_2: 3_ plants with this genotype and susceptible reactions to both isolates, indicating that the resistance gene(s) should not be located in the region bordered to the left by GSM202. Altogether, the genotyping and phenotyping data of these 15 plants indicated that the *Rcs*(PI 594891) gene is located in a region of approximately 72.6 kb between the SNP markers GSM154 and GSM202. This region is predicted to harbor five genes, three of which have a predicted function obtained from the Phytozome.net database based on protein sequence and high similarity with known genes from other species. The first known gene is Glyma13g25340, and was annotated to be an STRUBBELIG-receptor like protein with leucine rich repeats. The second known gene is Glyma13g25350, which was predicted to be transducin or Pleiotropic regulatory gene. The third gene, Glyma13g25380 was annotated to be a FALZ-Related Bromodomain-containing protein. Two remaining genes, Glyma13g25320 and Glyma13g25331, encode proteins with unknown functions. The three genes with annotated functions are referred to as Glyma13g25340 (LRR), Glyma13g25350 (TPR), and Glyma13g25380 (FBP).

### The 3.3 Mbp genomic region associated with FLS resistance in PI 594774

The same fine-mapping strategy was employed for the population from Blackhawk × PI 594774. In this population, 17 F_2_ seed with genotypes PI-HET or HET-PI for SSR markers Satt663 and Satt114 were identified from 192 F_2_ seed and advanced to the F_3_ generation. At harvest, 1,372 F_2: 3_ seeds were obtained and the seeds were chipped to ¼ and ¾ seeds for genotyping and advancement.

Using the SoySNP50K Infinium chip data for Blackhawk and PI 594774, 33 SNPs were identified in the 3.3 Mb region flanked by Satt663 and Satt114. However, most of these SNPs (22) were clustered together, and some SNPs fell in a region that is unfavorable for primer design. Therefore, only six SNPs, designated as GSM144 to GSM149, were successfully designed as KASP assays. The performance of these KASP assays was tested using DNA from leaves of the two parents and a set of 36 F_2_ individuals from Blackhawk x PI 594774.

DNA was extracted from the chips of 1,372 F_2: 3_ seed and genotyped with the six SNPs. Of 1,372 F_2: 3_ seed, we identified 395 seed that carried one or two recombination events in the interval flanked by Satt663 and Satt114. In March-April 2013, 55 seeds with recombination events from this population, along with the seeds from the FLS-594891 population were grown in the greenhouse and were phenotyped with *C*. *sojina* isolates 21 and 23. In the second evaluation in May-June 2013, 124 seed were grown and evaluated for FLS reaction. Due to germination issues, phenotypic data were only collected for a total of 103 F_2: 3_ plants from both evaluations. Leaf DNA collected from these 103 F_2: 3_ plants were genotyped with a set of six KASP markers.

The resistance gene from PI 594774 was initially mapped to a region of Chr 13 adjacent to the one in PI 594891, and was bordered by Satt663 and Satt114 [21]. The physical locations of these two SSR markers are close to KASP markers GSM144 and GSM149, respectively. However, fine-mapping data showed that the resistance gene(s) did not reside in this region (clearly indicated by the plants with recombination event 3 and 7). Since the resistance gene(s) in PI 594774 and PI 594891 were lying in two adjacent regions on the same chromosome, DNA of the 103 F_2: 3_ plants of the population FLS-594774 was genotyped with eight KASP markers from the population FLS-594891 (these markers are also polymorphic for Blackhawk and PI 594774). Data for 63 F_2: 3_ plants from the FLS-594774 population and 14 KASP markers covering a region of 6.2 Mbps are presented in Fig 2. Each recombination break point was numbered for ease of explanation. The reactions to *C*. *sojina* of the F_2: 3_ plants in Fig 2 were for the same for the isolates 21 and 23 of *C*. *sojina*.

In this population, PI 594774 and 10 plants with PI 594774 alleles at all of the KASP markers showed a resistance reaction, while Blackhawk and three plants with a genotype resembling Blackhawk showed a susceptible reaction (Fig 2). Representing recombination event No. 9 were four F_2: 3_ plants that were homozygous for the Blackhawk allele at SNP markers GSM152, GSM154, and GSM202, and heterozygous for all other SNPs in the region. Because these four plants had a susceptible reaction with both isolates of *C*. *sojina*, the FLS resistance gene from PI 594774 was predicted to be in one of two regions: one flanked by GSM151 and GSM153 or one flanked by GSM153 and GSM204. However, there were two F_2: 3_ plants that were homozygous for the Blackhawk allele with GSM152 and PI-HET for all other KASP markers (recombination event No. 10). They were also resistant to both FLS isolates. Therefore, the resistance gene in PI 594774 was estimated to reside in the region of approximately 540 kb flanked by GSM153 and GSM204.

### Relative expression levels of candidates genes in the presence of FLS pathogens

**Fig 3:**
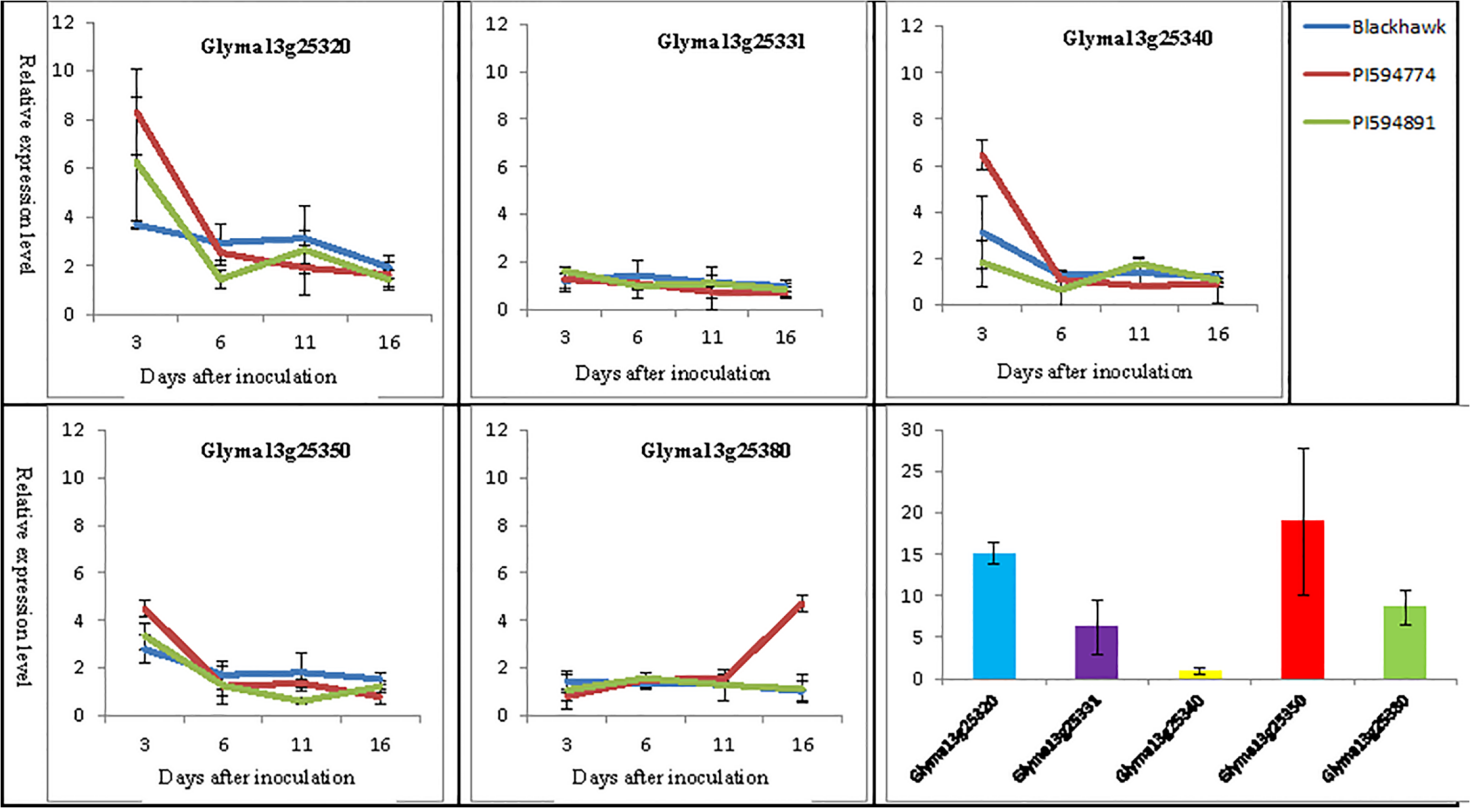
Relative expression level of five candidate genes identified in PI 594891 and PI 594774 in the presence of the frogeye leaf spot fungus *Cercospora sojina*.

To further pinpoint the candidate genes that contribute to the resistance to *C*. *sojina* in both PI 594891 and PI 594774, a real-time quantitative PCR experiment was performed to examine the expression levels of five candidate genes within the 72.6 kb region fine-mapped in the FLS-594891 population. Blackhawk and the two PIs were inoculated with *C*. *sojina* isolate 21 (Fig 3). Leaves for each parent were sampled at 3, 6, 11, and 16 days after inoculation (dai). Uninoculated leaves from the three parents were also sampled, but were not used for the assays.

Blackhawk, PI 594891 and PI 594774 had the same steady expression level at all-time points for the gene Glyma13g25331 (Fig 3). A similar trend was also observed for Glyma13g25380 (FBP), except that at 16 dai expression of this gene was 2× higher in PI 594774 than in the other two parents. The expression levels of Glyma13g25320, Glyma13g25340 (LRR), and Glyma13g25350 (TPR) were 2× and 1× higher in PI 594774 and PI 594891 than in Blackhawk, respectively, but only at 3 dai. There were no differences in the expression levels of these genes among the three genotypes at the later time points. The difference in expression levels of the three genes at 3 dai between PI 594774 and Blackhawk was statistically significant, while there was no difference between PI 594891 and Blackhawk. Because the expression levels of the five candidate genes in the two PIs differed from those in Blackhawk only at 3 dai, the expression of the five genes was compared in Blackhawk leaf tissues at 3 dai. At this time point, the expression levels of genes Glyma13g25331 and Glyma13g25380 (FBP) were about 7–8 times higher than those of Glyma13g25340 (LRR), which had the lowest expression level. Tthe Glyma13g25320 and Glyma13g25350 (TPR) genes had expression levels which were 15-18x higher than that of Glyma13g25340 (LRR) (Fig 3).

### Sequencing cDNA and DNA of candidate genes within the fine-mapped genomic regions

To further characterize the five candidate genes identified in PI 594891 and PI 594774, cDNA of the five genes was sequenced using RNA from the RT-qPCR experiment. When RNA extracted from leaves of Blackhawk and two PIs at 3 dai was used for cDNA synthesis, no PCR products were obtained for two genes Glyma13g25340 (LRR) and Glyma13g25380 (TPR). This supported the RT-qPCR results for Glyma13g25340 (LRR), which indicates that it was weakly translated. Glyma13g25380 had no difference in expression levels among Blackhawk and the two PIs until 16 dai, so no attempt was made to further sequence cDNA of this gene.

Sequencing the cDNA products of Blackhawk and two PIs for Glyma13g25320 indicated a SNP A^448^G in mRNA of PI 594891 that resulted in an amino acid change of threonine (T)^150^ to alanine (A). This amino acid substitution was evaluated as tolerant by the algorithm “Sorting Intolerant from Tolerant” (SIFT) [32] because the SIFT probability for the change T^150^A was equal to 0.64 (amino acids with P <. 05 are predicted to be deleterious). In addition, the presence of both threonine and alanine was observed at this residue in Weblogo images [33], indicating that this amino acid change in Glyma13g25320 protein was also observed in other species (S2 Table).

For Glyma13g25331, PI 594891 had the same sequences as Williams 82, while five SNPs were identified in cDNA sequences of Blackhawk and PI 594774 including C^178^A (H^60^N), A^446^T (N^149^I), C^548^A (S^183^Y), A^657^C, and T^662^C (I^221^T). An attempt was made to evaluate the impact of the amino acid change in this gene for Blackhawk and PI 594774, but blasting SIFT did not result in any sequences. In addition, the RT-qPCR results showed no difference at any time point for this gene in Blackhawk, PI 594891 and PI 594774, so there was no further work done with this gene.

Because no products were obtained in cDNA synthesis for the Glyma13g25340 (LRR) gene, the region consisting of 7.6 kb of genomic DNA and 5 kb of promoter was sequenced for Blackhawk, PI 594891, and PI 594774. Compared to the sequence of Williams 82, there was a total of 15 genotype-specific SNPs and nine common SNPs found in Blackhawk, PI 594891, and PI 594774 (S2 Table). Four of the SNPs were found in Blackhawk, including two SNPs in the promoter, one in the intronic region, and one in the exon that resulted in an amino acid change of L^636^F. This amino acid substitution was evaluated as deleterious by SIFT (*P* = 0.03). For PI 594774, there were two SNPs in the intronic region, two in the exonic region (only one resulted in an amino acid replacement of S^126^T), and one SNP in the 3’UTR region. The amino acid change in PI 594774 was predicted to be tolerant by SIFT. Compared to Blackhawk’s sequence, PI 594891 had a 6 bp deletion in the last intron and a SNP in the 3’ UTR region. Among nine common DNA changes for all three genotypes there was a big deletion of 602 bp and an insertion of 161 bps in the 12^th^ intron. In addition to this deletion/insertion, there was one SNP in the promoter, five in the intronic region, and one in the exonic region. However, none resulted in any amino acid change. These SNPs as well as one in the 3’ UTR region were observed in all three genotypes. In the 5 kb promoter region, there were 30 changes in DNA sequences of Blackhawk, PI 594891, and PI 594774 including SNPs, insertions and deletions when compared to that of Williams 82. Among the identified SNPs, one, five, and six SNPs are unique for PI 594891, Blackhawk and PI 594774, respectively. There were four SNPs shared between Blackhawk and PI 594891, 10 SNPs shared between Blackhawk and PI 594774, and four common to all three genotypes (S2 Table). The sequence of PI 594891 for this region was conserved and had fewer differences from that of Williams 82 in comparison to those of Blackhawk and PI 594774.

In the 8.8 kb genomic DNA sequence of Glyma13g25350 (TPL), there were 123 SNPs identified in Blackhawk, PI 594891, and PI 594774 when compared to the sequence of Williams 82. Of these 123 SNPs, there were 12 SNPs common to all three genotypes, with two of them in exonic regions, resulting in one common amino acid change of F^245^L. Blackhawk and PI 594891 had 83 SNPs in common, including 74 SNPs in introns and nine in exons that caused six amino acid changes. However, all of the amino acid changes were predicted to be tolerable by SIFT. Compared to the Blackhawk sequence, PI 594891 had eight SNPs in the intron and one in the 5’ UTR region, and PI 594774 had 13 genotype-specific SNPs in the intron, three in exons and one in the 5’ UTR region. All of the SNPs in the exonic regions in PI 594774 resulted in amino acid changes, with two changes predicted to be benign and one, E^646^K, predicted to be deleterious for the function by the SIFT program (*P* = 0.02). The 5 kb promoter region of this gene was sequenced and divided into two segments based on the pattern of the SNPs found in the three genotypes. In the 1,500 bp promoter region upstream and adjacent to the 5’ UTR region, there were six, two, and one SNP(s) that were specific to PI 594774, PI 594891, and Blackhawk, respectively. There was also one SNP that was present in all three genotypes. In this region, the Blackhawk and PI 594774 sequences shared the highest similarity, with 30 common SNPs and one deletion/insertion. When the 3.5 kb region upstream of this 1.5 kb promoter was sequenced, it was found that Blackhawk had the greatest number of differences in DNA sequence, including 14 SNPs and three deletions of 2, 5, and 6 bp. There were two SNPs that were shared between PI 594774 and PI 594891, two common to Blackhawk and PI 594891, and one common to Blackhawk and PI 594774. There were four and nine SNPs that are specific to PI 594891 and PI 594774, respectively. Three common SNPs were seen in all of the three genotypes in this upstream region of the promoter.

### KASP assay development

**Fig 4:**
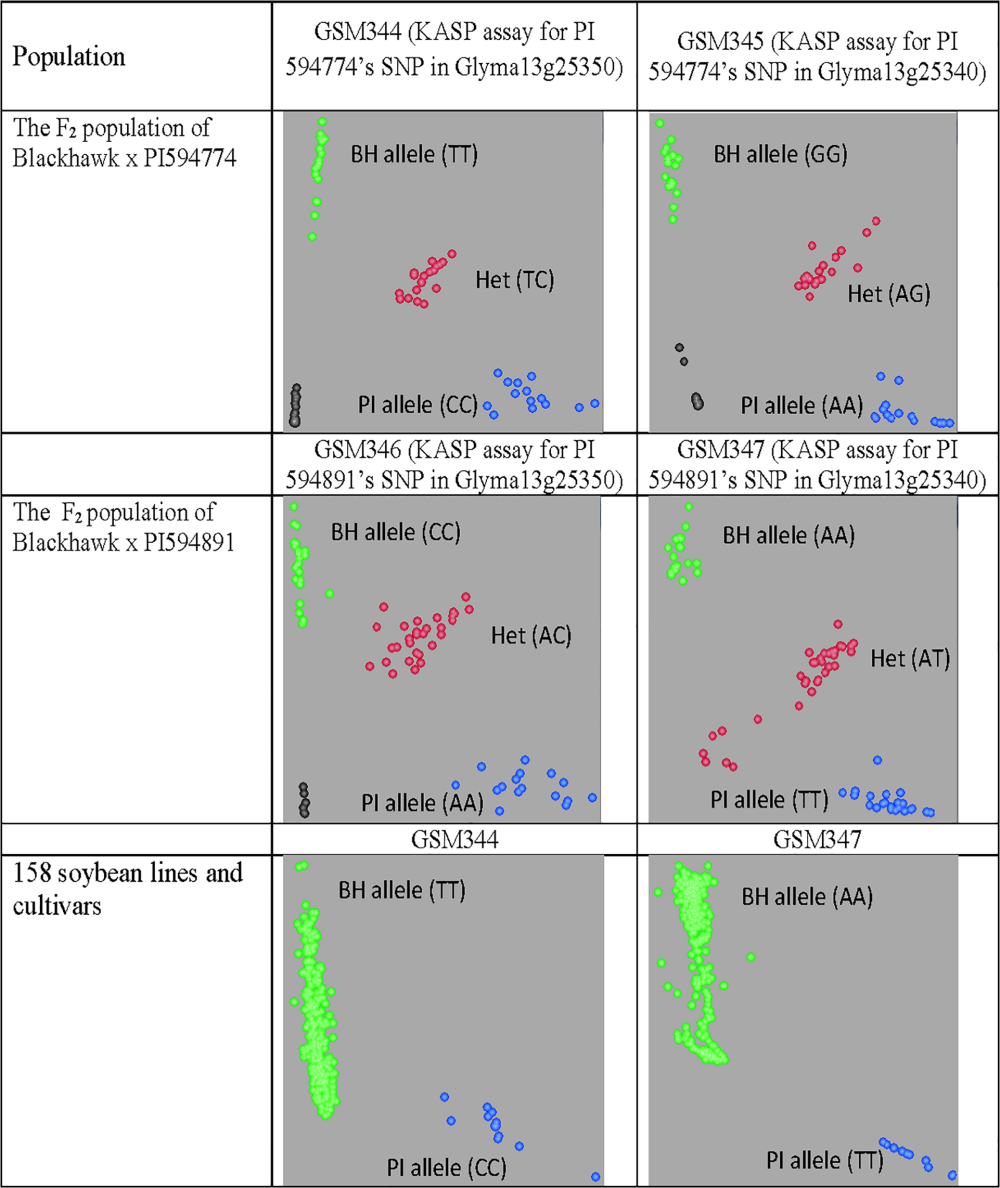
SNP graphs of KASP assays for the F_2_ populations of Blackhawk x PI 594891/PI 594774 and 158 soybean lines and cultivars. (BH = Blackhawk allele; PI = PI 594774 allele; Het = heterozygous alleles).

For the STRUBBELIG-receptor like gene (Glyma13g25340), the SNP G^18^A in the exonic region of PI 594774 and A>T in the 5’UTR region of PI 594891 were used for KASP development. For the transducin-like gene (Glyma13g25350), the SNP G^1828^A (causing an amino acid change) and SNP C^-382^>A (in the promoter) were used for KASP development. These KASP assays were named GSM344-347 (S1 Table). The performance and accuracy of these SNPs in predicting the FLS reaction were initially tested using DNA of Blackhawk, the two PIs, and a set of 36 F_2_ individuals for each of the populations FLS-594891 and FLS-594774. The phenotype of these F_2_ individuals was evaluated in the greenhouse in 2012. The tight clustering of the three genotypic classes for each marker indicated that they can effectively differentiate different genotypes (Fig 4). A complete association was observed between SNP alleles and reaction to FLS in the two sets of F_2_ individuals when each was run with two SNP markers (success rate 100%), which demonstrated the robustness of these SNP markers to predict the FLS phenotypes. These four SNPs were also used to genotype F_2: 3_ individuals which were used for fine-mapping genomic regions in both FLS-594891 and FLS-594774 populations. The PI alleles from two PIs were always found in individuals with the resistant phenotype.

### Haplotype analysis with additional soybean lines and cultivars

When GSM344 and GSM347 assays were used to genotype a panel of 45 soybean cultivars and lines with known phenotypes, both PI 594891 and PI 594774 showed unique haplotype alleles at these marker loci (S3 Table). To understand the genetic variation at this fine-mapped region, a haplotype and phenotype analysis was performed at the mapped locations of the resistance genes in PI 594774, PI 594891, and Davis (*Rcs3*) for these 45 lines.

The source of inoculum used was a combination of isolates 21 and 23 (race 8) of *C*. *sojina*. PI 594774 had an immune reaction, in which no lesions were visible. PI 594891 had a resistance reaction which was indicated by predominately small lesions without clearly defined centers. The Davis plants had a combination of immune and resistance reactions, while Blackhawk was susceptible (large lesions spreading with light centers and dark margins). Of the 45 soybean lines and cultivars, 17 had a combination of immune and resistance reactions on the assayed plants, 12 had an immune reaction, five had a resistance reaction, and 11 had a susceptible reaction (S3 Table).

The haplotype window defined was based on the fine-mapping of the resistance genes to a 72.6 kb region with 11 SNP markers, with physical positions of the first and last SNPs being Gm13_28559988 and Gm13_28632634, respectively. In this interval both PI 594774 and PI 594891 have unique haplotypes which are different from those of Davis, Blackhawk, and the 45 soybean cultivars and lines (S4 Table). We have also evaluated the haplotype allele variation at the *Rcs3* locus from Davis based on the mapped interval of the *Rcs3* gene on chr16 [31]. The physical positions of the first and last SNP in the Davis haplotype window were Gm16_32681330 and Gm16_33360539, and there were a total of 30 SNPs in the defined window (S5 Table). Similarly, Davis had a unique haplotype from PI 594774, PI 594891, and Blackhawk. However, there were four cultivars ‘Young’, ‘Cook’, ‘Doles’, and ‘N6201’ with a 100% match to the Davis haplotype (S5 Table), suggesting that these cultivars all have Davis in their pedigrees.

## Discussion

The emergence and spread of strains of *C*. *sojina* K. Hara with tolerance to some commonly used fungicides, the breakdown of known FLS resistance genes, and the complexity of the FLS race structure have made the development of FLS-resistant soybean cultivars an important objective. The study by Hoskins (2011) indicated that the FLS resistance gene(s) in PI 594891 and PI 594774 to *C*. *sojina* (race 8) were located at a different locus compared to the *Rcs3* gene in ‘Davis’, providing an opportunity for gene pyramiding to obtain a more durable and stable resistance against this disease [21]. The small number of candidate genes identified from fine-mapping would enhance the feasibility of map-based cloning of novel FLS resistance genes in PI 594891 and PI 594774.

PI 495891 and PI 594774 were found having resistance reactions to 12 isolates representing all of the 12 races in a differential experiment (data not shown). In this experiment, only one race (race 8) was used as the one that was used in the QTL mapping study conducted previously to identify the resistance genes in these two PIs. As *Rcs3* was found to condition resistance to all known races of *C*. *sojina*, it is likely that the genes controlling the resistance to other *C*. *sojina* isolates in these two PIs are the same gene(s). However, this question can only be answered by phenotyping the F_3_ recombinant plants generated from this experiment with other isolates of the remaining 11 races.

This study has demonstrated a novel approach to fine-map genomic regions containing genes of interest using the SoySNP50K Infinium chip and KASP technology for accelerated genotyping. Three key factors typically affect fine-mapping studies: marker density, recombination frequency, and accuracy of recombinant phenotypes [34]. Most fine-mapping studies in plants have used SSR markers for genotyping and identification of recombination events [35–39]. Although numerous SSR markers are now available [40], deployment of SSR markers for genotyping a large number of seeds is costly, time consuming, and laborious. This study took advantage of the available SoySNP50K Infinium BeadChip data and selected polymorphic SNPs based on their locations and sequences, followed by KASP marker assay development. This strategy provided us with more markers and flexibility over the genotyping process. Ninety-one SNPs were identified for Blackhawk and PI 594891 in a 2.9 Mbp-genomic region, with an average of three SNPs per 100 kb. For Blackhawk and PI 594774, the frequency is much lower, with one SNP per 100 kb. With a total of 30 SNPs being developed as KASP marker assays and nearly 4,000 samples being screened, KASP technology became the method of choice for this study. The small reaction volume (4 μL) and simplicity of PCR implementation and data acquisition made the entire genotyping process affordable, fast, and accurate. In addition, use of seed chipping saved us a lot of time, space, and labor that would have been needed for planting the entire F_2: 3_ populations. It also increased the number of F_2: 3_ seeds that can be phenotypically screened and enhanced the probability of finding lines with recombination events. Altogether, the combination of high SNP marker density, seed chipping method, and an inexpensive and fast genotyping platform made our fine-mapping study successful in a reduced period of time.

From this study, *Rcs*(PI 594891) was fine-mapped to a 72.6 kb interval based on the opposite phenotype of plants carrying a same recombination breakpoint. The FLS resistance gene(s) in PI 594774 were found to be not located within a 3.3 Mbp region bordered by Satt663 and Satt114 in the previous study, but in a 540 kb region inside the 2.9 Mbp region containing the FLS resistance gene(s) in PI 594891 [21]. In the FLS-594774 population, fewer (103 plants) F_2: 3_ plants were identified when compared to the FLS-594891 population. This is due to many factors including the limited number of polymorphic SSR markers and the low recombination frequency in this region. Six plants carrying interesting crossovers were identified and used to narrow down the region containing *Rcs*(PI 594774) to a region of 540 kb. Because this region overlaps with the 72.6 kb region found for PI 594891, all of the candidate genes in the 72.6 kb regions were characterized for both PIs. Sequencing and qPCR data of these candidate genes in PI 594774 suggested that three genes in this region were more highly expressed in PI 594774 than in Blackhawk following infection with *C*. *sojina* race 8 isolates, and that one of the genes contained a deleterious mutation. Continuing the fine-mapping effort with larger populations and more advanced generation materials may be implemented to further narrow down the 540 kb region found in this study for *Rcs*(PI 594774). Extensive sequencing is also needed to provide more SNPs for better resolution of recombination events if this is the method of choice. Functional characterization of these gene(s) in the two PIs (silencing or complementation) is another feasible approach to verify the true cause of the FLS resistance carried in these two PIs.

In this study, RT-PCR was conducted to measure the expression of five candidate genes from the fine-mapped genomic region in PI 594891 at four time points over two weeks which is the necessary time for the lesions to be seen. However, the data indicated that only at three days after inoculation, the expression of these five genes differed between the three genotypes. This suggests that the period from 0–3 days after inoculation is a critical phase to investigate the expression level of candidate genes that control the FLS resistance in soybean. This is different from the pattern of candidate gene(s) in response to *Cercospora zeae-maydis* or *Cercospora beticola* causing leaf spot diseases in maize and sugar beet, respectively. When maize and sugar beet were inoculated with the two aforementioned *Cercospora* species, resistance-related genes showed the most difference in their expression levels at 7–15 dai [42, 43]. Sequencing the 72.6 kb region between Blackhawk and PI 594891 may provide the most accurate information about the number of genes present in this region.

The difference in the SNP and expression profiles of the five candidate genes residing in the 72.6 kb fine-mapped interval were different between two PIs, suggesting that the FLS resistance might be controlled by two different gene(s) residing in the fine-mapped regions or different alleles of the same gene. In addition, we have to take into account that the FLS resistance gene in PI 594774 may reside outside of the 72.6 kb interval. Additional phenotypic data on these lines in a study where they were compared to 45 additional soybean lines and cultivars using a combination of FLS isolates 21 and 23 indicated a difference in phenotypic reactions which fortified this hypothesis. PI 594774 had an immune reaction, while PI 594891 had resistance-type lesions (S3 Table). Haplotype analysis also indicated that these two PIs may have different resistance alleles. In the haplotype window, both PIs had a unique haplotype (55% similarity), which was also different from the haplotypes of all other tested cultivars and elite lines, suggesting that PI 594774 may carry a different resistance allele than PI 594891. The haplotype of Davis was unique from those of PI 594774, PI 594891, and the susceptible parent, Blackhawk, but identical to four cultivars (Young, Cook, Doles, and N6201) based on 30 polymorphic SNP markers in the defined haplotype window. Young was developed by the USDA-ARS in North Carolina, and is a cross of Davis x ‘Essex’ [41]. Cook and Doles were both developed at the Univ. of Georgia and are derived from the crosses of ‘Braxton’ x Young and D74-7741 x Young, respectively. Cook is resistant to the common races of *C*. *sojina* and Doles is resistant to all known races in the U.S. [42, 43]. N6201 was also released by USDA-ARS in North Carolina and is a cross of ‘Nakasennari’ x Young. It is also resistant to *C*. *sojina* [44]. Based on this haplotype analysis, whether intentional or accidental, the *Rcs3* gene identified in the cultivar Davis may also reside in these four cultivars. With such large haplotype allele variation, it could be inferred that the other lines that are resistant to *C*. *sojina* could carry genes different than *Rcs3* and resistance alleles from PI 594774 and PI 594891. Further studies are planned to identify possible additional new resistance sources.

The region on chr13 surrounding Satt114 marker is a resistance gene-rich region. The resistance gene *Rsp8* conditioning the resistance to *P*. *sojae* isolate OH25 was positioned between the markers Satt425 and Satt114 on chr13 (Satt114 was found to be significantly associated with FLS resistance in PI 594891 and PI 594774) [45]. Aside from *Rps8*, other R-genes that have been mapped to chr13 near Satt114 include *Rpa1*, *Rsv1*, *Rag2*, *rag4*, and *Rag5*. *Rpa1* confers race-specific resistance to *Pythium* damping-off caused by *Pythium aphanidermatum* in ‘Archer’ soybean, *Rsv1* confers strain-specific resistance to soybean mosaic virus, and *Rag2*, *rag4*, and *Rag5* confer biotype-specific resistance to soybean aphid (*Aphis glycines*) [46–49]. A fine-mapping of the *Rag2* gene from PI 200538 on chr13 showed that the *Rag2* gene(s) lies within a 54 kb interval containing seven genes one of which is a nucleotide-binding site-leucine-rich repeat gene. However, similar to other fine-mapping studies of disease resistance genes in soybean, this study did not further characterize all of the candidate genes within the fine-mapped region [37]. The fine-mapping study presented here is the first that fine-mapped as well as characterized the candidate genes within the fine-mapped interval containing the resistance genes. In five candidate genes annotated based on the Williams 82 DNA sequence from the 72.6 kb genomic region fine-mapped for the population FLS-594891, there were no prominent SNPs or mutations that may be linked to the FLS resistance in PI 594891. In addition, the expression level of the three aforementioned genes in PI 594891 was higher but not statistically different compared to that in Blackhawk. In this case, further study needs to be conducted to validate and pinpoint a true underlying gene that controls the resistance to FLS in PI 594891. As there were SNPs identified in Glyma13g25320, Glyma13g25340 (LRR), and Glyma13g25350 (transducin); and these three genes had higher expression levels in the two PIs compared to Blackhawk, a resistance mechanism via a combined action of multiple genes, as is the case at the *Rhg1* complex locus, is possible [50]. On the other hand, if only one gene is responsible for the resistance, Glyma13g25350 (transducin or Pleiotropic regulatory gene based on the prediction in the Phytozome.net) should be the first gene of interest. In PI 594774, this gene has a mutation that resulted in an amino acid change predicted to be deleterious for the protein function. PI 594774 and PI 594891 also had two common SNPs in the promoter region of this gene. Additionally, members of this class of the gene were reported to be highly expressed as a defense mechanism against attacking pathogens, such as in potato infected with *P*. *infestans* and in *Arabidopsis* challenged with *C*. *higginsianum* [51–55]. A pleiotropic regulatory gene (PLRG) was reported to be one of the three most important components of a protein complex that is essential for plant innate immunity [55]. In Arabidopsis, PLRG is a WD40 repeat protein shown to bind directly to an atypical R2R3 Myb transcription factor and together with a nuclear protein they make up the spliceosome-associated PRP Nineteen Complex (NTC) [55]. A mutant *plrg Arabidopsis* line had an increase in growth of *Pseudomonas syringae* pv *maculicola* up to more than 50 times compared to wild-type Col-0 control, indicating the role of this protein in the defense mechanism against plant pathogens [55]. The lack of deleterious SNPs in candidate genes in PI 594891 may hint at other possibilities such as the presence of a causal element residing outside of the sequenced regions of these five annotated genes or the effects of epigenetics. Nevertheless, a fine-mapped region of 72.6 kb would make it more feasible for future studies to enhance our knowledge about the mechanism of the resistance to *C*. *sojina* in soybean.

## Conclusions

This study has demonstrated a novel approach to fine-map genomic regions containing genes of interest using the SoySNP50K Infinium chip and KASP technology. Five annotated candidate genes were narrowed down, from which the SNPs that caused amino acid changes have been identified. Based on phenotype, genotype and haplotype analysis results, two soybean accessions might carry different resistance alleles of the same or different gene(s). The SNPs were used to develop KASP assays to detect the resistance alleles on chromosome 13 from the two PIs for marker-assisted selection in breeding programs.

## Supporting Information

S1 Table Primers used for KASP assays and qPCR (XLSX)

S2 Table DNA and protein alignment of Williams 82, Blackhawk, PI 594891, and PI 594774 for four candidate genes on chromosome 13. (DOCX)

S3 Table Results of SNP genotypes and reaction to *Cercospora sojina* Hara for PI 594774, PI 594891, Davis, Blackhawk, *and* 45 soybean lines and cultivars. (XLSX)

S4 Table Haplotype allele variation at the fine-mapped locus on chromosome 13 among PI 594891 and PI 594774, Davis, Blackhawk, and 45 soybean lines and cultivars. (XLSX)

S5 Table Haplotype allele variation at *Rcs3* locus on chromosome 16 among Davis, PI 594774, PI 594891, Blackhawk, and 45 soybean lines and cultivars. (XLSX)

## References

[1] Melchers LE. Diseases of cereal and forage crops in the United States in 1924. Plant Dis Rep Suppl. 1925; 40: 186.

[2] Akem CN, Dashiell KE. Effect of planting date on severity of frogeye leaf spot and grain yield of soybeans. Crop Protection. 1994; 13: 607–610.

[3] Sinclair JB, Backman PA. Compendium of soybean diseases, 3rd ed.: American Phytopathological Society, St Paul, MN; 1989.

[4] Ploper LD, González V, Gálvez MR, Devani MR, Ledesma F, Zamorano MA. Frogeye Leaf Spot of Soybean Caused by *Cercospora sojina* in Northwestern Argentina. Plant Disease. 2001; 85: 801.2–801.2. 10.1094/PDIS.2001.85.7.801B 30823211

[5] Carmona MA, Scandiani M and Luque A. Severe Outbreaks of Soybean Frogeye Leaf Spot Caused by *Cercospora sojina* in the Pampean Region, Argentina. Plant Disease. 2009; 93: 966–966. 30754559 10.1094/PDIS-93-9-0966B

[6] Koenning SR, Wrather JA. Suppression of soybean yield potential in the continental United States by plant diseases from 2006 to 2009. Plant Health Progress. 2010. doi: 101094/PHP-2010-1122-01-RS.

[7] Laviolette FA, Athow KL, Probst AH, Wilcox JR, Abney TS. Effect of Bacterial Pustule and Frogeye Leafspot on Yield of Clark Soybean1. Crop Sci. 1970; 10: 418–419.

[8] Mian MAR, Boerma HR, Phillips DV, Kenty MM, Shannon G, Shipe ER, et al Performance of Frogeye Leaf Spot-Resistant and-Susceptible Near-Isolines of Soybean. Plant Disease. 1998; 82: 1017–1021. 30856828 10.1094/PDIS.1998.82.9.1017

[9] Yorinori JT. Frogeye leaf spot of soybeans (*Cercospora sojina* Hara). in: World Soybean Production and Utilization Conference IV. R. Shibbles, ed. Westview Press Inc., Boulder, CO. 1987. pp. 1275–1283.

[10] Simonetti E, Carmona MA, Scandiani MM, García AF, Luque AG, Correa OS, et al Evaluation of indigenous bacterial strains for biocontrol of the frogeye leaf spot of soya bean caused by *Cercospora sojina* . Letters in Applied Microbiology. 2012; 55: 170–173. 10.1111/j.1472-765X.2012.03266.x 22671984

[11] Zhang G, Pedersen DK, Phillips DV, Bradley CA. Sensitivity of *Cercospora sojina* isolates to quinone outside inhibitor fungicides. Crop Protection. 2012; 40: 63–68.

[12] Zhang GR, Newman MA, Bradley CA. First Report of the Soybean Frogeye Leaf Spot Fungus (*Cercospora sojina*) Resistant to Quinone Outside Inhibitor Fungicides in North America. Plant Disease. 2012; 96: 767–767. 10.1016/j.mcna.2012.05.005 30727541

[13] Ma SM, Li BY. Primary report on the identification for physiological races of *Cercospora sojina* Hara in northeast China. Acta Phytopathol Sin. 1997; 27: 180.

[14] Yorinori JT. Management of foliar fungal diseases in Brazil In: Copping LG, et al (ed.) Pest management in soybean. Elsevier Applied Science, London; 1992 pp. 185–193

[15] Phillips DV, Boerma HR. *Cercospora sojina* race 5—a threat to soybeans in the southeastern United States. Phytopathology. 1981; 71: 334–336.

[16] Grau CR, Dorrance AE, Bond J, Russin JS. Fungal diseases In: Boerma H.R. and Specht J.E. (ed.) Soybeans: Improvement, production, and uses. 3rd ed. Agron. Monogr. 16 ASA, CSSA, and SSSA, Madison, WI 2004 pp. 679–763.

[17] Mian MAR, Missaoui AM, Walker DR, Phillips DV, Boerma HR. Frogeye Leaf Spot of Soybean: A Review and Proposed Race Designations for Isolates of *Cercospora sojina* Hara. Crop Sci. 2008; 48: 14–24.

[18] Athow K, Probst AH. The inheritance of resistance to frogeye leaf spot of soybeans. Phytopathology. 1952; 42: 660–662.

[19] Probst AH, Athow KL, Laviolette FA. Inheritance of resistance to race 2 of *Cercospora sojina* in soybeans. Crop Sci. 1965; 5: 332

[20] Phillips DV, Boerma HR. Two genes for resistance to race 5 of *Cercospora sojina* in soybeans. Phytopathology. 1982; 72: 764–766.

[21] Hoskin A. Genetic mapping of soybean resistance genes to frogeye leaf spot in five Chinese Plant Introductions and efficiency of early generation selection for low phytate soybean lines Institute of Plant Breeding, Genetics, and Genomics. University of Georgia 2011.

[22] Zou J, Dong W, Yang Q, Cao Y, Chen S. Inheritance of resistance to race 7 of *Cercospora sojina* in soybeans and RAPD tagging of the resistance gene. Chin Sci Bull. 1999; 44: 452–455.

[23] Song Q, Hyten DL, Jia G, Quigley CV, Fickus EW, Nelson RL, et al Development and evaluation of SoySNP50K, a high-density genotyping array for soybean. PLoS One. 2013; 8: e54985 10.1371/journal.pone.0054985 23372807 PMC3555945

[24] Fehr WR, Caviness CE, Burmood DT, Pennington JS. Stage of development descriptions for soybeans, Glycine max (L.) Merr. Crop Sci. 1971; 11: 929–931.

[25] Keim P, Olson T, Shoemaker R. A rapid protocol for isolating soybean DNA. Soybean Genetic Newsl. 1988; 15: 150–152.

[26] Diwan N, Cregan PB. Automated sizing of fluorescent-labeled simple sequence repeat (SSR) markers to assay genetic variation in soybean. Theor Appl Genet. 1997; 95: 723–733.

[27] Untergasser A, Nijveen H, Rao X, Bisseling T, Geurts R, Leunissen JA. Primer3Plus, an enhanced web interface to Primer3. Nucleic Acids Research. 2007; 35: W71–74. 17485472 10.1093/nar/gkm306 PMC1933133

[28] Libault M, Thibivilliers S, Bilgin DD, Radwan O, Benitez M, Clough SJ, et al Identification of Four Soybean Reference Genes for Gene Expression Normalization. Plant Genome. 2008; 1: 44–54.

[29] Livak KJ, Schmittgen TD. Analysis of Relative Gene Expression Data Using Real-Time Quantitative PCR and the 2−ΔΔCT Method. Methods. 2001; 25: 402–408. 11846609 10.1006/meth.2001.1262

[30] Missaoui AM, Phillips DV, Boerma HR. DNA marker analysis of ‘Davis’ soybean and its descendants for the *Rcs3* gene conferring resistance to *Cercospora sojina* . Crop Sci. 2007; 47: 1263–1270.

[31] Milne I, Shaw P, Stephen G, Bayer M, Cardle L, Thomas WT, et al Flapjack—graphical genotype visualization. Bioinformatics. 2010; 26: 3133–3134. 10.1093/bioinformatics/btq580 20956241 PMC2995120

[32] Kumar P, Henikoff S, Ng PC. Predicting the effects of coding non-synonymous variants on protein function using the SIFT algorithm. Nature Protocols. 2009; 4: 1073–1081. 10.1038/nprot.2009.86 19561590

[33] Crooks GE, Hon G, Chandonia J-M, Brenner SE.) WebLogo: A Sequence Logo Generator. Genome Research. 2004; 14: 1188–1190. 15173120 10.1101/gr.849004 PMC419797

[34] Yang Q, Zhang D, Xu M. A Sequential Quantitative Trait Locus Fine-Mapping Strategy Using Recombinant-Derived Progeny. Journal of Integrative Plant Biology. 2012; 54: 228–237. 10.1111/j.1744-7909.2012.01108.x 22348858

[35] Tao Y, Liu Q, Wang H, Zhang Y, Huang X, Wang B, et al Identification and fine-mapping of a QTL, qMrdd1, that confers recessive resistance to maize rough dwarf disease. BMC Plant Biology. 2013; 13: 145 10.1186/1471-2229-13-145 24079304 PMC3850639

[36] Wan X, Weng J, Zhai H, Wang J, Lei C, Liu X, et al Quantitative Trait Loci (QTL) Analysis For Rice Grain Width and Fine Mapping of an Identified QTL Allele gw-5 in a Recombination Hotspot Region on Chromosome 5. Genetics. 2008; 179: 2239–2252. 10.1534/genetics.108.089862 18689882 PMC2516094

[37] Kim K-S, Hill C, Hartman G, Hyten D, Hudson M, Diers BW. Fine mapping of the soybean aphid-resistance gene *Rag2* in soybean PI 200538. Theor Appl Genet. 2010; 121: 599–610. 10.1007/s00122-010-1333-6 20454773

[38] Yamashita Y, Takeuchi T, Ohnishi S, Sasaki J, Tazawa A. Fine mapping of the major Soybean dwarf virus resistance gene *Rsdv1* of the soybean cultivar ‘Wilis’. Breeding Science. 2013; 63: 417–422. 10.1270/jsbbs.63.417 24399914 PMC3859353

[39] Sun J, Li L, Zhao J, Huang J, Yan Q, Xing H, et al Genetic analysis and fine mapping of RpsJS, a novel resistance gene to Phytophthora sojae in soybean [*Glycine max* (L.) Merr.]. Theor Appl Genet. 2014; 127: 913–919. 10.1007/s00122-014-2266-2 24419901

[40] Song Q, Jia G, Zhu Y, Grant D, Nelson RT, Hwang EY, et al Abundance of SSR Motifs and Development of Candidate Polymorphic SSR Markers (BARCSOYSSR_1.0) in Soybean. Crop Sci. 2010; 50: 1950–1960.

[41] Burton JW, Brim CA, Young MF. Registration of ‘Young’ Soybean. Crop Sci. 1987; 27: 1093–1093.

[42] Boerma HR, Hussey RS, Phillips DV, Wood ED, Finnerty SL. Registration of ‘Cook’ Soybean. Crop Sci. 1992; 32: 497–497.

[43] Boerma HR, Hussey RS, Phillips DV, Wood ED, Finnerty SL. Registration of ‘Doles’ Soybean. Crop Sci. 1994; 34: 1411–1412.

[44] Carter TE, Burton JW, Cui Z, Zhou X, Villagarcia MR, Fountain MO, et al Registration of ‘N6201’ Soybean. Crop Sci. 2003; 43: 1125–a–1126.

[45] Gordon SG, St. Martin SK, Dorrance AE. Rps8 Maps to a Resistance Gene Rich Region on Soybean Molecular Linkage Group F. Crop Sci. 2006; 46: 168–173.

[46] Rosso ML, Rupe JC, Chen P. Mozzoni LA. Inheritance and Genetic Mapping of Resistance to Pythium Damping-Off Caused by Pythium aphanidermatum in ‘Archer’ Soybean. Crop Sci. 2008; 48: 2215–2222.

[47] Ashfield T, Danzer JR, Held D, Clayton K, Keim P, Saghai Maroof MA, et al *Rpg1*, a soybean gene effective against races of bacterial blight, maps to a cluster of previously identified disease resistance genes. Theor Appl Genet. 1998; 96: 1013–1021.

[48] Gore MA, Hayes AJ, Jeong SC, Yue YG, Buss GR, Saghai Maroof MA. Mapping tightly linked genes controlling potyvirus infection at the *Rsv1* and *Rpv1* region in soybean. Genome. 2002; 45: 592–599. 12033629 10.1139/g02-009

[49] Hill CB, Chirumamilla A, Hartman GL. Resistance and virulence in the soybean-Aphis glycines interaction. Euphytica. 2012; 186: 635–646.

[50] Cook DE, Lee TG, Guo X, Melito S, Wang K, Hughes TJ, et al Copy Number Variation of Multiple Genes at Rhg1 Mediates Nematode Resistance in Soybean. Science. 2012; 338: 1206–1209. 10.1126/science.1228746 23065905

[51] Narusaka Y, Narusaka M, Park P, Kubo Y, Hirayama T, Seki M, et al *RCH1*, a Locus in Arabidopsis That Confers Resistance to the Hemibiotrophic Fungal Pathogen Colletotrichum higginsianum. Molecular Plant-Microbe Interactions. 2004; 17: 749–762. 15242169 10.1094/MPMI.2004.17.7.749

[52] Tian ZD, Liu J, Wang BL, Xie CH. Screening and expression analysis of Phytophthora infestans induced genes in potato leaves with horizontal resistance. Plant Cell Rep. 2006; 25: 1094–1103. 16738852 10.1007/s00299-006-0169-7

[53] Wang H, Wijeratne A, Wijeratne S, Lee S, Taylor C, Martin SK, et al Dissection of two soybean QTL conferring partial resistance to Phytophthora sojae through sequence and gene expression analysis. BMC Genomics. 2012; 13: 428 10.1186/1471-2164-13-428 22925529 PMC3443417

[54] Weihmann T, Palma K, Nitta Y, Li X. Pleiotropic regulatory locus 2 exhibits unequal genetic redundancy with its homolog PRL1. Plant and Cell Physiology. 2012; 53: 1617–1626. 10.1093/pcp/pcs103 22813545

[55] Palma K, Zhao Q, Cheng YT, Bi D, Monaghan J, Cheng W, et al Regulation of plant innate immunity by three proteins in a complex conserved across the plant and animal kingdoms. Genes & Development. 2007; 21: 1484–1493. 17575050 10.1101/gad.1559607 PMC1891426

