# Supplementary material for: Fine Mapping and Characterization of Candidate Genes that Control Resistance to Cercospora sojina K. Hara in Two Soybean Germplasm Accessions
Source: PLoS One. 2015 May 19;10(5):e0126753. doi: 10.1371/journal.pone.0126753 (PMC4437980; doi:10.1371/journal.pone.0126753)
Supplement: S2 Table — (DOCX) [file pone.0126753.s002.docx]

**S2 Table.** DNA and protein alignment of Williams 82, Blackhawk, PI 594891, and PI 594774 for four candidate genes on chromosome 13.

ATCG: promoter

ATCG: 5’UTR

ATCG: exon/protein

ATCG: intron,

ATCG: 3’UTR

1a. Glyma13g25320_protein alignment

Blackhawk MVVGEQTNGWPPIGAPLNVHRDEHWTNFDSSVNAVSFGFVATAILISMFLVMAIFERYLR

Williams82 MVVGEQTNGWPPIGAPLNVHRDEHWTNFDSSVNAVSFGFVATAILISMFLVMAIFERYLR

PI594774 MVVGEQTNGWPPIGAPLNVHRDEHWTNFDSSVNAVSFGFVATAILISMFLVMAIFERYLR

PI594891 MVVGEQTNGWPPIGAPLNVHRDEHWTNFDSSVNAVSFGFVATAILISMFLVMAIFERYLR

Blackhawk PTSPPLSPSATTRRRSPSDVEAQIGFSGKLAHASPKMSVYASGVSVLMPGDEIPTFIAHP

Williams82 PTSPPLSPSATTRRRSPSDVEAQIGFSGKLAHASPKMSVYASGVSVLMPGDEIPTFIAHP

PI594774 PTSPPLSPSATTRRRSPSDVEAQIGFSGKLAHASPKMSVYASGVSVLMPGDEIPTFIAHP

PI594891 PTSPPLSPSATTRRRSPSDVEAQIGFSGKLAHASPKMSVYASGVSVLMPGDEIPTFIAHP

Blackhawk APCYPERISWPSHQHNNTLPCSSSNTLPTTTINQV

Williams82 APCYPERISWPSHQHNNTLPCSSSNTLPTTTINQV

PI594774 APCYPERISWPSHQHNNTLPCSSSNTLPTTTINQV

PI594891 APCYPERISWPSHQHNNTLPCSSSNTLPTATINQV

1b. Weblogo output of the amino acid conservation of Glyma13g25320 as part of the BLINK feature at NCBI using GI number 356548771. The top 100 best matched sequences were aligned and used as input for sequence LOGO <http://weblogo.berkeley.edu/logo.cgi> [website](http://www.webcitation.org/query.php?url=http://weblogo.berkeley.edu/logo.cgi&refdoi=10.1186/1471-2229-10-195). The logo consists of stacks of symbols, one stack for each position in the amino acid sequence. The overall height of the stack indicates the sequence conservation at that position, while the height of symbols within the stack indicates the relative frequency of each amino acid at that position. Red circle indicates residue changed due to the T^150^A mutation in PI 594891


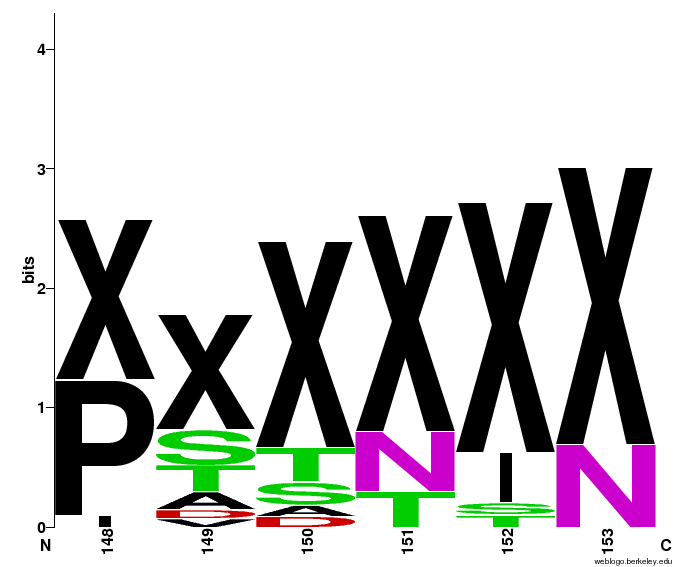


1c. Glyma13g25331_protein alignment

PI594891 MGCCVSSSHKTSTSSLAKFHDPPKPLTKPSESNSTVEETVKEVLLETPTTFAKSKPQKPH

Williams82 MGCCVSSSHKTSTSSLAKFHDPPKPLTKPSESNSTVEETVKEVLLETPTTFAKSKPQKPH

PI594774 MGCCVSSSHKTSTSSLAKFHDPPKPLTKPSESNSTVEETVKEVLLETPTTFAKSKPQKPN

Blackhawk MGCCVSSSHKTSTSSLAKFHDPPKPLTKPSESNSTVEETVKEVLLETPTTFAKSKPQKPN

PI594891 QNTNFHKFMEEKSKVEKRALTLSIYKAEDTTTSEEVCSLSRTVSRATSITDQREEMRKRV

Williams82 QNTNFHKFMEEKSKVEKRALTLSIYKAEDTTTSEEVCSLSRTVSRATSITDQREEMRKRV

PI594774 QNTNFHKFMEEKSKVEKRALTLSIYKAEDTTTSEEVCSLSRTVSRATSITDQREEMRKRV

Blackhawk QNTNFHKFMEEKSKVEKRALTLSIYKAEDTTTSEEVCSLSRTVSRATSITDQREEMRKRV

PI594891 YRSHPKLQKNRSFPGERRERTVVHGARNNNNVGSVRLVQCRDQTSQKIGNGGTGRRRDAA

Williams82 YRSHPKLQKNRSFPGERRERTVVHGARNNNNVGSVRLVQCRDQTSQKIGNGGTGRRRDAA

PI594774 YRSHPKLQKNRSFPGERRERTVVHGARNINNVGSVRLVQCRDQTSQKIGNGGTGRRRDAA

Blackhawk YRSHPKLQKNRSFPGERRERTVVHGARNINNVGSVRLVQCRDQTSQKIGNGGTGRRRDAA

PI594891 ENSLRRSRSQATVAGAGAARPVMGRSPYVRKPNRSTASIGIGTAENGGRKTEKPATTEKW

Williams82 ENSLRRSRSQATVAGAGAARPVMGRSPYVRKPNRSTASIGIGTAENGGRKTEKPATTEKW

PI594774 ENYLRRSRSQATVAGAGAARPVMGRSPYVRKPNRSTASIGTGTAENGGRKTEKPATTEKW

Blackhawk ENYLRRSRSQATVAGAGAARPVMGRSPYVRKPNRSTASIGTGTAENGGRKTEKPATTEKW

PI594891 PYAGESLENPLVSLECFIFI

Williams82 PYAGESLENPLVSLECFIFI

PI594774 PYAGESLENPLVSLECFIFI

Blackhawk PYAGESLENPLVSLECFIFI

1d. Glyma13g25340_DNA alignment

PI594891 TCAAGTTTTTGAATGTGTAATTGTCTTAAATAATAAAAAATAAAGTTTTGTTATTGATAA

PI594774 TCAAGTTTTTGAATGTGTAATTGTCTTAAATAATAAAAAATAAAGTTTTGTTATTGATAA

Williams82 TCAAGTTTTTGAATGTGTAATTGTCTTAAATAATAAAAAATAAAGTTTTGTTATTGATAA

Blackhawk TCAAGTTTTTGAATGTGTAATTGTCTTAAATAATAAAAAATAAAGTTTTGTTATTGATAA

PI594891 TGTTGTTATTTGATTCCAATAAAATTTCTTTTCGTGTGAAAAGATACATTTGATCTCTAA

PI594774 TGTTGTTATTTGATTCCAATAAAATTTCTTTTCGTGTGAAAAGATACATTTGATCTCTAA

Williams82 TGTTGTTATTTGATTCCAATAAAATTTCTTTTCGTGTGAAAAGATACATTTGATCTCTAA

Blackhawk TGTTGTTATTTGATTCCAATAAAATTTCTTTTCGTGTGAAAAGATACATTTGATCTCTAA

PI594891 CAAAACAATTCTAGGGGATAATAAG-TAAAACATTTTAATACCAAAGACAAGACACAATG

PI594774 CAAAACAATTCTAGGGGATAATAAGTAAAAACATTTTAATACCAAAGACAAGACACAATG

Williams82 CAAAACAATTCTAGGGGATAATAAG-TAAAACATTTTAATACCAAAGACAAGACACAATG

Blackhawk CAAAACAATTCTAGGGGATAATAAG-TAAAACATTTTAATACCAAAGACAAGACACAATG

PI594891 AGGAAAAGTTTAAATGCACACGAACTTGGATAGATTGAGCCAACAAATAACGGGACTCTT

PI594774 AGGAAAAGTTTAAATGCACACGAACTTGGATAGATTGAGCCAACAAATAACGGGACTCTT

Williams82 AGGAAAAGTTTAAATGCACACGAACTTGGATAGATTGAGCCAACAAATAATGGGACTCTT

Blackhawk AGGAAAAGTTTAAATGCACACGAACTTGGATAGATTGAGCCAACAAATAACGGGACTCTT

PI594891 TTCTAAATGGATTTGGCATGGGGTGGCAACTGTGTCGTTTTGTGCGTGATATTCGTCCCT

PI594774 TTCTAAATGGATTTGGCATGGGGTGGCAACTGTGTCGTTTTGTGCGTGATATTCGTCCCT

Williams82 TTCTAAATGGATTTGGCATGGGGTGGCAACTGTGTCGTTTTGTGCGTGATATTCGTCCCT

Blackhawk TTCTAAATGGATTTGGCATGGGGTGGCAACTGTGTCGTTTTGTGCGTGATATTCGTCCCT

PI594891 GCTCTCATGAAAGTGTACTTAAATATTGGAAAAGAGTGAATAAAACCTCCTGCGGAGGAT

PI594774 GCTCTCATGAAAGTGTACTTAAATATTAGAAAAGAGTGAATAAAACCTCCTGCGGAGGAT

Williams82 GCTCTCATGAAAGTGTACTTAAATATTGGAAAATAGTGAATAAAACCTCCTGCGGAGGAT

Blackhawk GCTCTCATGAAAGTGTACTTAAATATTGGAAAAGAGTGAATAAAACCTCCTGCGGAGGAT

PI594891 GATTACCATATGGTGCTTGTGACTGTGCCCCAATCTATGCCTTGCAAATTTCTATTAGGT

PI594774 GATTACCATATGGTGCTTGTGACTGTGCCCCAATCTATGCCTTGCAAATTTCTATTAGGT

Williams82 GATTACCATATGGTGCTTGTGACTGTGCCCCAATCTATGCCTTGCAAATTTCTATTAGGT

Blackhawk GATTACCATATGGTGCTTGTGACTGTGCCCCAATCTATGCCTTGCAAATTTCTATTAGGT

PI594891 GCGGCGAATGTTAAGGTATGAGATGTCCAATTTCAAGGTATAAGTTCAGTGATAATGGTT

PI594774 GCGGCGAATGTTAAGGTATGAGATGTCCAATTTTAAGGTATAAGTTCAGTGATAATGGTT

Williams82 GCGGCGAATGTTAAGGTATGAGATGTCCAATTTCAAGGTATAAGTTCAGTGATAATGGTT

Blackhawk GCGGCGAATGTTAAGGTATGAGATGTCCAATTTCAAGGTATAAGTTCAGTGATAATGGTT

PI594891 ATTGATTAATCTCAGAAACGTCCCGTGTGAGTTATGATGAGATGTACAATGATAATGAGA

PI594774 ATTGATTAATCTCAGAAACGTCCCGTGTGAGTTATGATGAGATGTACAATGATAATGAGA

Williams82 ATTGATTAATCTCAGAAACGTCCCGTGTGAGTTATGATGAGATGTACAATGATAATGAGA

Blackhawk ATTGATTAATCTCAGAAACGTCCCGTGTGAGTTATGATGAGATGTACAATGATAATGAGA

PI594891 GCCCATATACCTACAATGTTAATTAAACAAAGATTAGATTATAATAAATTAAAATAATAG

PI594774 GCCCATATACCTACAANGTTA-TTAAACAAAGATTGGATTATAATAAATTAAAATAATAG

Williams82 GCCCATATACCTACAATGTTAATTAAACAAAGATTAGATTATAATAAATTAAAATAATAG

Blackhawk GCCCATATACCTACAATGTTAATTAAACAAAGATTAGATTATAATAAATTAAAATAATAG

PI594891 ACAATTATAAAACTAATTATTTTAATACTATCATGAATTCTTTTTTCTTTGAACATGAGG

PI594774 ACAATTATAAAACTAATTATTTTAATACTATCATGAATTCTTTTTTCTTTGAACATGAGG

Williams82 ACAATTATAAAACTAATTATTTTAATACTATCATGAATTCTTTTTTCTTTGAACATGAGG

Blackhawk ACAATTATAAAACTAATTATTTTAATACTATCATGAATTCTTTTTTCTTTGAACATGAGG

PI594891 TGTCTCTCCTCATGGAACTAAAGACTAGTCCTTAGTATTGTCATCTATGCAACAAGTAGA

PI594774 TGTCTCTCCTCATGGAACTAAAGACTAGTCCTTAGTATTGTCATCTATGCAACAAGTAGA

Williams82 TGTCTCTCCTCATGGAACTAAAGACTAGTCCTTAGTATTGTCATCTATGCAACAAGTAGA

Blackhawk TGTCTCTCCTCATGGAACTAAAGACTAGTCCTTAGTATTGTCATCTATGCAACAAGTAGA

PI594891 AAAAGAACAATGAATTTTCTGATAATTCGAATTGAAAATGATAAAAAAGAGAGAATGA

PI594774 AAAAGAACAATGAATTTTCTGATAATTCGAATTGAAAATGATAAAAAAGAGAGAATGA

Williams82 AAAAGAACAATGAATTTTCTGATAATTCGAATTGAAAATGATAAAAAAGAGAGAATGA

Blackhawk AAAAGAACAATGAATTTTCTGATAATTCGAATTGAAAATGATAAAAAAGAGAGAATGA

PI594891 AGAAGATTGTGTTAGTTTAGATTATTTTATTTTATTTTGAAATAGTTTAATTATAATTAA

PI594774 AGAAGATTGTGTTAGTTTAGATTATTTTATTTTATTTTGAAATAGTTTAATTATAATTAA

Williams82 AGAAGATTGTGTTAGTTTAGATTATTTTATTTTATTTTGAAATAGTTTAATTATAATTAA

Blackhawk AGAAGATTGTGTTAGTTTAGATTATTTTATTTTATTTTGAAATAGTTTAATTATAATTAA

PI594891 ACCCTTATGGGGGAAACTCCTAAATGTTCAATTACTTGTTTCAAGAAAATTATGGATCTC

PI594774 ACCCTTATGGGGGAAACTCCTAAATGTTCAATTACTTGTTTCAAGAAAATTATGGATCTC

Williams82 ACCCTTATGGGGGAAACTCCTAAATGTTCAATTACTTGTTTCAAGAAAATTATGGATCTC

Blackhawk ACCCTTATGGGGGAAACTCCTAAATGTTCAATTACTTGTTTCAAGAAAATTATGGATCTC

PI594891 TTACGACAGATATTTATGAAAAAAAATATTAAAAAAGTGTTTTTTTTCTTATAGTTTCAT

PI594774 TTACGACAGATATTTATGAAAAAAAATATTAAAAAAGTGTTTTTTTTCTTATAGTTTCAT

Williams82 TTACGACAGATATTTATGAAAAAAAATATTAAAAAAGTGTTTTTTTTCTTATAGTTTCAT

Blackhawk TTACGACAGATATTTATGAAAAAAAATATTTAAAAAGTGTTTTTTTTCTTATAGTTTCAT

PI594891 TAACATCAATCTAAATGACTAATTTAAATAATTAAAAGACAAGATTTTATTGAAGAATAT

PI594774 TAACATCAATCTAAATGACTAATTTAAATAATTAAAAGACAAGATTTTATTGAAGAATAT

Williams82 TAACATCAATCTAAATGACTAATTTAAATAATTAAAAGACAAGATTTTATTGAAGAATAT

Blackhawk TAACATCAATCTAAATGACTAATTTAAATAATTAAAAGACAAGATTTTATTGAAGAATAT

PI594891 ATTAAAAATAAAATAAAAAAGTGTAATAAATGTTTTTCCTAGTAAAATATTATCAATTT

PI594774 ATTAAAAATAAAATAAAAAAGTGTAATAAATGTTTTTCCTAGTAAAATATTATCAATTT

Williams82 ATTAAAAATAAAATAAAAAAGTGTAATAAATGTTTTTCCTAGTAAAATATTATCAATTT

Blackhawk ATTAAAAATAAAATAAAAAAGTGTAATAAATGTTTTTCCTAGTAAAATATTATCAATTT

PI594891 TAGTTGGTTAATTAAAGCCATCCTTTAATTTTGATGTTAATATTTTTCTTTTTCTTAATA

PI594774 TAGTTGGTTAATTAAAGCCATCCTTTAATTTTGATGTTAATATTTTTCTTTTTCTTAATA

Williams82 TAGTTGGTTAATTAAAGCCATCCTTTAATTTTGATGTTAATATTTTTCTTTTTCTTAATA

Blackhawk TAGTTGGTTAATTAAAGCCATCCTTTAATTTTGATGTTAATATTTTTCTTTTTCTTAATA

PI594891 TAATTAACATTAAACATGGTCATGAGTTCCTGATGTGTGAAATTCTATATGCTATTAGA

PI594774 TAATTAACATTAAACATGGTCATGAGTTCCTGATGTGTGAAATTCTATATGCTATTAGA

Williams82 TAATTAACATTAAACATGGTCATGAGTTCCTGATGTGTGAAATTCTATATGCTATTAGA

Blackhawk TAATTAACATTAAACATGGTCATGAGTTCCTGATGTGTGAAATTCTATATGCTATTAGA

PI594891 AGTATCACGATAAACACTTCTAAGTTCTAATCAGAAAACCTCGTCATTCGTCACTGATTC

PI594774 AGTATCACGATAAACACTTCTAAGTTCTAATCAGAAAACCTCGTCATTCGTCACTGATTC

Williams82 AGTATCACGATAAACACTTCTAAGTTCTAATCAGAAAACCTCGTCATTCGTCACTGATTC

Blackhawk AGTATCACGATAAACACTTCTAAGTTCTAATCAGAAAACCTCGTCATTCGTCACTGATTC

PI594891 AATGCATTGGATTCTTGGAGGAACAAAAGAACAAGACACACTTTGCATGTGTTTTGCTT

PI594774 AATGCATTGGATTCTTGGAGGAACAAAAGAACAAGACACACTTTGCATGTGTTTTGCTT

Williams82 AATGCATTGGATTCTTGGAGGAACAAAAGAACAAGACACACTTTGCATGTGTTTTGCTT

Blackhawk AATGCATTGGATTCTTGGAGGAACAAAAGAACAAGACACACTTTGCATGTGTTTTGCTT

PI594891 GTAGTAACAAGGAATTTCTTACCTGCCAACGAAGCATTGAAATCTAGGAGGCCAATGAAA

PI594774 GTAGTAACAAGGAATTTCTTACCTGCCAACGAAGCATTGAAATCTAGGAGGCCAATGAAA

Williams82 GTAGTAACAAGGAATTTCTTACCTGCCAACGAAGCATTGAAATCTAGGAGGCCAATGAAA

Blackhawk GTAGTAACAAGGAATTTCTTACCTGCCAACGAAGCATTGAAATCTAGGAGGCCAATGAAA

PI594891 GTGCACAAACACGCAATAAAAAGAAGAAAAATGAGTAATACTTTTTTGTCAGAGTTTTCG

PI594774 GTGCACAAACACGCAATAAAAAGAAGAAAAATGAGTAATATTTTTT-GTCAGAGTTTTCG

Williams82 GTGCACAAACACGCAATAAAAAGAAGAAAAATGAGTAATATTTTTT-GTCAGAGTTTTCG

Blackhawk GTGCACAAACACGCAATAAAAAGAAGAAAAATGAGTAATACTTTTTTGTCAGAGTTTTCG

PI594891 ATTGGTCATATGTATAACCATAAATCTAGATTAGATTTTGCAATTGGTAAGTGACTCAGC

PI594774 ATTGGTCATATGTATAACCATAAATCTAGATTAGATTTTGCAATTGGTAAGTGACTCAGC

Williams82 ATTGGTCATATGTATAACCATAAATCTAGATTAGATTTTGCAATTGGTAAGTGACTCAGC

Blackhawk ATTGGTCATATGTATAACCATAAATCTAGATTAGATTTTGCAATTGGTAAGTGACTCAGC

PI594891 CTGCATCAGACGGATGATATTTAAGTAAAATAAATAAATTGTACGTGATTCTTTCTTTTT

PI594774 CTGCATCAGACGGATGATATTTAAGTAAAATAAATAAATTGTACGTGATTCTTTCTTTTT

Williams82 CTGCATCAGACGGATGATATTTAAGTAAAATAAATAAATTGTACGTGATTCTTTCTTTTT

Blackhawk CTGCATCAGACGGATGATATTTAAGTAAAATAAATAAATTGTACGTGATTCTTTCTTTTT

PI594891 TATTTCACTATCCTAGTATCCTCTGTCTTAGACAAACATAAAATTTAAGAACCTGTGGAA

PI594774 TATTTCACTATCCTAGTATCCTCTGTCTTAGACAAACATAAAATTTAAGAACCTGTGGAA

Williams82 TATTTCACTATCCTAGTATCCTCTGTCTTAGACAAACATAAAATTTAAGAACCTGTGGAA

Blackhawk TATTTCACTATCCTAGTATCCTCTGTCTTAGACAAACATAAAATTTAAGAACCTGTGGAA

PI594891 TCAACATAAATAAATCATATTTCAATCAACAGCAAACACACCGCAAAGGTGAGTGCTAAA

PI594774 TCAACATAAATAAATCATATTTCAATCAACAGCAAACACACCGCAAAGGTGAGTGCTAAA

Williams82 TCAACATAAATAAATCATATTTCAATCAACAGCAAACACACCGCAAAGGTGAGTGCTAAA

Blackhawk TCAACATAAATAAATCATATTTCAATCAACAGCAAACACGCCGCAAAGGTGAGTGCTAAA

PI594891 TCCTCTGTATTAGAATCGTTATTTTTTATCACCAAGTACATTAATCAATTGACATCAGAT

PI594774 TCCTCTGTATTAGAATCGTTATTTTTTATCACCAAGTACATTAATCAATTGACATCAGAT

Williams82 TCCTCTGTATTAGAATCGTTATTTTTTATCACCAAGTACATTAATCAATTGACATCAGAT

Blackhawk TCCTCTGTATTAGAATCGTT-TTTTTTATCACCAAGTACATTAATCAATTGACATCAGAT

PI594891 TATTCTATTTTAACTACAAGTTTTGACTTCAATTTCTCACTTATTTTCTTAGAAGTGACT

PI594774 TATTCTATTTTAACTACAAGTTTTGACTTCAATTTCTCACTTATTTTCTTAGAAGTGACT

Williams82 TATTCTATTTTAACTACAAGTTTTGACTTCAATTTCTCACTTATTTTCTTAGAAGTGACT

Blackhawk TATTCTATTTTAACTACAAGTTTTGACTTCAATTTCTCACTTATTTTCTTAGAAGTGACT

PI594891 GCTACTCATCAAGCTCAAGGAAACAATTAACTACCTCTTCAGATCTGAGGATGACATGGA

PI594774 GCTACTCATCAAGCTCAAGGAAACAATTAACTACCTCTTCAGATCTGAGGATGACATGGA

Williams82 GCTACTCATCAAGCTCAAGGAAACAATTAACTACCTCTTCAGATCTGAGGATGACATGGA

Blackhawk GCTACTCATCAAGCTCAAGGAAACAATTAACTACCTCTTCAGATCTGAGGATGACATGGA

PI594891 CCAGACTAATAATTCATCTTTTTCTTCTATACTCATTATGTTTATGAAAACAACAACGGC

PI594774 CCAGACTAATAATTCATCTTTTTCTTCTATACTCATTATGTTTATGAAAACAACAACGGC

Williams82 CCAGACTAATAATTCATCTTTTTCTTCTATACTCATTATGTTTATGAAAACAACAACGGC

Blackhawk CCAGACTAATAATTCATCTTTTTCTTCTATACTCATTATGTTTATGAAAACAACAACGGC

PI594891 ATCGGAGCTGGAAAGGACATAGCCTAAAAAAGGAAAAAAGGTTTTAAAAATAAACGAAAA

PI594774 ATCGGAGCTGGAAAGGACATAGCCTAAAAAAGGAAAAAAGGTTTTAAAAATAAACGAAAA

Williams82 ATCGGAGCTGGAAAGGACATAGCCTAAAAAAGGAAAAAAGGTTTTAAAAATAAACGAAAA

Blackhawk ATCGGAGCTGGAAAGGACATAGCCTAAAAAAGGAAAAAAGGTTTTAAAAATAAACGAAAA

PI594891 AACAAATTTCATATTCTCATACGAAGTTATTACTTGGGATATTTTCTTTTTTATTGATTC

PI594774 AACAAATTTCATATTCTCATACGAAGTTATTACTTGGGATATTTTCTTTTTTATTGATTC

Williams82 AACAAATTTCATATTCTCATACGAAGTTATTACTTGGGATATTTTCTTTTTTATTGATTC

Blackhawk AACAAATTTCATATTCTCATACGAAGTTATTACTTGGGATATTTTCTTTTTTATTGATTC

PI594891 GGTCTTTGAGTAATGAAGAATCAACTTTCAATCTGAAAATTATCATTCAAAAGAGAGTTT

PI594774 GGTCTTTGAGTAATGAAGAATCAACTTTCAATCTGAAAATTATCATTCAAAAGAGAGTTT

Williams82 GGTCTTTGAGTAATGAAGAATCAACTTTCAATCTGAAAATTATCATTCAAAAGAGAGTTT

Blackhawk GGTCTTTGAGTAATGAAGAATCAACTTTCAATCTGAAAATTATCATTCAAAAGAGAGTTT

PI594891 GGCTGATTTATAAACTTCATGTCCAAAATATTCATCAAAGCTGATTTGTTGGGCATAAAT

PI594774 GGCTGATTTATAAACTTCATGTCCAAAATATTCATCAAAGCTGATTTGTTGGGCATAAAT

Williams82 GGCTGATTTATAAACTTCATGTCCAAAATATTCATCAAAGCTGATTTGTTGGGCATAAAT

Blackhawk GGCTGATTTATAAACTTCATGTCCAAAATATTCATCAAAGCTGATTTGTTGGGCATAAAT

PI594891 TTCTAAGGGCACAAAGATTCAAATTAGAACGGATTAAGTAAAAAAGAAAAGAAAATTAAC

PI594774 TTCTAAGGGCACAAAGATTCAAATTAGAACGGATTAAGTAAAAAAGAAAAGAAAATTAAC

Williams82 TTCTAAGGGCTCAAAGATTCAAATTAGAACGGATTAAGTAAAAAAGAAAAGAAAATTAAC

Blackhawk TTCTAAGGGCACAAAGATTCAAATTAGAACGGATTAAGTAAAAAAGAAAAGAAAATTAAC

PI594891 CTGATATGAATTCCGAAATTAATGAGAGTTTTCCAATACCACCATAAAAATAAACACGTA

PI594774 CTGATATGAATTCCGAAATTAATGAGAGTTTTCCAATACCACCATAAAAATAAACACGTA

Williams82 CTGATATGAATTCCGAAATTAATGAGAGTTTTCCAATACCACCATAAAAATAAACACGTA

Blackhawk CTGATATGAATTCCGAAATTAATGAGAGTTTTCCAATACCACCATAAAAATAAACACGTA

PI594891 AGGGTTACACTTGATTTAAAAACTATCTCATGTCTTAAAAATGGGTGAAACTTATTGGGC

PI594774 AGGGTTACACTTGATTTAAAAACTATCTCATGTCTTAAAAATGGGTGAAACTTATTGGGC

Williams82 AGGGTTACACTTGATTTAAAAACTATCTCATGTCTTAAAAATGGGTGAAACTTATTGGGC

Blackhawk AGGGTTACACTTGATTTAAAAACTATCTCATGTCTTAAAAATGGGTGAAACTTATTGGGC

PI594891 TCAAAATTTAAAACCATGCAATGAATCTATGACTCTTTYCACTCATAAAGCCCTTGCTTA

PI594774 TCAAAATTTAAAACCATGCAATGAATCTATGACTCTTTTCACTCATAAAGCCCTTGCTTA

Williams82 TCAAAATTTAAAACCATGCAATGAATCTATGACTCTTTTCACTCATAAAGCCCTTGCTTA

Blackhawk TCAAAATTTAAAACCATGCAATGAATCTATGACTCTTTTCACTCATAAAGCCCTTGCTTA

PI594891 ACCTATTTCTTACTACCCTGAGAGACTTGGCCACCAGTGAATTTGAAACATTAGATTTCT

PI594774 ACCTATTTCTTACTACCCTGAGAGACTTGACCACCAGTGAATTTGAAACATTAGATTTCT

Williams82 ACCTATTTCTTACTACCCTGAGAGACTTGACCACCAGTGAATTTGAAACATTAGATTTCT

Blackhawk ACCTATTTCTTACTACCCTGAGAGACTTGGCCACCAGTGAATTTGAAACATTAGATTTCT

PI594891 GGGTGTTATTTAGAACCTCTGATTTTGTTAAGGTTTTTCCTTTGAAGTAAAAAAAAAAAC

PI594774 GGGTGTTATTTAGAACCTCTGATTTTGTTAAGGTTTTTCCTTTGAAGTAAAAAAAAAAAC

Williams82 GGGTGTTATTTAGAACCTCTGATTTTGTTAAGGTTTTTCCTTTGAAGTAAAAAAAAAAAC

Blackhawk GGGTGTTATTTAGAACCTCTGATTTTGTTAAGGTTTTTCCTTTGAAGTAAAAAAAAAAAC

PI594891 TTTTTAAAACCCACATGCTCGTGCCTGTAACAACGATTAGATGACATACTTGTTGCAGAT

PI594774 TTTTTAAAACCCACATGCTCGTGCCTGTAACAACGATTAGATGACATACTTGTTGCAGAT

Williams82 TTTTTAAAACCCACATGCTCGTGCCTGTAACAACGATTAGATGACATACTTGTTGCAGAT

Blackhawk TTTTTAAAACCCACATGCTCGTGCCTGTAACAACGATTAGATGACATACTTGTTGCAGAT

PI594891 TGGAATAAAATTTAGCATGCTCTTCAATCAAAAACCAATTTGTGTTGCACCTCCTTTTCA

PI594774 TGGAATAAAATTTAGCATGCTCTTCAATCAAAAACCAATTTGTGTTGCACCTCCTTTTCA

Williams82 TGGAATAAAATTTAGCATGCTCTTCAATCAAAAACCAATTTGTGTTGCACCTCCTTTTCA

Blackhawk TGGAATAAAATTTAGCATGCTCTTCAATCAAAAACCAATTTGTGTTGCACCTCCTTTTCA

PI594891 TAACAAAGAATAGAAATGGGCCAACTAAGAGACTAGTAGTATCACATCCGCAAGCAGATA

PI594774 TAACAAAGAATAGAAATGGGCCAACTAAGAGACTAGTAGTATCACATCCGCAAGCAGATA

Williams82 TAACAAAGAATAGAAATGGGCCAACTAAGAGACTAGTAGTATCACATCCGCAAGCAGATA

Blackhawk TAACAAAGAATAGAAATGGGCCAACTAAGAGACTAGTAGTATCACATCCGCAAGCAGATA

PI594891 CCAAAGTCCAATTTCAATTTGACACAAGAGTGTTGCTAGTTGCTGGGTGCACCTAACAT

PI594774 CCAAAGTCCAATTTCAATTTGACACAAGAGTGTTGCTAGTTGCTGGGTGCACCTAACAT

Williams82 CCAAAGTCCAATTTCAATTTGACACAAGAGTGTTGCTAGTTGCTGGGTGCACCTAACAT

Blackhawk CCAAAGTCCAATTTCAATTTGACACAAGAGTGTTGCTAGTTGCTGGGTGCACCTAACAT

PI594891 TTAATTAAAATACCATTTAAACAAAAAAAAATTGATGGGATTTGAGGAAAAAAAAC

PI594774 TTAATTAAAATACCATTTAAACAAAAAAAATTGATGGG-ATTTGAGGAAAAAAAAC

Williams82 TTAATTAAAATACCATTTAAACAAAAAAAATTGATGGG-ATTTGAGGAAAAAAAAC

Blackhawk TTAATTAAAATACCATTTAAACAAAAAAAAATTGATGGGATTTGAGGAAAAAAAAC

PI594891 TACAAAAAATAAATATTTAAAAGATGAGTGATAACTCTCTAAAGTTATATATATATAT--

PI594774 TACAAAAAATAAATATTTAAAAGATGAGTGATAACTCTCTAAAGTTATATATATATAT--

Williams82 TACAAAAAATAAATATTTAAAAGATGAGTGATAACTCTCTAAAGTTATATATATATAT--

Blackhawk TACAAAAAATAAATATTTAAAAGATGAGTGATAACTCTCTAAAGTTATATATATATATAT

PI594891 ACATGCTTCTTTATTAGTTTCGAAATTCAAAGGAGAATATATAAAGCTATTGAAATTCAG

PI594774 ACATGCTTCTTTATTAGTTTCGAAATTCAAAGGAGAATATATAAAGCTATTGAAATTCAG

Williams82 ACATGCTTCTTTATTAGTTTCGAAATTCAAAGGAGAATATATAAAGCTATTGAAATTCAG

Blackhawk ACATGCTTCTTTATTAGTTTCGAAATTCAAAGGAGAATATATAAAGCTATTGAAATTCAG

PI594891 GGACCATTTAAGATGAATAATAAATCCAAAACGTTAAGTATACAATATTAATTTATTTTT

PI594774 GGACCATTTAAGATGAATAATAAATCCAAAACGTTAAGTATACAATATTAATTTATTTTT

Williams82 GGACCATTTAAGATGAATAATAAATCCAAAACGTTAAGTATACAATATTAATTTATTTTT

Blackhawk GGACCATTTAAGATGAATAATAAATCCAAAACGTTAAGTATACAATATTAATTTATTTTT

PI594891 TTCTTTTTGTAAAGTAATAATACAATTTTTTTAAATCTTCTTATTATAATCCCCTTATCA

PI594774 TTCTTTTTGTAAAGTAATAATACAATTTTTTTAAATCTTCTTATTATAATCCCCTTATCA

Williams82 TTCTTTTTGAAAAGTAATAATACAATTTTTTTAAATCTTCTTATTATAATCCCCTTATCA

Blackhawk TTCTTTTTGTAAAGTAATAATACAATTTTTTTAAATCTTCTTATTATAATCCCCTTATCA

PI594891 AATAAAAAAAAA-TCAAAGGACAAGAACGTTTTTAAACGAAAACAATCATCTATAGAAAG

PI594774 AATAAAAAAAAAATCAAAGGACAAGAACGTTTTTAAACGAAAACAATCATCTATAGAAAG

Williams82 AATAAAAAAAAAATCAAAGGACAAGAACGTTTTTAAACGAAAACAATCATCTATAGAAAG

Blackhawk AATAAAAAAAAA-TCAAAGGACAAGAACGTTTTTAAACGAAAACAATCATCTATAGAAAG

PI594891 CAATCCTTTACAACAAAGGTGAAAATTTCTAAACTAAAGGAACATAAAACAAAATTGAAA

PI594774 CAATCCTTTACAACAAAGGTGAAAATTTCTAAACTAAAGGAACATAAAACAAAATTGAAA

Williams82 CAATCCTTTACAACAAAGGTGAAAATTTCTAAACTAAAGGAACATAAAACAAAATTGAAA

Blackhawk CAATCCTTTACAACAAAGGTGAAAATTTCTAAACTAAAGGAACATAAAACAAAATTGAAA

PI594891 ATATTAATACAAGATAAAAAAAATCTTTTGTATGAGTTGTAAACAGTGTTTGTTGTCCAT

PI594774 ATATTAATACAAGATAAAAAAAATCTTTTGTATGAGTTGTAAACAGTGTTTGTTGTCCAT

Williams82 ATATTAATACAAGATAAAAAAAATCTTTTGTATGAGTTGTAAACAGTGTTTGTTGTCCAT

Blackhawk ATATTAATACAAGATAAAAAAAATCTTTTGTATGAGTTGTAAACAGTGTTTGTTGTCCAT

PI594891 TGTCCATGTAGTTTTTGAAGAGTAGGTTGCTAACCGCTAAGCAAAAAAGATTCAACAAGG

PI594774 TGTCCATGTAGTTTTTGAAGAGTAGGTTGCTAACCGCTAAGCAAAAAAGATTCAACAAGG

Williams82 TGTCCATGTAGTTTTTGAAGAGTAGGTTGCTAACCGCTAAGCAAAAAAGATTCAACAAGG

Blackhawk TGTCCATGTAGTTTTTGAAGAGTAGGTTGCTAACCGCTAAGCAAAAAAGATTCAACAAGG

PI594891 CAGACATGAAAAGGAAAGAGAAAGATGAAAAGAGAAGAATACAACGATTGGATAA-TATT

PI594774 CAGACATGAAAAGGAAAGAGAAAGATGAAAAGAGAAGAATACAACGATTGGATAAATATT

Williams82 CAGACATGAAAAGGAAAGAGAAAGATGAAAAGAGAAGAATACAACGATTGGATAAATATT

Blackhawk CAGACATGAAAAGGAAAGAGAAAGATGAAAAGAGAAGAATACAACGATTGGATAA-TATT

PI594891 AATCCAAATTTGAAAGAAAACATTAATTACAAATTTAAAATTTCAACAAAAGGGAAATTT

PI594774 AATCCAAATTTGAAAGAAAACATTAATTACAAATTTAAAATTTCAACAAAAGGGAAATTT

Williams82 AATCCAAATTTGAAAGAAAACATTAATTACAAATTTAAAATTTCAACAAAAGGGAAATTT

Blackhawk AATCCAAATTTGAAAGAAAACATTAATTACAAATTTAAAATTTCAACAAAAGGGAAATTT

PI594891 TATAAATTAAGTAACTTATGCAGAAACAATAATTATAATTATTAAATAGTGCAAGATTTT

PI594774 TATAAATTAAGTAACTTATGCAGAAACAATAATTATAATTATTAAATAGTGCAAGATTTT

Williams82 TATAAATTAAGTAACTTATGCAGAAACAATAATTATAATTATTAAATAGTGCAAGATTTT

Blackhawk TATAAATTAAGTAACTTATGCAGAAACAATAATTATAATTATTAAATAGTGCAAGGTTTT

PI594891 TTTAAGGAATAGTGCAATG-----------------------CTATCAACGTAACTTTCT

PI594774 TTTAAGGAATAGTGCAATG-----------------------CTATCAACGTAACTTTCT

Williams82 TTTAAGGAATAGTGCAAGATTTTTTTAAGGAATAGTGCAATGCTATCAACGTAACTTTCT

Blackhawk TTTAAGGAATAGTGCAATG-----------------------CTATCAACGTAACTTTCT

PI594891 TATTTTCAATAGCATTTTTATATTGCAAAATCAATATCAAATTAAAAATAACCTTTTTAA

PI594774 TATTTTCAATAGCATTTTTATATTGCAAAATCAATATCAAATTAAAAATAACCTTTTTAA

Williams82 TATTTTCAATAGCATTTTTATATTGCAAAATCAATATCAAATTAAAAATAACCTTTTTAA

Blackhawk TATTTTCAATAGCATTTTTATATTGCAAAATCAATATCAAATTAAAAATAACCTTTTTAA

PI594891 CTCTAATTTGTACGGAAAATCCTTAAAATAAACTCAACACTTCTATTTGTGAGTATATAT

PI594774 CTCTAATTTGTACGGAAAATCCTTAAAATAAACTCAACACAAATAT--------ATATAT

Williams82 CTCTAATTTGTACGGAAAATCCTTAAAATAAACTCAACACTTCTATTTGTGAGTATATAT

Blackhawk CTATAATTTGTACGGAAAATCCTTAAAATAAACTCAACACAAATAT--------ATATAT

PI594891 TTTTATTTTTTACCATCACATTTATGTGAATTATTTGCCAAATTTTTTTAGGAGAAATTA

PI594774 TTTTATTTTTTACCATCACATTTATGTGAATTATTTGCCAAATTTTTTTAGGAGAAATTA

Williams82 TTTTATTTTTTACCATCACATTTATGTGAATTATTTGCCAAATTTTTTTAGGAGAAATTA

Blackhawk TTTTATTTTTTACCATCACATTTATGTGAATTATTTGCCAAATTTTTTTAGGAGAAATTA

PI594891 TCTACCAATTTTGTTATCACTAATTTTACAGCAAATCTAAATAATCATATTTAGTCGAGT

PI594774 TCTACCAATTTTGTTATCACTAATTTTACAGCAAATCTAAATAATCATATTTAGTCGAGT

Williams82 TCTACCAATTTTGTTATCACTAATTTTACAGCAAATCTAAATAATCATATTTAGTCGAGT

Blackhawk TCTACCAATTTTGTTATCACTAATTTTACAGCAAATCTAAATAATCATATTTAGTCGAGT

PI594891 CTTTCATTTTGACCATTTTTTTCTTCTATCTATCAAGATTTTCCTTAAAGAATCTTAGTC

PI594774 CTTTCATTTTGACCATTTTTTTCTTCTATCTATCAAGATTTTCCTTAAAGAATCTTAGTC

Williams82 CTTTCATTTTGACCATTTTTTTCTTCTATCTATCAAGATTTTCCTTAAAGAATCTTAGTC

Blackhawk CTTTCATTTTGACCATTTTTTTCTTCTATCTATCAAGATTTTCCTTAAAGAATCTTAGTC

PI594891 TAATTTTACTGTTGATTGTATAATTCATTTTAGAATTATGTGTGATTATTTTTATGATAA

PI594774 TAATTTTACTGTTGATTGTATAATTCATTTTAGAATTATGTGTGATTATTTTTATGATAA

Williams82 TAATTTTACTGTTGATTGTATAATTCATTTTAGAATTATGTGTGATTATTTTTATGATAA

Blackhawk TAATTTTACTGTTGATTGTATAATTCATTTTAGAATTATGTGTGATTATTTTTATGATAA

PI594891 AATTTTCACAACAAAGATGTAACAAAGTTTGATACAATTTTTGTCCTG-ATTATAAATAA

PI594774 AATTTTCACAACAAAGATATAACAAAGTTTGATACAATTTTTGTCCCTGATTATAAATAA

Williams82 AATTTTCACAACAAAGATATAACAAAGTTTGATACAATTTTTGTCCTG-ATTATAAATAA

Blackhawk AATTTTCTCAACAAAGATATAACAAAGTTTGATACAATTTTTGTCCCTGATTATAAATAA

PI594891 TATAATTGATTAGAACAATCAGTTTTTTTTGGGGGGTTCAACTACCGCTTTGGTTTACGG

PI594774 TATAATTGATTAGAACAATCAGTTTTTTTTTTGGGGTTCAACTACCGCTTTGGTTTACGG

Williams82 TATAATTGATTAGAACAATCAGTTTTTTTTGGGGGGTTCAACTACCGCTTTGGTTTACGG

Blackhawk TATAATTGATTAGAACAATCAGTTTTTTTTTTGGGGTTCAACTACCGCTTTGGTTTACGG

PI594891 GTGGGAGCCTCAGTTATCTGCACAAATTAATTTATGATTTTTTTTTAAAGTGTATTATAC

PI594774 GTGGGAGCCTCAGTTATCTGCACAAATTAATTTTTGATTTTTTTT--AAGTGTATTATGC

Williams82 GTGGGAGCCTCAGTTATCTGCACAAATTAATTTATGATTTTTTTTTAAAGTGTATTATAC

Blackhawk GTGGGAGCCTCAGTTATCTGCACAAATTAATTTTTGATTTTTTTT---AGTGTATTATGC

PI594891 AAGAATTAAATATAATTTTTTTCCCACAAATTTAGCGTATGTTGTCTAGCTATACTAAAA

PI594774 AAGAATTAAATATAATTTTTT-CCCACAAATTTAGCCTATGTTGTCTAGCTATACTAAAA

Williams82 AAGAATTAAATATAATTTTTTTCCCACAAATTTAGCGTATGTTGTCTAGCTATACTAAAA

Blackhawk AAGAATTAAATATAATTTTTT-CCCACAAATTTAGCCTATGTTGTCTAGCTATACTAAAA

PI594891 GGGTAATTATTATAAATTAATACTTACAAGAAATTAGTATAAAATTAAAACATTTTTTTA

PI594774 GGGTAATTATTATAAATTAATACTTACAAGAAATTAGTATAAAATTAAAACATTTTTT-A

Williams82 GGGTAATTATTATAAATTAATACTTACAAGAAATTAGTATAAAATTAAAACATTTTTTTA

Blackhawk GGGTAATTATTATAAATTAATACTTACAAGAAATTAGTATAAAATTAAAACATTTTTT-A

PI594891 TTTTTTAAAGAGAAAAATACATTTTTATTATAACTCCTTGCTTTTATGTTTTGAAGCAC

PI594774 TTTTTTAAAGAGAAAAATAAATTTTTATTATAACTCCTTGCTTTTATGTTTTGAAGCAC

Williams82 TTTTTTAAAGAGAAAAATACATTTTTATTATAACTCCTTGCTTTTATGTTTTGAAGCAC

Blackhawk TTTTTTAAAGAGAAAAATAAATTTTTATTATAACTCCTTGCTTTTATGTTTTGAAGCAC

PI594891 TGAGTATGCATAAAAGTAACCGTACTTGGTGGACCCTGAATCCGCATTTTTATTCTTTTA

PI594774 TGAGTATGCATAAAAGTAACCGTACTTGGTGGACCCTGAATCCGCATTTTTATTCTTTTA

Williams82 CGAGTATGCATAAAAGTAACCGTACTTGGTGGACCCTGAATCCGCATTTTTATTCTTTTA

Blackhawk TGAGTATGCATAAAAGTAACCGTACTTGGTGGACCCTGAATCCGCATTTTTATTCTTTTA

PI594891 ATTAAAAGAGAATTTGAACCAAACCAACGTTCAAACCAAAAAAAAAAACAGTGACGT

PI594774 ATTAAAAGAGAATTTGAACCAAACCAACGTTCAAACCAAAAAAAAAAAAAGTGACGT

Williams82 ATTAAAAGAGAATTTGAACCAAACCAACGTTCAAACCAAAAAAAAAAACAGTGACGT

Blackhawk ATTAAAAGAGAATTTGAACCAAACCAACGTTCAAACCAAAAAAAAAAAAAAAGACGT

PI594891 TAGTGTTGTTGTTGTAGACTTGTAGTAGTTGTTGTTAGCTCTTGCTTTTGGAAAGAAAAG

PI594774 TAGTGTTGTTGTTGTAGACTTGTAGTAGTTGTTGTTAGCTCTTGCTTTTGGAAAGAAAAG

Williams82 TAGTGTTGTTGTTGTAGACTTGTAGTAGTTGTTGTTAGCTCTTGCTTTTGGAAAGAAAAG

Blackhawk TAGTGTTGTTGTTGTAGACTTGTAGTAGTTGTTGTTAGCTCTTGCTTTTGGAAAGAAAAG

PI594891 AAAGAAGTACAAAGCAGCAGAACAGAGTGTGTCTGTCTCCATCTGTATCCACTTTGGAA

PI594774 AAAGAAGTACAAAGCAGCAGAACAGAGTGTGTCTGTCTCCATCTGTATCCACTTTGGAA

Williams82 AAAGAAGTACAAAGCAGCAGAACAGAGTGTGTCTGTCTCCATCTGTATCCACTTTGGAA

Blackhawk AAAGAAGTACAAAGCAGCAGAACAGAGTGTGTCTGTCTCCATCTGTATCCACTTTGGAA

PI594891 CCATCTCTTCTTTTTTTCACTCACCACACCCATCTTCCGCTTTTCTCACACTTCAACT

PI594774 CCATCTCTTCTTTTTTTCACTCACCACACCCATCTTCCGCTTTTCTCACACTTCAACT

Williams82 CCATCTCTTCTTTTTTTCACTCACCACACCCATCTTCCGCTTTTCTCACACTTCAACT

Blackhawk CCATCTCTTCTTTTTTTCACTCACCACACCCATCTTCCGCTTTTCTCACACTTCAACT

PI594891 TTATTTGCCTCTGATCGCCAAAGGGCATGTTTCAATTCTCAGAATTACCCTCATTCTTAT

PI594774 TTATTTGCCTCTGATCGCCAAAGGGCATGTTTCAATTCTCAGAATTACCCTCATTCTTAT

Williams82 TTATTTGCCTCTGATCGCCAAAGGGCATGTTTCAATTCTCAGAATTACCCTCATTCTTAT

Blackhawk TTATTTGCCTCTGATCGCCAAAGGGCATGTTTCAATTCTCAGAATTACCCTCATTCTTAT

PI594891 CAAAGTTCATGCCTTTGTCTTGCTCTATTGTTTCAATTTTCAGCTTTGGAGGCTTGTGTG

PI594774 CAAAGTTCATGCCTTTGTCTTGCTCTATTGTTTCAATTTTCAGCTTTGGAGGCTTGTGTG

Williams82 CAAAGTTCATGCCTTTGTCTTGCTCTATTGTTTCAATTTTCAGCTTTGGAGGCTTGTGTG

Blackhawk CAAAGTTCATGCCTTTGTCTTGCTCTATTGTTTCAATTTTCAGCTTTGGAGGCTTGTGTG

PI594891 TCTGAAGGCATTGGAAAAAGGTGCTGAAAATTGAAAGCTGATTCTTTTGAAGAAATGGGT

PI594774 TCTGAAGGCATTGGAAAAAGGTGCTGAAAATTGAAAGCTGATTCTTTTGAAGAAATGGGT

Williams82 TCTGAAGGCATTGGAAAAAGGTACTGAAAATTGAAAGCTGATTCTTTTGAAGAAATGGGT

Blackhawk TCTGAAGGCATTGGAAAAAGGTGCTGAAAATTGAAAGCTGATTCTTTTGAAGAAATGGGT

PI594891 TGGTCTAATTCGGAGTTGAATTCGCAAATTTTAATCTTGTCTATGCTGATTTTCACTGCA

PI594774 TGGTCTAATTC**A**GAGTTGAATTCGCAAATTTTAATCTTGTCTATGCTGATTTTCACTGCA

Williams82 TGGTCTAATTCGGAGTTGAATTCGCAAATTTTAATCTTGTCTATGCTGATTTTCACTGCA

Blackhawk TGGTCTAATTCGGAGTTGAATTCGCAAATTTTAATCTTGTCTATGCTGATTTTCACTGCA

PI594891 AGTTTCTGTGTTGGGGACACTGACCCACTTGATGGTATGGTATAGTTCAGAGTTTTCATT

PI594774 AGTTTCTGTGTTGGGGACACTGACCCACTTGATGGTATGGTATAGTTCAGAGTTTTCATT

Williams82 AGTTTCTGTGTTGGGGACACTGACCCACTTGATGGTATGGTATAGTTCAGAGTTTTCATT

Blackhawk AGTTTCTGTGTTGGGGACACTGACCCACTTGATGGTATGGTATAGTTCAGAGTTTTCATT

PI594891 TTTCAGTTTTGATTGTAATTTAACTTTATGATGCTCTGTTTGGATGTGTTAGCTAAACGT

PI594774 TTTCAGTTTTGATTGTAATTTAACTTTATGATGCTCTGTTTGGATGTGTTAGCTAAACGT

Williams82 TTTCAGTTTTGATTGTAATTTAACTTTATGATGCTCTGTTTGGATGTGTTAGCTAAACGT

Blackhawk TTTCAGTTTTGATTGTAATTTAACTTTATGATGCTCTGTTTGGATGTGTTAGCTAAACGT

PI594891 TTTCTTTTAATGTGTTTATAGTTGCGGCAATCAATAGTTTGTACGTGGCTCTTGGCTCAC

PI594774 TTTCTTTTAATGTGTTTATAGTTGCGGCAATCAATAGTTTGTACGTGGCTCTTGGCTCAC

Williams82 TTTCTTTTAATGTGTTTATAGTTGCGGCAATCAATAGTTTGTACGTGGCTCTTGGCTCAC

Blackhawk TTTCTTTTAATGTGTTTATAGTTGCGGCAATCAATAGTTTGTACGTGGCTCTTGGCTCAC

PI594891 CACTCCTTGAGGGATGGAAAGCCACAGGAGGAGATCCTTGTTTGGAGCAATGGGAAGGTG

PI594774 CACTCCTTGAGGGATGGAAAGCCACAGGAGGAGATCCTTGTTTGGAGCAATGGGAAGGTG

Williams82 CACTCCTTGAGGGATGGAAAGCCACAGGAGGAGATCCTTGTTTGGAGCAATGGGAAGGTG

Blackhawk CACTCCTTGAGGGATGGAAAGCCACAGGAGGAGATCCTTGTTTGGAGCAATGGGAAGGTG

PI594891 TGAGTTGTGTCTTCTCCAACATTACTGCATTGTATGTCCCTCCTCACTTCCTTAAACTAT

PI594774 TGAGTTGTGTCTTCTCCAACATTACTGCATTGTATGTCCCTCCTCACTTCCTTAAACTAT

Williams82 TGAGTTGTGTCTTCTCCAACATTACTGCATTGTATGTCCCTCCTCACTTCCTTAAACTAT

Blackhawk TGAGTTGTGTCTTCTCCAACATTACTGCATTGTATGTCCCTCCTCACTTCCTTAAACTAT

PI594891 TTATTTGTTGTTTAGTCTTGTCTTGTGTTCTATTTTCTCAAAAAGTGTAATGTTTTTATG

PI594774 TTATTTGTTGTTTAGTCTTGTCTTGTGTTCTATTTTCTCAAAAAGTGTAATGTTTTTATG

Williams82 TTATTTGTTGTTTAGTCTTGTCTTGTGTTCTATTTTCTCAAAAAGTGTAATGTTTTTATG

Blackhawk TTATTTGTTGTTTAGTCTTGTCTTGTGTTCTATTTTCTCAAAAAGTGTAATGTTTTTATG

PI594891 TGGTGTTTTCTTGAGTTGTATAATTGATGAAGGTGAATGTGATCACTTGGTATATGGCTC

PI594774 TGGTGTTTTCTTGAGTTGTATAATTGATGAAGGTGAATGTGATCACTTGGTATATGGCTC

Williams82 TGGTGTTTTCTTGAGTTGTATAATTGATGAAGGTGAATGTGATCACTTGGTATATGGCTC

Blackhawk TGGTGTTTTCTTGAGTTGTATAATTGATGAAGGTGAATGTGATCACTTGGTATATGGCTC

PI594891 TATAAAATTTGTGTATTTGGGTTGGCAGACGGCTGGGAGGCATGGATTTAAGTGGAAAGC

PI594774 TATAAAATTTGTGTATTTGGGTTGGCAGACGGCTGGGAGGCATGGATTTAAGTGGAAAGC

Williams82 TATAAAATTTGTGTATTTGGGTTGGCAGACGGCTGGGAGGCATGGATTTAAGTGGAAAGC

Blackhawk TATAAAATTTGTGTATTTGGGTTGGCAGACGGCTGGGAGGCATGGATTTAAGTGGAAAGC

PI594891 TGGGAACTAATTTGGATTTCCCATCCATCATAGAAATGTAAGTGTGTTTTTTCAAGATA

PI594774 TGGGAACTAATTTGGATTTCCCATCCATCATAGAAATGTAAGTGTGTTTTTTCAAGATA

Williams82 TGGGAACTAATTTGGATTTCCCATCCATCATAGAAATGTAAGTGTGTTTTTTCAAGATA

Blackhawk TGGGAACTAATTTGGATTTCCCATCCATCATAGAAATGTAAGTGTGTTTTTTCAAGATA

PI594891 ATTGTAAATGTCATTAGCATGTGTGAATTTTTAGAATGAGGTAGTCTACAGTATTACATT

PI594774 ATTGTAAATGTCATTAGCATGTGTGAATTTTTAGAATGAGGTAGTCTACAGTATTACATT

Williams82 ATTGTAAATGTCATTAGCATGTGTGAATTTTTAGAATGAGGTAGTCTACAGTATTACATT

Blackhawk ATTGTAAATGTCATTAGCATGTGTGAATTTTTAGAATGAGGTAGTCTACAGTATTACATT

PI594891 TTTCATTTTTCCCTTGGTTACCATTGAAATTCTGAACTGAGGTCCTATTTGTTTGAATTC

PI594774 TTTCATTTTTCCCTTGGTTACCATTGAAATTCTGAACTGAGGTCCTATTTGTTTGAATTC

Williams82 TTTCATTTTTCCCTTGGTTACCATTGAAATTCTGAACTGAGGTCCTATTTGTTTGAATTC

Blackhawk TTTCATTTTTCCCTTGGTTACCATTGAAATTCTGAACTGAGGTCCTATTTGTTTGAATTC

PI594891 CAATTCAATGGTGACTAAAGCAGTCAATTTTTTTGTTTTGGTTGTTGAGGAATTTTATAG

PI594774 CAATTCAATGGTGACTAAAGCAGTCAATTTTTTTGTTTTGGTTGTTGAGGAATTTTATAG

Williams82 CAATTCAATGGTGACTAAAGCAGTCAATTTTTTTGTTTTGGTTGTTGAGGAATTTTATAG

Blackhawk CAATTCAATGGTGACTAAAGCAGTCAATTTTTTTGTTTTGGTTGTTGAGGAATTTTATAG

PI594891 CTCTAAACTAATAGTAGAAATAACAGTGGATGGTTTCACATATATGCATATTGAAAATGG

PI594774 CTCTAAACTAATAGTAGAAATAACAGTGGATGGTTTCACATATATGCATATTGAAAATGG

Williams82 CTCTAAACTAATAGTAGAAATAACAGTGGATGGTTTCACATATATGCATATTGAAAATGG

Blackhawk CTCTAAACTAATAGTAGAAATAACAGTGGATGGTTTCACATATATGCATATTGAAAATGG

PI594891 TAGCATTTCTTTTATATGCTTTATTGTTTAGGTTCCATAATTGTGCTATCATTTGCATGA

PI594774 TAGCATTTCTTTTATATGCTTTATTGTTTAGGTTCCATAATTGTGCTATCATTTGCATGA

Williams82 TAGCATTTCTTTTATATGCTTTATTGTTTAGGTTCCATAATTGTGCTATCATTTGCATGA

Blackhawk TAGCATTTCTTTTATATGCTTTATTGTTTAGGTTCCATAATTGTGCTATCATTTGCATGA

PI594891 AAACCTCAAGAAGCTTTCTGTTCCCCAATTGGGCAGGGATCTTAGCAACAACCAGATTGG

PI594774 AAACCTCAAGAAGCTTTCTGTTCCCCAATTGGGCAGGGATCTTAGCAACAACCAGATTGG

Williams82 AAACCTCAAGGAGCTTTCTGTTCCCCAATTGGGCAGGGATCTTAGCAACAACCAGATTGG

Blackhawk AAACCTCAAGAAGCTTTCTGTTCCCCAATTGGGCAGGGATCTTAGCAACAACCAGATTGG

PI594891 AGGGACCATTCCATTCACTTTGCCCCCTACTTTGAGGAACTTGTATGCTTTCTTTATCA

PI594774 AGGGACCATTCCATTCACTTTGCCCCCTACTTTGAGGAACTTGTATGCTTTCTTTATCA

Williams82 AGGGACCATTCCATTCACTTTGCCCCCTACTTTGAGGAACTTGTATGCTTTCTTTATCA

Blackhawk AGGGACCATTCCATTCACTTTGCCCCCTACTTTGAGGAACTTGTATGCTTTCTTTATCA

PI594891 TCAGTTCATCACCAATTTCTTGACTTGTTTTATTGTACTTGCTATTGACTAAATAATTTG

PI594774 TCAGTTCATCACCAATTTCTTGACTTGTTTTATTGTACTTGCTATTGACTAAATAATTTG

Williams82 TCAGTTCATCACCAATTTCTTGACTTGTTTTATTGTACTTGCTATTGACTAAATAATTTG

Blackhawk TCAGTTCATCACCAATTTCTTGACTTGTTTTATTGTACTTGCTATTGACTAAATAATTTG

PI594891 TATCTATATCCATAAATATGAGTATCCTTATGCAAGTTTCTGTTTTCTTTTTATAAGTTC

PI594774 TATCTATATCCATAAATATGAGTATCCTTATGCAAGTTTCTGTTTTCTTTTTATAAGTTC

Williams82 TATCTATATCCATAAATATGAGTTTTCTTATGCAAGTTTCTGTTTTCTTTTTATAAGTTC

Blackhawk TATCTATATCCATAAATATGAGTATCCTTATGCAAGTTTCTGTTTTCTTTTTATAAGTTC

PI594891 AGGTCACTATCATCAAATCAGTTAAATGGAAGCATTCCAGATGCCTTATCCTTGTTAACT

PI594774 AGGTCACTATCAACAAATCAGTTAAATGGAAGCATTCCAGATGCCTTATCCTTGTTAACT

Williams82 AGGTCACTATCATCAAATCAGTTAAATGGAAGCATTCCAGATGCCTTATCCTTGTTAACT

Blackhawk AGGTCACTATCATCAAATCAGTTAAATGGAAGCATTCCAGATGCCTTATCCTTGTTAACT

PI594891 CAATTGTCAGACTTGTGAGTTTATAGCATTGCCTCATCTCAATACTAAGTTGTTATATTT

PI594774 CAATTGTCAGACTTGTGAGTTTATAGCATTGCCTCATCTCAATACTAAGTTGTTATATTT

Williams82 CAATTGTCAGACTTGTGAGTTTATAGCATTGCCTCATCTCAATACTAAGTTGTTATATTT

Blackhawk CAATTGTCAGACTTGTGAGTTTATAGCATTGCCTCATCTCAATACTAAGTTGTTATATTT

PI594891 TTCCATAATCTGTTTCTTCATTTTGAATGAGAATATACTGCCACCTTTTAGGTCATTAAA

PI594774 TTCCATAATCTGTTTCTTCATTTTGAATGAGAATATACTGCCACCTTTTAGGTCATTAAA

Williams82 TTCCATAATCTGTTTCTTCATTTTGAATGAGAATATACTGCCACCTTTTAGGTCATTAAA

Blackhawk TTCCATAATCTGTTTCTTCATTTTGAATGAGAATATACTGCCACCTTTTAGGTCATTAAA

PI594891 AGATAACCATCTGAATGGACAAATCCCAAATGCATTTCTAGAACTCACTGGTTTGATGAA

PI594774 AGATAACCATCTGAATGGACAAATCCCAAATGCATTTCTAGAACTCACTGGTTTGATGAA

Williams82 AGATAACCATCTGAATGGACAAATCCCAAATGCATTTCTAGAACTCACTGGTTTGATGAA

Blackhawk AGATAACCATCTGAATGGACAAATCCCAAATGCATTTCTAGAACTCACTGGTTTGATGAA

PI594891 TTTGTAAGAAAATGTTGTTAAATGATAAATCACATTGATATTTCAGTGTCTTTACTTTGT

PI594774 TTTGTAAGAAAATGTTGTTAAATGATAAATCACATTGATATTTCAGTGTCTTTACTTTGT

Williams82 TTTGTAAGAAAATGTTGTTAAATGATAAATCACATTGATATTTCAGTGTCTTTACTTTGT

Blackhawk TTTGTAAGAAAATGTTGTTAAATGATAAATCACATTGATATTTCAGTGTCTTTACTTTGT

PI594891 CCACTATGACACAAATCCTGTGTTTTGCTGCAGGGATCTGTCTGGCAACAACTTGAGTGG

PI594774 CCACTATGACACAAATCCTGTGTTTTGCTGCAGGGATCTGTCTGGCAACAACTTGAGTGG

Williams82 CCACTATGACACAAATCCTGTGTTTTGCTGCAGGGATCTGTCTGGCAACAACTTGAGTGG

Blackhawk CCACTATGACACAAATCCTGTGTTTTGCTGCAGGGATCTGTCTGGCAACAACTTGAGTGG

PI594891 TAAGCTGCCGCCCTCAATGGGAAATTTGTCTTCTCTTATCACACTGTAAGTACAATTTTT

PI594774 TAAGCTGCCGCCCTCAATGGGAAATTTGTCTTCTCTTATCACACTGTAAGTACAATTTTT

Williams82 TAAGCTGCCGCCCTCAATGGGAAATTTGTCTTCTCTTATCACACTGTAAGTACAATTTTT

Blackhawk TAAGCTGCCGCCCTCAATGGGAAATTTGTCTTCTCTTATCACACTGTAAGTACAATTTTT

PI594891 AGCAACTAAGATTATATTTGCCTACTTGTTAAATGAACACATGTTTTTAAAATTTTATTT

PI594774 AGCAACTAAGATTATATTTGCCTACTTGTTAAATGAACACATGTTTTTATAATTTTATTT

Williams82 AGCAACTAAGATTATATTTGCCTACTTGTTAAATGAACACATGTTTTTAAAATTTTATTT

Blackhawk AGCAACTAAGATTATATTTGCCTACTTGTTAAATGAACACATGTTTTTAAAATTTTATTT

PI594891 ATTTTTGCAGAAACTTGCAGAACAATCAACTTTCTGGGACCCTTTTTGTTTTGCAGGACC

PI594774 ATTTTTGCAGAAACTTGCAGAACAATCAACTTTCTGGGACCCTTTTTGTTTTGCAGGACC

Williams82 ATTTTTGCAGAAACTTGCAGAACAATCAACTTTCTGGGACCCTTTTTGTTTTGCAGGACC

Blackhawk ATTTTTGCAGAAACTTGCAGAACAATCAACTTTCTGGGACCCTTTTTGTTTTGCAGGACC

PI594891 TCCCTCTTCAGGATCTGTATATTTCTCGGAAATCATATTCTCTTTACAAACATAGAAAT

PI594774 TCCCTCTTCAGGATCTGTATATTTCTCGGAAATCATATTCTCTTTACAAACATAGAAAT

Williams82 TCCCTCTTCAGGATCTGTATATTTCTCGGAAATCATATTCTCTTTACAAACATAGAAAT

Blackhawk TCCCTCTTCAGGATCTGTATATTTCTCGGAAATCATATTCTCTTTACAAACATAGAAAT

PI594891 TAGTCAAATGACTGTTGTCTATACCTAGCAACAATTTATGCTTAGGAAGTTTAAAGTGT

PI594774 TAGTCAAATGACTGTTGTCTATACCTAGCAACAATTTATGCTTAGGAAGTTTAAAGTGT

Williams82 TAGTCAAATGACTGTTGTCTATACCTAGCAACAATTTATGCTTAGGAAGTTTAAAGTGT

Blackhawk TAGTCAAATGACTGTTGTCTATACCTAGCAACAATTTATGCTTAGGAAGTTTAAAGTGT

PI594891 TTTAAATTTAATTTGTGAGTCTATAATAATGTAGGAACATAGAGAACAATATATTCTCTG

PI594774 TTTAAATTTAATTTGTGAGTCTATAATAATGTAGGAACATAGAGAACAATATATTCTCTG

Williams82 TTTAAATTTAATTTGTGAGTCTATAATAATGTAGGAACATAGAGAACAATATATTCTCTG

Blackhawk TTTAAATTTAATTTGTGAGTCTATAATAATGTAGGAACATAGAGAACAATATATTCTCTG

PI594891 GGCCAATTCCCCCAGAGTTGTTGAGTATCCCTAATTTCAGGTGAGTCATGGCTTACA

PI594774 GGCCAATTCCCCCAGAGTTGTTGAGTATCCCTAATTTCAGGTGAGTCATGGCTTACA

Williams82 GGCCAATTCCCCCAGAGTTGTTGAGTATCCCTAATTTCAGGTGAGTCATGGCTTACA

Blackhawk GGCCAATTCCCCCAGAGTTGTTGAGTATCCCTAATTTCAGGTGAGTCATGGCTTACA

PI594891 CTTTCTCTAGCATAGTTTTGTTAGCTAATCTCTTTCTCTCTCTCTCTCTCTCTCTATAAA

PI594774 CTTTCTCTAGCATAGTTTTGTTAGCTAATCTCTTTCTCTCTCTCTCTCTCTCTCTATAAA

Williams82 CTTTCTCTAGCATAGTTTTGTTAGCTAATCTCTTTCTCTCTCTCTCTCTCTCTCTATAAA

Blackhawk CTTTCTCTAGCATAGTTTTGTTAGCTAATCTCTTTCTCTCTCTCTCTCTCTCTCTATAAA

PI594891 TTGAAACCTTTGTTCAAGTATTATTATTTTGACCTTCAAGGAGATGTTACATTTGAAATT

PI594774 TTGAAACCTTTGTTCAAGTATTATTATTTTGACCTTCAAGGAGATGTTACATTTGAAATT

Williams82 TTGAAACCTTTGTTCAAGTATTATTATTTTGACCTTCAAGGAGATGTTACATTTGAAATT

Blackhawk TTGAAACCTTTGTTCAAGTATTATTATTTTGACCTTCAAGGAGATGTTACATTTGAAATT

PI594891 TGTACTTTAGTTTATTTCATGATGAGAATTGATGATAAAATGTAATGCAATATGCTAGCA

PI594774 TGTACTTTAGTTTATTTCATGATGAGAATTGATGATAAAATGTAATGCAATATGCTAGCA

Williams82 TGTACTTTAGTTTATTTCATGATGAGAATTGATGATAAAATGTAATGCAATATGCTAGCA

Blackhawk TGTACTTTAGTTTATTTCATGATAAGAATTGATGATAAAATGTAATGCAATATGCTAGCA

PI594891 TTATCCTTTTCCAGATGATCTAAGCATTTTAGTTCTGGCTTAGTTTCTTTGCATAACGCC

PI594774 TTATCCTTTTCCAGATGATCTAAGCATTTTAGTTCTGGCTTAGTTTCTTTGCATAACGCC

Williams82 TTATCCTTTTCCAGATGATCTAAGCATTTTAGTTCTGGCTTAGTTTCTTTGCATAACGCC

Blackhawk TTATCCTTTTCCAGATGATCTAAGCATTTTAGTTCTGGCTTAGTTTCTTTGCATAACGCC

PI594891 TTAGGATAAAATAAATTCATCCAAGTTAATTATAGTAGCATTTACTTTGCAGAAAAGATG

PI594774 TTAGGATAAAATAAATTCATCCAAGTTAATTATAGTAGCATTTACTTTGCAGAAAAGATG

Williams82 TTAGGATAAAATAAATTCATCCAAGTTAATTATAGTAGCATTTACTTTGCAGAAAAGATG

Blackhawk TTAGGATAAAATAAATTCATCCAAGTTAATTATAGTAGCATTTACTTTGCAGAAAAGATG

PI594891 GAAATCCATTTAATACTACTATCATTCCATCACCTCCAGCTGCTTTCCCAGCACCTGCTG

PI594774 GAAATCCATTTAATACTACTATCATTCCATCACCTCCAGCTGCTTTCCCAGCACCTGCTG

Williams82 GAAATCCATTTAATACTACTATCATTCCATCACCTCCAGCTGCTTTCCCAGCACCTGCTG

Blackhawk GAAATCCATTTAATACTACTATCATTCCATCACCTCCAGCTGCTTTCCCAGCACCTGCTG

PI594891 CTATGGCTCCTTCCCCAGAAAAATCACCTTGGAAAATGGCACATAATCCCTCTGATACC

PI594774 CTATGGCTCCTTCCCCAGAAAAATCACCTTGGAAAATGGCACATAATCCCTCTGATACC

Williams82 CTATGGCTCCTTCCCCAGAAAAATCACCTTGGAAAATGGCACATAATCCCTCTGATACC

Blackhawk CTATGGCTCCTTCCCCAGAAAAATCACCTTGGAAAATGGCACATAATCCCTCTGATACC

PI594891 ATAAAAGCACCAATACCTGCAATTGCTGGGAGATCTTTCAAAACTACAAAATTAGTTTG

PI594774 ATAAAAGCACCAATACCTGCAATTGCTGGGAGATCTTTCAAAACTACAAAATTAGTTTG

Williams82 ATAAAAGCACCAATACCTGCAATTGCTGGGAGATCTTTCAAAACTACAAAATTAGTTTG

Blackhawk ATAAAAGCACCAATACCTGCAATTGCTGGGAGATCTTTCAAAACTACAAAATTAGTTTG

PI594891 GATTGTTGGTGCAGGCTTTTTGATATTTATTGCATTAGGAGTCTGTCTTCTAATGCTG

PI594774 GATTGTTGGTGCAGGCTTTTTGATATTTATTGCATTAGGAGTCTGTCTTCTAATGCTG

Williams82 GATTGTTGGTGCAGGCTTTTTGATATTTATTGCATTAGGAGTCTGTCTTCTAATGCTG

Blackhawk GATTGTTGGTGCAGGCTTTTTGATATTTATTGCATTAGGAGTCTGTCTTCTAATGCTG

PI594891 TGGTGCTTTAAGAGAAGACAGGAAAACAAGAAATATAAGAAACACAACACGAATATGTAT

PI594774 TGGTGCTTTAAGAGAAGACAGGAAAACAAGAAATATAAGAAACACAACACGAATATGTAT

Williams82 TGGTGCTTTAAGAGAAGACAGGAAAACAAGAAATATAAGAAACACAACACGAATATGTAT

Blackhawk TGGTGCTTTAAGAGAAGACAGGAAAACAAGAAATATAAGAAACACAACACGAATATGTAT

PI594891 ACAAGATCTTTGCATAAACGTACATGCAGTAACTCCCCTTTTGAAGCAACTAATGATGAG

PI594774 ACAAGATCTTTGCATAAACGTACATGCAGTAACTCCCCTTTTGAAGCAACTAATGATGAG

Williams82 ACAAGATCTTTGCATAAACGTACATGCAGTAACTCCCCTTTTGAAGCAACTAATGATGAG

Blackhawk ACAAGATCTTTGCATAAACGTACATGCAGTAACTCCCCTTTTGAAGCAACTAATGATGAG

PI594891 GAGAAAGGTAAATTTGATGATGAAGCTCCTATTCTTTTAGTTTGAGCTGTTGTAACATAA

PI594774 GAGAAAGGTAAATTTGATGATGAAGCTCCTATTCTTTTAGTTTGAGCTGTTGTAACATAA

Williams82 GAGAAAGGTAAATTTGATGATGAAGCTCCTATTCTTTTAGTTTGAGCTGTTGTAACATAA

Blackhawk GAGAAAGGTAAATTTGATGATGAAGCTCCTATTCTTTTAGTTTGAGCTGTTGTAACATAA

PI594891 CTTCTTAAACTCTATTTGCAATTCAAAATAGAAGAACCAAATTGATTCCAAAGTTGCAG

PI594774 CTTCTTAAACTCTATTTGCAATTCAAAATAGAAGAACCAAATTGATTCCAAAGTTGCAG

Williams82 CTTCTTAAACTCTATTTGCAATTCAAAATAGAAGAACCAAATTGATTCCAAAGTTGCAG

Blackhawk CTTCTTAAACTCTATTTGCAATTCAAAATAGAAGAACCAAATTGATTCCAAAGTTGCAG

PI594891 GATGAACAAGAAGTACTTGTGAAAAGAATTTCTTTCAATTTCAGAATGCAATAACATTTA

PI594774 GATGAACAAGAAGTACTTGTGAAAAGAATTTCTTTCAATTTCAGAATGCAATAACATTTA

Williams82 GATGAACAAGAAGTACTTGTGAAAAGAATTTCTTTCAATTTCAGAATGCAATAACATTTA

Blackhawk GATGAACAAGAAGTACTTGTGAAAAGAATTTCTTTCAATTTCAGAATGCAATAACATTTA

PI594891 TGAATTAAACAATACAGAATGGAGTTCCAAACTTCCACCCCTGCAGCCAGCACCACCACA

PI594774 TGAATTAAACAATACAGAATGGAGTTCCAAACTTCCACCCCTGCAGCCAGCACCACCACA

Williams82 TGAATTAAACAATACAGAATGGAGTTCCAAACTTCCACCCCTGCAGCCAGCACCACCACA

Blackhawk TGAATTAAACAATACAGAATGGAGTTCCAAACTTCCACCCCTGCAGCCAGCACCACCACA

PI594891 TCATATCCCAATCATTCCTGGAGAGAATTTAATAATCAATCCAGCTATATCTACCCAAGC

PI594774 TCATATCCCAATCATTCCTGGAGAGAATTTAATAATCAATCCAGCTATATCTACCCAAGC

Williams82 TCATATCCCAATCATTCCTGGAGAGAATTTAATAATCAATCCAGCTATATCTACCCAAGC

Blackhawk TCATATCCCAATCATTCCTGGAGAGAATTTAATAATCAATCCAGCTATATCTACCCAAGC

PI594891 AGCTGAAAGACAAATTGTCACAAATTCCATTAAAGTTTATACTGTTGCATCACTTCAGCA

PI594774 AGCTGAAAGACAAATTGTCACAAATTCCATTAAAGTTTATACTGTTGCATCACTTCAGCA

Williams82 AGCTGAAAGACAAATTGTCACAAATTCCATTAAAGTTTATACTGTTGCATCACTTCAGCA

Blackhawk AGCTGAAAGACAAATTGTCACAAATTCCATTAAAGTTTATACTGTTGCATCACTTCAGCA

PI594891 GTACACAAATAGTTTTTCCCAAGAAAATTATATTGGGGAAGGCATGCTTGGCCCTGTTTA

PI594774 GTACACAAATAGTTTTTCCCAAGAAAATTATATTGGGGAAGGCATGCTTGGCCCTGTTTA

Williams82 GTACACAAATAGTTTTTCCCAAGAAAATTATATTGGGGAAGGCATGCTTGGCCCTGTTTA

Blackhawk GTACACAAATAGTTTTTCCCAAGAAAATTATATTGGGGAAGGCATGCTTGGCCCTGTTTA

PI594891 CAGGGCTGAACTCCCTGATGGAAAGGTAATATTCTTCTAGTTTAATGTATTTGGGCATTT

PI594774 CAGGGCTGAACTCCCTGATGGAAAGGTAATATTCTTCTAGTTTAATGTATTTGGGCATTT

Williams82 CAGGGCTGAACTCCCTGATGGAAAGGTAATATTCTTCTAGTTTAATGTATTTGGGCATTT

Blackhawk CAGGGCTGAACTCCCTGATGGAAAGGTAATATTCTTCTAGTTTAATGTATTTGGGCATTT

PI594891 TTTAATGTTCATTTCTTTGCTAACTTGGCCTTGATTCCTATGGAGGGAAAGGGAAGGTAG

PI594774 TTTAATGTTCATTTCTTTGCTAACTTGGCCTTAATTCCTATGGAGGGAAAGGAAAGGTAG

Williams82 TTTAATGTTCATTTCTTTGCTAACTTGGCCTTGATTCCTATGGAGGGAAAGGGAAGGTAG

Blackhawk TTTAATGTTCATTTCTTTGCTAACTTGGCCTTGATTCCTATGGAGGGAAAGGGAAGGTAG

PI594891 GGTTAAATTTTCAATTTGAACATTTTTTTAAATGAAAGGAATAAAACCAGATATTTGTT

PI594774 GGTTAAATTTTCAATTTGAACATTTTTTTAAATGAAAGGAATAAAACCAGATATTTGTT

Williams82 GGTTAAATTTTCAATTTGAACATTTTTTTAAATGAAAGGAATAAAACCAGATATTTGTT

Blackhawk GGTTAAATTTTCAATTTGAACATTTTTTTAAATGAAAGGAATAAAACCAGATATTTGTT

PI594891 CATCTTCTGTTGCCCCCTCTCAATAGTTTGAACAGTAATTCTTTTCCTCTACTACCCTTA

PI594774 CATCTTCTGTTGCCCCCTCTCAATAGTTTGAACAGTAATTCTTTTCCTCTACTACCCTTA

Williams82 CATCTTCTGTTGCCCCCTCTCAATAGTTTGAACAGTAATTCTTTTCCTCTACTACCCTTA

Blackhawk CATCTTCTGTTGCCCCCTCTCAATAGTTTGAACAGTAATTCTTTTCCTCTACTACCCTTA

PI594891 ATAAATTATGAAGATTATTTTGAAGTTTAAACATTGCTTGTTATTAGGAATCTAGTACTT

PI594774 ATAAATTAAGAAGATTATTTTGAAGTTTAAACATTGCTTGTTATTAGGAATCTAGTACTT

Williams82 ATAAATTAAGAAGATTATTTTGAAGTTTAAACATTGCTTGTTATTAGGAATCTAATACTT

Blackhawk ATAAATTATGAAGATTATTTTGAAGTTTAAACATTGCTTGTTATTAGGAATCTAGTACTT

PI594891 TGTGATGTTTGCTGATTTTTCCTGTTGTGCTTGCTTGCCAATGTTGCCATGACCTGTTAT

PI594774 TGTGATGTTTGCTGATTTTTCCTGTTGTGCTTGCTTGCCAATGTTGCCATGACCTGTTAT

Williams82 TGTGATGTTTGCTGATTTTTCCTGTTGTGCTTGCTTGCCAATGTTGCCATGACCTGTTAT

Blackhawk TGTGATGTTTGCTGATTTTTCCTGTTGTGCTTGCTTGCCAATGTTGCCATGACCTGTTAT

PI594891 TATTCTCCTAGTTTCACTTATTTAACTGGAGCTTCATATGCTTATATATTTCAAATAATC

PI594774 TATTCTCCTAGTTTCACTTATTTAACTGGAGCTTCATATGCTTATATATTTCAAATAATC

Williams82 TATTCTCCTAGTTTCACTTATTTAACTGGAGCTTCATATGCTTATATATTTCAAATAATC

Blackhawk TATTCTCCTAGTTTCACTTATTTAACTGGAGCTTCATATGCTTATATATTTCAAATAATC

PI594891 TTCTTTGTAGTTACTGGCAGTTAGGAAGATGAATACTACTGCTTCTATGGGGCAAAATCA

PI594774 TTCTTTGTAGTTACTGGCAGTTAGGAAGATGAATACTACTGCTTCTATGGGGCAAAATCA

Williams82 TTCTTTGTAGTTACTGGCAGTTAGGAAGATGAATACTACTGCTTCTATGGGGCAAAATCA

Blackhawk TTCTTTGTAGTTACTGGCAGTTAGGAAGATGAATACTACTGCTTCTATGGGGCAAAATCA

PI594891 TGAACAGTTTCTTCAATTAGTTTTCAGTATCTCCAAAATTCAACATGCTAATATTGTTAA

PI594774 TGAACAGTTTCTTCAATTAGTTTTCAGTATCTCCAAAATTCAACATGCTAATATTGTTAA

Williams82 TGAACAGTTTCTTCAATTAGTTTTCAGTATCTCCAAAATTCAACATGCTAATATTGTTAA

Blackhawk TGAACAGTTTCTTCAATTAGTTTTCAGTATCTCCAAAATTCAACATGCTAATATTGTTAA

PI594891 GCTTATGGGCTACTGTGCTGAGTATAGTCAACGACTACTTGTACATGAGTATTGCAATAA

PI594774 GCTTATGGGCTACTGTGCTGAGTATAGTCAACGACTACTTGTACATGAGTATTGCAATAA

Williams82 GCTTATGGGCTACTGTGCTGAGTATAGTCAACGACTACTTGTACATGAGTATTGCAATAA

Blackhawk GCTTATGGGCTACTGTGCTGAGTATAGTCAACGACTACTTGTACATGAGTATTGCAATAA

PI594891 TGGTACCCTACATGAAGCATTGCATACAGATGATAAACTTCAGATTAAACTTTCATGGGA

PI594774 TGGTACCCTACATGAAGCATTGCATACAGATGATAAACTTCAGATTAAACTTTCATGGGA

Williams82 TGGTACCCTACATGAAGCATTGCATACAGATGATAAACTTCAGATTAAACTTTCATGGGA

Blackhawk TGGTACCCTACATGAAGCATTGCATACAGATGATAAACTTCAGATTAAACTTTCATGGGA

PI594891 TGATCGCATTCAGGTGTCACTTGGAGCTGCAAGAGCTTTGGAGTAAGTTTAGCAATTAGT

PI594774 TGATCGCATTCAGGTGTCACTTGGAGCTGCAAGAGCTTTGGAGTAAGTTTAGCAATTAGT

Williams82 TGATCGCATTCAGGTGTCACTTGGAGCTGCAAGAGCTTTGGAGTAAGTTTAGCAATTAGT

Blackhawk TGATCGCATTCAGGTGTCACTTGGAGCTGCAAGAGCTTTGGAGTAAGTTTAGCAATTAGT

PI594891 NACAAATTCAATAACTCTTTCATGTAACTAGATAAACTCAATATTGGTTAGTGAACTATG

PI594774 CACAAATTCAATAACTCTTTCATGTAACTAGATAAACTCAATATTGGTTAGTGAACTATG

Williams82 CACAAATTCAATAACTCTTTCATGAGCCAGAGGAAGCTTTAGACACAAGGAAGAATGAAC

Blackhawk CACAAATTCAATAACTCTTTCATGTAACTAGATAAACTCAATATTGGTTAGTGAACTATG

PI594891 CTTGTCTTGTTCAGTTTAGCATTAGTCACAAATTCAATAACTCTTTCATGTAACTAGATA

PI594774 CTTGTCTTGTTCAGTTTAGCATTAGTCACAAATTCAATAACTCTTTCATGTAACTAGATA

Williams82 AGGGAGTAGTGGAAGTCTTGGTGAAATGGAAAGACCTTCCAGACTTTGAAAATTCCTGGG

Blackhawk CTTGTCTTGTTCAGTTTAGCATTAGTCACAAATTCAATAACTCTTTCATGTAACTAGATA

PI594891 AACTCAATATTGGTTAGTGAACTATGCTTGTCTTGTTCTGGAGGTTCGAAACATAACTTT

PI594774 AACTCAATATTGGTTAGTGAACTATGCTTGTCTTGTTCTGGAGGTTCGAAACATAACTTT

Williams82 AGCCAGTGGAAAAGCTCAGATCAGAATTTCCTGGATTTCTTCTTGAGGGCAAGAAGAGTT

Blackhawk AACTCAATATTGGTTAGTGAACTATGCTTGTCTTGTTCTGGAGGTTCGAAACATAACTTT

PI594891 TATATGTTAAAAGTATTATTAAGAATATCTT-----------------------------

PI594774 TATATGTTAAAAGTATTATTAAGAATATCTT-----------------------------

Williams82 TTGAAGGCGGGGGA----------------------------------------------

Blackhawk TATATGTTAAAAGTATTATTAAGAATATCTT-----------------------------

PI594891 ------------------------------------------------------------

PI594774 ------------------------------------------------------------

Williams82 ATTGATAAGCAACAGATAGTATACACGAGGAAACATGGTAAGGGAAGTGTAGGGGATCAT

Blackhawk ------------------------------------------------------------

PI594891 ------------------------------------------------------------

PI594774 ------------------------------------------------------------

Williams82 CCCACTAGAGGAGTAACTAACTTAGTGGGGAAGGTGTGATGGGACAGGTGGCAGAGAGAG

Blackhawk ------------------------------------------------------------

PI594891 ------------------------------------------------------------

PI594774 ------------------------------------------------------------

Williams82 AAAGGAGGGCTGTAACGTTATATGCTGAGGGAATAATCAGTAGTGGGGCATAAGGAGGGC

Blackhawk ------------------------------------------------------------

PI594891 ------------------------------------------------------------

PI594774 ------------------------------------------------------------

Williams82 TGTAACGTTATATGCTGAGGGAATAATCAGTAGTGGGGCATGTATGGATTCAGTTAGAAA

Blackhawk ------------------------------------------------------------

PI594891 ------------------------------------------------------------

PI594774 ------------------------------------------------------------

Williams82 CTGCATGGGAGTTTTCTAAGCAGCTCGAAATGCTTAGCCTGTTTTCTGCATTTTTTCATT

Blackhawk ------------------------------------------------------------

PI594891 ------------------------------------------------------------

PI594774 ------------------------------------------------------------

Williams82 TCCATTGTTCATCCATTGTAATTCACAAGTTAGATCCCATTTGAACTCTAACTTGATTTA

Blackhawk ------------------------------------------------------------

PI594891 AGGTCTTGCAATTTGTACAAGTTTTGCAAGGTGGTTTTTACACATATGATGTGAAATTGG

PI594774 AGGTCTTGCAATTTGTACAAGTTTTGCAAGGTGGTTTTTACACATATGATGTGAAATTGG Williams82 CATCAATAATATCTCCCATTTTCATCTTAAAATTGTTATTTTACTGTTTTATTACTTCTG

Blackhawk AGGTCTTGCAATTTGTACAAGTTTTGCAAGGTGGTTTTTACACATATGATGTGAAATTGG

PI594891 CATATGATGTGAAATTGGATCTCCATCTACCTATAAAGATATTATGTCCTAGACTATTAA

PI594774 CATATGATGTGAAATTGGATCTCCATCTACCTATAAAGATATTATGTCCTAGACTATTAA

Williams82 GATATCAGATTGAGACCTATCACCATCTACCTATAAAGATATTATGTCCTAGACTATTAA

Blackhawk CATATGATGTGAAATTGGATCTCCATCTACCTATAAAGATATTATGTCCTAGACTATTAA

PI594891 CACTAGGTTAAGGCATTGGTGATTTTCTTGATAACCTTAATTGATCATCTCCTTTTTTCA

PI594774 CACTAGGTTAAGGCATTGGTGATTTTCTTGATAACCTTAATTGATCATCTCCTTTTTTCA

Williams82 CACTAGGTTAAGGCATTGGTGATTTTCTTGATAACCTTAATTGATCATCTCCTTTTTTCA

Blackhawk CACTAGGTTAAGGCATTGGTGATTTTCTTGATAACCTTAATTGATCATCTCCTTTTTTCA

PI594891 TGAGCTAGGTACTTGCATGAGCACTGTCAACCACCTATTGTGCACCGAAACTTTAGGTC

PI594774 TGTGCTAGGTACTTGCATGAGCACTGTCAACCACCTATTGTGCACCGAAACTTTAGGTC

Williams82 TGTGCTAGGTACTTGCATGAGCACTGTCAACCACCTATTGTGCACCGAAACTTTAGGTC

Blackhawk TGAGCTAGGTACTTGCATGAGCACTGTCAACCACCTATTGTGCACCGAAACTTTAGGTC

PI594891 TGCTAATATACTCCTCAATGACAAATTGGAAGTGCTTGTCTCTGATTGTGGATTAGGTT

PI594774 TGCTAATATACTCCTCAATGACAAATTGGAAGTGCTTGTCTCTGATTGTGGATTAGGTT

Williams82 TGCTAATATACTCCTCAATGACAAATTGGAAGTGCTTGTCTCTGATTGTGGATTAGGTT

Blackhawk TGCTAATATACTCCTCAATGACAAATTGGAAGTGCTTGTCTCTGATTGTGGATTAGGTT

PI594891 CTTTACTATCTTCAGGCTTTGCTAGTCAGGTAACACTTCAGCTTTAATTTCTTCACATAA

PI594774 CTTTACTATCTTCAGGCTCTGCTAGTCAGGTAACACTTCAGCTTTAATTTCTTCACATAA

Williams82 CTTTACTATCTTCAGGCTCTGCTAGTCAGGTAACACTTCAGCTTTAATTTCTTCACATAA

Blackhawk CTTTACTATCTTCAGGCTTTGCTAGTCAGGTAACACTTCAGCTTTAATTTCTTCACATAA

PI594891 TCTTCAGCTGATATATAGTTTGGGGATTTGGATTCCTGGTTCTTTTTGTGCTTTTAAAAT

PI594774 TCTTCAGCTGATATATAGTTTGGGGATTTGGATTCCTGGTTCTTTTTGTGCTTTTAAAAT

Williams82 TCTTCAGCTGATATATAGTTTGGGGATTTGGATTCCTGGTTCTTTTTGTGCTTTTAAAAT

Blackhawk TCTTCAGCTGATATATAGTTTGGGGATTTGGATTCCTGGTTCTTTTTGTGCTTTTAAAAT

PI594891 ATGCTTTTGACATTCTAGAATTGCTATTGAACAAGATGAAATAACTGGCCTAAATATGAA

PI594774 ATGCTTTTGACATTCTAGAATTGCTATTGAACAAGATGAAATAACTGGCCTAAATATGAA

Williams82 ATGCTTTTGACATTCTAGAATTGCTATTGAACAAGATGAAATAACTGGCCTAAATATGAA

Blackhawk ATGCTTTTGACATTCTAGAATTGCTATTGAACAAGATGAAATAACTGGCCTAAATATGAA

PI594891 TGTATAGAAAACATGTGGTTGAATTTTACTTTATTTTATTTTTAAAATTAACTTTTCTCT

PI594774 TGTATAGAAAACATGTGGTTGAATTTTACTTTATTTTATTTTTAAAATTAACTTTTCTCT

Williams82 TGTATAGAAAACATGTGGTTGAATTTTACTTTATTTTATTTTTAAAATTAACTTTTCTCT

Blackhawk TGTATAGAAAACATGTGGTTGAATTTTACTTTATTTTATTTTTAAAATTAACTTTTCTCT

PI594891 ATCTTTGTTGCATTTCTGGCTAACACTAGTACATCTTCTTTTAGTTGTTGGGACGCCACC

PI594774 ATCTTTGTTGCATTTCTGGCTAACACTAGTACATCTTCTTTTAGTTGTTGGGACGCCACC

Williams82 ATCTTTGTTGCATTTCTGGCTAACACTAGTACATCTTCTTTTAGTTGTTGGGACGCCACC

Blackhawk ATCTTTGTTGCATTTCTGGCTAACACTAGTACATCTTCTTTTAGTTGTTGGGACGCCACC

PI594891 TCACAGCTAATGGTTACAGTGCTCCAGAATTTGAGTATGGAAGTTATACATTGCAAAGT

PI594774 TCACAGCTAATGGTTACAGTGCTCCAGAATTTGAGTATGGAAGTTATACATTGCAAAGT

Williams82 TCACAGCTAATGGTTACAGTGCTCCAGAATTTGAGTATGGAAGTTATACATTGCAAAGT

Blackhawk TCACAGCTAATGGTTACAGTGCTCCAGAATTTGAGTATGGAAGTTATACATTGCAAAGT

PI594891 GATGTCTTTAGCTTTGGAGTTGTAATGCTAGAACTCCTCACTGGACGAAAATCCTTTGA

PI594774 GATGTCTTTAGCTTTGGAGTTGTAATGCTAGAACTCCTCACTGGACGAAAATCCTTTGA

Williams82 GATGTCTTTAGCTTTGGAGTTGTAATGCTAGAACTCCTCACTGGACGAAAATCCTTTGA

Blackhawk GATGTCTTTAGCTTTGGAGTTGTAATGCTAGAACTCCTCACTGGACGAAAATCCTTTGA

PI594891 CAGGTTAGCAATCCCATTAGCAAGTTCGATTAGATACTTGTTTTATAATGTCATATTTAA

PI594774 CAGGTTAGCAATCCCATTAGCAAGTTCGATTAGATACTTGTTTTATAATGTCATATTTAA

Williams82 CAGGTTAGCAATCCCATTAGCAAGTTCGATTAGATACTTGTTTTATAATGTCATATTTAA

Blackhawk CAGGTTAGCAATCCCATTAGCAAGTTCGATTAGATACTTGTTTTATAATGTCATATTTAA

PI594891 AGTTTTGAATTTGGCTAAGGCACCATTGATTATGTATTGGTATAGCTCACGGCCTCGTGT

PI594774 AGTTTTGAATTTGGCTAAGGCACCATTGATTATGTATTGGTATAGCTCACGGCCTCGTGT

Williams82 AGTTTTGAATTTGGCTAAGGCACCATTGATTATGTATTGGTATAGCTCACGGCCTCGTGT

Blackhawk AGTTTTGAATTTGGCTAAGGCACCATTGATTATGTATTGGTATAGCTCACGGCCTCGTGT

PI594891 AGAGCAATTTTTGATGAGATGGGCAATCCCTCAACTCCATGACATAGATGCATTGTCAAA

PI594774 AGAGCAATTTTTGATGAGATGGGCAATCCCTCAACTCCATGACATAGATGCATTGTCAAA

Williams82 AGAGCAATTTTTGATGAGATGGGCAATCCCTCAACTCCATGACATAGATGCATTGTCAAA

Blackhawk AGAGCAATTTTTTATGAGATGGGCAATCCCTCAACTCCATGACATAGATGCATTGTCAAA

PI594891 AATGGTTGACCCCTCCTTGAATGGAGAATATCCTAAGAAATCCTTGTCGCGTTTTGCAGA

PI594774 AATGGTTGACCCCTCCTTGAATGGAGAATATCCTAAGAAATCCTTGTCGCGTTTTGCAGA

Williams82 AATGGTTGACCCCTCCTTGAATGGAGAATATCCTAAGAAATCCTTGTCGCGTTTTGCAGA

Blackhawk AATGGTTGACCCCTCCTTGAATGGAGAATATCCTAAGAAATCCTTGTCGCGTTTTGCAGA

PI594891 CATTATTTCTTCATGTATTCAGGTAATTTTCTTCATGTATCATGGTACATTGCACAAATG

PI594774 CATTATTTCTTCATGTATTCAGGTAATTTTCTTCATGTATCATGGTACATTGCACAAATG

Williams82 CATTATTTCTTCATGTATTCAGGTAATTTTCTTCATGTATCATGGTACATTGCACAAATG

Blackhawk CATTATTTCTTCATGTATTCAGGTAATTTTCTTCATGTATCATGGTACATTGCACAAATG

PI594891 TAATCTTTAATGATTAAATATTGAGGCATGCTTTTCACCAAAAACAAAAAAACAAAGATT

PI594774 TAATCTTTAATGATTAAATATTGAGGCATGCTTTTCACCAAAAACAAAAAAACAAAGATT

Williams82 TAATCTTTAATGATTAAATATTGAGGCATGCTTTTCACCAAAAACAAAAAAACAAAGATT

Blackhawk TAATCTTTAATGATTAAATATTGAGGCATGCTTTTCACCAAAAACAAAAAAACAAAGATT

PI594891 TCACCAAAAAATTACTGAGGTGTGCTTTACATTTTAACCCTGCCTTTTGTAGTCTCCAAA

PI594774 TCACCAAAAAATTACTGAGGTGTGCTTTACATTTTAACCCTGCCTTTTGTAGTCTCCAAA

Williams82 TCACCAAAAAATTACTGAGGTGTGCTTTACATTTTAACCCTGCCTTTTGTAGTCTCCAAA

Blackhawk TCACCAAAAAATTACTGAGGTGTGCTTTACATTTTAACCCTGCCTTTTGTAGTCTCCAAA

PI594891 TTCTTGTCTAGTTTAATTTGAAATGTGCAAACCATAACATGAATTGTCTATATTGAATAT

PI594774 TTCTTGTCTAGTTTAATTTGAAATGTGCAAACCATAACATGAATTGTCTATATTGAATAT

Williams82 TTCTTGTCTAGTTTAATTTGAAATGTGCAAACCATAACATGAATTGTCTATATTGAATAT

Blackhawk TTCTTGTCTAGTTTAATTTGAAATGTGCAAACCATAACATGAATTGTCTATATTGAATAT

PI594891 ACAAGGACCATTCAAGGAATATGGTGAGGTCTGGAATTAGAACTATACTTCAATATT

PI594774 ACAAGGACCATTCAAGGAATATGGTGAGGTCTGGAATTAGAACTATACTTCAATATT

Williams82 ACAAGGACCATTCAAGGAATATGGTGAGGTCTGGAATTAGAACTATACTTCAATATT

Blackhawk ACAAGGACCATTCAAGGAATATGGTGAGGTCTGGAATTAGAACTATACTTCAATATT

PI594891 GTCATTATTTCACATGTTCTAATATATATTGGTCGATGCAGTTTATCTACTCTCTTATCA

PI594774 GTCATTATTTCACATGTTCTAATATATATTGGTCGATGCAGTTTATCTACTCTCTTATCA

Williams82 GTCATTATTTCACATGTTCTAATATATATTGGTCGATGCAGTTTATCTACTCTCTTATCA

Blackhawk GTCATTATTTCACATGTTCTAATATATATTGGTCGATGCAGTTTATCTACTCTCTTATCA

PI594891 CTTTCTTATGGCCCATAATTTTTATTTTT------TCAATTGTTTTTCTTGCAGAATGAA

PI594774 CTTTCTTATGGCCCATAATTTTTATTTTTATTTTTTCAATTGTTTTTCTTGCAGAATGAA

Williams82 CTTTCTTATGGCCCATAATTTTTATTTTTATTTTTTCAATTGTTTTTCTTGCAGAATGAA

Blackhawk CTTTCTTATGGCCCATAATTTTTATTTTTATTTTTTCAATTGTTTTTCTTGCAGAATGAA

PI594891 CCTGAATTTCGACCAGCAATGTCAGAAATTGTTCAGGATCTTTTGCGAATGATGTAGAGG

PI594774 CCTGAATTTCGACCAGCAATGTCAGAAATTGTTCAGGATCTTTTGCGAATGATGTAGAGG

Williams82 CCTGAATTTCGACCAGCAATGTCAGAAATTGTTCAGGATCTCTTGCGAATGATGTAGAGG

Blackhawk CCTGAATTTCGACCAGCAATGTCAGAAATTGTTCAGGATCTTTTGCGAATGATGTAGAGG

PI594891 GCCGTGTAGCTGTTAATGGGGTGATGAACAGCCTCAGCCAGTAAAAGAAGCTATGGTTAC

PI594774 GCCGTGTAGCTGTTAATGGGGTGATGAACAGCCTCAGCCAGTAAAAGAAGCTATGGTTAC

Williams82 GCCGTGTAGCTGTTAATGGGGTGATGAACAGCCTCAGCCAGTAAAAGAAGCTATGGTTAC

Blackhawk GCCGTGTAGCTGTTAATGGGGTGATGAACAGCCTCAGCCAGTAAAAGAAGCTATGGTTAC

PI594891 CAAATTTTCTGGATGTTACATTTTCATTCTTTTATTTTTTCAGTGACATCAATCTTCCAT

PI594774 CAAATTTTCTGGATGTTACATTTTCATTCTTTTATTTTTTCAGTGACATCAATCTTCCAT

Williams82 CAAATTTTCTGGATGTTACATTTTCATTCTTTTATTTTTTCAGTGACATCAATCTTCCAT

Blackhawk CAAATTTTCTGGATGTTACATTTTCATTCTTTTATTTTTTCAGTGACATCAATCTTCCAT

PI594891 TATGTGTGATTATGCTTATTAATGTGATTTTATTGAGTTTGTATAGTGTTATGTACACAG

PI594774 TATGTGTGATTATGCTTATTAATGTGATTTTATTGAGTTTGTATAGTGTTATGTACACAG

Williams82 TATGTGTGATTATGCTTATTAATGTGATTTTATTGAGTTTGTATAGTGTTATGTACACAG

Blackhawk TATGTGTGATTATGCTTATTAATGTGATTTTATTGAGTTTGTATAGTGTTATGTACACAG

PI594891 ACACAAACACAACGCTGATGATTCAGCGGGATGCTGACATATGAATAATGGCGATCCAGA

PI594774 ACACAAACACAACGCAGATGATTCAGCGGGATGCTGACATATGAATAATGGCGATCCAGA

Williams82 ACACAAACACAACGCAGATGATTCAGCGGGATGCTGACATATGAATAATGGCGATCCAGA

Blackhawk ACACAAACACAACGCAGATGATTCAGCGGGATGCTGACATATGAATAATGGCGATCCAGA

PI594891 AAATTTATTTAGTGGAGAGAAAAAATAATTCATGATATTTTCATTGAAAATAATAGATTT

PI594774 AAATTTATTTAGTGGAGAGAAAAAATAATTCATGATATTTTCATTGAAAATAATAGATTT

Williams82 AAATTTATTTAGTGGAGAGAAAAAATAATTCATGATATTTTCATTGAAAATAATATATTT

Blackhawk AAATTTATTTAGTGGAGAGAAAAAATAATTCATGATATTTTCATTGAAAATAATAGATTT

PI594891 AATTAAGTTTTTCATTTCTGGAA

PI594774 AATTAAATTTTTCATTCCTGGAA

Williams82 AATTAAGTTTTTCATTCCTAGAA

Blackhawk AATTAAGTTTTTCATTTCTGGAA

1c. Glyma13g25340_protein alignment

Blackhawk MGWSNSELNSQILILSMLIFTASFCVGDTDPLDVAAINSLYVALGSPLLEGWKATGGDPC

PI594891 MGWSNSELNSQILILSMLIFTASFCVGDTDPLDVAAINSLYVALGSPLLEGWKATGGDPC

Williams82 MGWSNSELNSQILILSMLIFTASFCVGDTDPLDVAAINSLYVALGSPLLEGWKATGGDPC

PI594774 MGWSNSELNSQILILSMLIFTASFCVGDTDPLDVAAINSLYVALGSPLLEGWKATGGDPC

Blackhawk LEQWEGVSCVFSNITALRLGGMDLSGKLGTNLDFPSIIEMDLSNNQIGGTIPFTLPPTLR

PI594891 LEQWEGVSCVFSNITALRLGGMDLSGKLGTNLDFPSIIEMDLSNNQIGGTIPFTLPPTLR

Williams82 LEQWEGVSCVFSNITALRLGGMDLSGKLGTNLDFPSIIEMDLSNNQIGGTIPFTLPPTLR

PI594774 LEQWEGVSCVFSNITALRLGGMDLSGKLGTNLDFPSIIEMDLSNNQIGGTIPFTLPPTLR

Blackhawk NLSLSSNQLNGSIPDALSLLTQLSDLSLKDNHLNGQIPNAFLELTGLMNLDLSGNNLSGK

PI594891 NLSLSSNQLNGSIPDALSLLTQLSDLSLKDNHLNGQIPNAFLELTGLMNLDLSGNNLSGK

Williams82 NLSLSSNQLNGSIPDALSLLTQLSDLSLKDNHLNGQIPNAFLELTGLMNLDLSGNNLSGK

PI594774 NLSLS**T**NQLNGSIPDALSLLTQLSDLSLKDNHLNGQIPNAFLELTGLMNLDLSGNNLSGK

Blackhawk LPPSMGNLSSLITLNLQNNQLSGTLFVLQDLPLQDLNIENNIFSGPIPPELLSIPNFRKD

PI594891 LPPSMGNLSSLITLNLQNNQLSGTLFVLQDLPLQDLNIENNIFSGPIPPELLSIPNFRKD

Williams82 LPPSMGNLSSLITLNLQNNQLSGTLFVLQDLPLQDLNIENNIFSGPIPPELLSIPNFRKD

PI594774 LPPSMGNLSSLITLNLQNNQLSGTLFVLQDLPLQDLNIENNIFSGPIPPELLSIPNFRKD

Blackhawk GNPFNTTIIPSPPAAFPAPAAMAPSPEKSPWKMAHNPSDTIKAPIPAIAGRSFKTTKLVW

PI594891 GNPFNTTIIPSPPAAFPAPAAMAPSPEKSPWKMAHNPSDTIKAPIPAIAGRSFKTTKLVW

Williams82 GNPFNTTIIPSPPAAFPAPAAMAPSPEKSPWKMAHNPSDTIKAPIPAIAGRSFKTTKLVW

PI594774 GNPFNTTIIPSPPAAFPAPAAMAPSPEKSPWKMAHNPSDTIKAPIPAIAGRSFKTTKLVW

Blackhawk IVGAGFLIFIALGVCLLMLWCFKRRQENKKYKKHNTNMYTRSLHKRTCSNSPFEATNDEE

PI594891 IVGAGFLIFIALGVCLLMLWCFKRRQENKKYKKHNTNMYTRSLHKRTCSNSPFEATNDEE

Williams82 IVGAGFLIFIALGVCLLMLWCFKRRQENKKYKKHNTNMYTRSLHKRTCSNSPFEATNDEE

PI594774 IVGAGFLIFIALGVCLLMLWCFKRRQENKKYKKHNTNMYTRSLHKRTCSNSPFEATNDEE

Blackhawk KEWSSKLPPLQPAPPHHIPIIPGENLIINPAISTQAAERQIVTNSIKVYTVASLQQYTNS

PI594891 KEWSSKLPPLQPAPPHHIPIIPGENLIINPAISTQAAERQIVTNSIKVYTVASLQQYTNS

Williams82 KEWSSKLPPLQPAPPHHIPIIPGENLIINPAISTQAAERQIVTNSIKVYTVASLQQYTNS

PI594774 KEWSSKLPPLQPAPPHHIPIIPGENLIINPAISTQAAERQIVTNSIKVYTVASLQQYTNS

Blackhawk FSQENYIGEGMLGPVYRAELPDGKLLAVRKMNTTASMGQNHEQFLQLVFSISKIQHANIV

PI594891 FSQENYIGEGMLGPVYRAELPDGKLLAVRKMNTTASMGQNHEQFLQLVFSISKIQHANIV

Williams82 FSQENYIGEGMLGPVYRAELPDGKLLAVRKMNTTASMGQNHEQFLQLVFSISKIQHANIV

PI594774 FSQENYIGEGMLGPVYRAELPDGKLLAVRKMNTTASMGQNHEQFLQLVFSISKIQHANIV

Blackhawk KLMGYCAEYSQRLLVHEYCNNGTLHEALHTDDKLQIKLSWDDRIQVSLGAARALEYLHEH

PI594891 KLMGYCAEYSQRLLVHEYCNNGTLHEALHTDDKLQIKLSWDDRIQVSLGAARALEYLHEH

Williams82 KLMGYCAEYSQRLLVHEYCNNGTLHEALHTDDKLQIKLSWDDRIQVSLGAARALEYLHEH

PI594774 KLMGYCAEYSQRLLVHEYCNNGTLHEALHTDDKLQIKLSWDDRIQVSLGAARALEYLHEH

Blackhawk CQPPIVHRNFRSANILLNDKLEVLVSDCGLGSLLSSGSASQLLGRHLTANGYSAPEFEYG

PI594891 CQPPIVHRNFRSANILLNDKLEVLVSDCGLGSLLSSGSASQLLGRHLTANGYSAPEFEYG

Williams82 CQPPIVHRNFRSANILLNDKLEVLVSDCGLGSLLSSGSASQLLGRHLTANGYSAPEFEYG

PI594774 CQPPIVHRNFRSANILLNDKLEVLVSDCGLGSLLSSGSASQLLGRHLTANGYSAPEFEYG

Blackhawk SYTLQSDVFSFGVVMLELLTGRKSFDSSRPRVEQF**F**MRWAIPQLHDIDALSKMVDPSLNG

PI594891 SYTLQSDVFSFGVVMLELLTGRKSFDSSRPRVEQFLMRWAIPQLHDIDALSKMVDPSLNG

Williams82 SYTLQSDVFSFGVVMLELLTGRKSFDSSRPRVEQFLMRWAIPQLHDIDALSKMVDPSLNG

PI594774 SYTLQSDVFSFGVVMLELLTGRKSFDSSRPRVEQFLMRWAIPQLHDIDALSKMVDPSLNG

Blackhawk EYPKKSLSRFADIISSCIQNEPEFRPAMSEIVQDLLRMM

PI594891 EYPKKSLSRFADIISSCIQNEPEFRPAMSEIVQDLLRMM

Williams82 EYPKKSLSRFADIISSCIQNEPEFRPAMSEIVQDLLRMM

PI594774 EYPKKSLSRFADIISSCIQNEPEFRPAMSEIVQDLLRMM


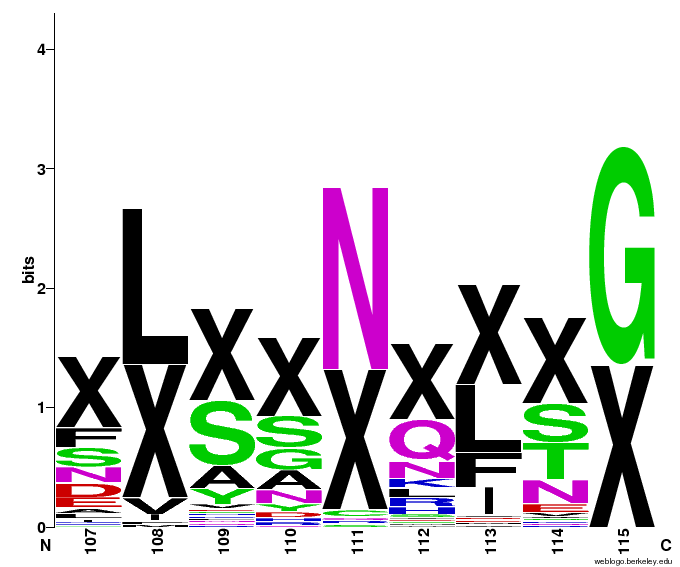


PI594891 ATGAAATTTTTTTCAGATACTTGAGTAAAAAATAAATGAATGTTAAGTAAAAATCTAAAA

Williams82 ATGAAATTTTTTTCAGATACTTGAGTGAAAAATAAATGAATGTTAAGTAAAAATCTAAAA

PI594774 ATGAAATTTTTTTCAGATACTTGAGTGAAAAATAAATGAATGTTAAGTAAAAATCTAAAA

Blackhawk ATGAAATTTTTTTCAGATACTTGAGTAAAAAATAAATGAATGTTAAGTAAAAATCTAAAA

PI594891 ATGATTTATATTTCAGGTATAGAAAACTTAATTAAGTAAAAAAAGTATGGTAAATTTTTA

Williams82 ATGATTTATATTTCAGGTATAGAAAACTTAATTAAGTAAAAAAAGTATGGTAAATTTTTA

PI594774 ATGATTTATATTTCAGGTATAGAAAACTTAATTAAGTAAAAAAAGTATGGTAAATTTTTA

Blackhawk ATGATTTATATTTCAGGTATAGAAAACTTAATTAAGTAAAAAAAGTATGGTAAATTTTTA

PI594891 CAAAATATGTAATTTTATGTAAAAGAAAAAATCTAACATGCTTAAGTTTATATAAAACTA

Williams82 CAAAATATGTAATTTTATGTAAAAGAAAAAATCTAACATGCTTAAGTTTATATAAAACTA

PI594774 CAAAATATGTAATTTTATGTAAAAGAAAAAATCTAACATGCTTAAGTTTATATAAAACTA

Blackhawk CAAAATATGTAATTTTATGTAAAAGAAAAAATCTAACATGCTTAAGTTTATATAAAACTA

PI594891 AAACATGTGAGACAAACTTTTTAAAATACTCTAAAAAGCACACCAGCAGGCCCTGAACTC

Williams82 AAACATGTGAGACAAACTTTTTAAAATACTCTAAAAAGCACATCAGCAGGCCCTGAACTC

PI594774 AAACATGTGAGACAAACTTTTTAAAATACTCTAAAAAGCACATCAGCAGGCCCTGAACTC

Blackhawk AAACATGTGAGACAAACTTTTTAAAATACTCTAAAAAGCACATCAGCAGGCCCTGAACTC

PI594891 GAAATATTTAGAGCACTTGGGGT-GGAGATACCTACTTGAATTAAGAATTGAAAAGGAAA

Williams82 GAAATATTTAGAGCACTTGGGGTTGGAGATACCTACTTGAATTAAGAATTGAAAAGGAAA

PI594774 GAAATATTTAGAGCACTTGGGGTTGGAGATACCTACTTGAATTAAGAATTGAAAAGGAAA

Blackhawk GAAATATTTAGAGCACTTGGGGTTGGAGATACCTACTTGAATTAAGAATTGAAAAGGAAA

PI594891 TAATTAATTAAGGTTTGGTTGCTCGTTAATAAAAATTGTATCCACGTGAAGCAATTATAT

Williams82 TAATTAATTAAGGTTTGGTTGCTCGTTAATAAAAATTGTATCCACGTGAAGCAATTATAT

PI594774 TAATTAATTAAGGTTTGGTTGCTCGTTAATAAAAATTGTATCCACGTGAAGCAATTATAT

Blackhawk TAATTAATTAAGGTTTGGTTGCTCGTTAATAAAAATTGTATCCACGTGAAGCAATTATAT

PI594891 GGACCCAAATAGATGGAGAAGAACAGGAATCCTTGAATGAGGTTGAGGACTTGACTACCC

Williams82 GGACCCAAATAGATGGAGAAGAACAGGAATCCTTGAATGAGGTTGAGGACTTGACTACCC

PI594774 GGACCCAAATAGATGGAGAAGAACAGGAATCCTTGAATGAGGTTGAGGACTTGACTACCC

Blackhawk GGACCCAAATAGATGGAGAAGAACAGGAATCCTTGAATGAGGTTGAGGACTTGACTACCC

PI594891 ACAACTACTTTAATTGGTCCTTATTTTTGCAGGTAAATATTGGTTTAATTTTTTGCTTGC

Williams82 ACAACTACTTTAATTGGTCCTTATTTTTGCAGGTAAATATTGGTTTAATTTTTTGCTTGC

PI594774 ACAACTACTTTAATTGGTCCTTATTTTTGCAGGTAAATATTGGTTTAATTTTTTGCTTGC

Blackhawk ACAACTACTTTAATTGGTCCTTATTTTTGCAGGTAAATATTGGTTTAATTTTTTGCTTGC

PI594891 TCCCATTTGCATTAACCATGTGCACATGCTATGTCGTAAAACACTAAACAGTGTTGTCAC

Williams82 TCCCATTTGCATTAACCATGTGCACATGCTATGTCGTAAAACACTAAACAGTGTTGTCAC

PI594774 TCCCATTTGCATTAACCATGTGCACATGCTATGTCGTAAAACACTAAACAGTGTTGTCAC

Blackhawk TCCCATTTGCATTAACCATGTGCACATGCTATGTCGTAAAACACTAAACAGTGTTGTCAC

PI594891 TTTCCAACTACAGATGCTTCTTATTTCTTATTTAAAAGGTTAAAGAAGGATCAGATGCTT

Williams82 TTTCCAACTACAGATGCTTCTTATTTCTTATTTAAAAGGTTAAAGAAGGATCAGATGCTT

PI594774 TTTCCAACTACAGATGCTTCTTATTTCTTATTTAAAAGGTTAAAGAAGGATCAGATGCTT

Blackhawk TTTCCAACTACAGATGCTTCCTATTTCTTATTTAAAAGGTTAAAGAAGGATCAGATGCTT

PI594891 GGCTCATATGGTAATTGTCAAGTCCTGTTTTATAGTTAACTAAAATTATAGTATACATTG

Williams82 GGTTCATATGGTAATTGTCAAGTCCTGTTTTATAGTTAACTAAAATTATAGTATACATTG

PI594774 GGCTCATATGGTAATTGTCAAGTCCTGTTTTATAGTTAACTAAAATTATAGTATACATTG

Blackhawk GGTTCATATGGTAATTGTCAAGTCCTGTTTTATAGTTAACTAAAATTATAGCATACATTG

PI594891 CAGAAGTTAAAGAAGGAAAATAAAGGGAATTGAAGAAGACGTTGAAAATGACCATAAAAA

Williams82 CAGAAGTTAAAGAAGGAAAATAAAGGGAATTGAAGAAGACGTTGAAAATGACCATAAAAA

PI594774 CGGAAGTTAAAGAAGGAAAATAAAGGGAATTGAAGAAGACGTTGAAAATGACCATAAAAA

Blackhawk CAGAAGTTAAAGAAGGAAAATAAAGGGAATTGAAGAAGACGTTGAAAATGACCATAAAAA

PI594891 ATATCATAAAACCGCAAATCCTGTTTCATGCATATCGTTTTTTTATCGAAAAATATTTAG

Williams82 ATATCATAAAACCGCAAATCCTGTTTCATGCATATTGTTTTTTTATCGAAAAATATTTAG

PI594774 ATATCATAAAACCGCAAATCCTGTTTCATGCATATTGTTTTTTTATCGAAAAATATTTAG

Blackhawk ATATCATAAAACCGCAAATCCTGTTTCATGCATATTGTTTTTTTATCGAAAAATATTTAG

PI594891 TTTGTTATGTTTGTTAGTCCTATACGTACTACGGTGTGATAATGGTTAATAGTAGTAATA

Williams82 TTTGTTATGTTTGTTAGTCCTATACGTACTACGGTGTGATAATGGTTAATAGTAGTAATA

PI594774 TTTGTTATGTTTGTTAGTCCTATACGTACTACGGTGTGATAATGGTTAATAGTAGTAATA

Blackhawk TTTGTTATGTTTGTTAGTCCTATACGTACTACGGTGTGATAATGGTTAATAGTAGTAATA

PI594891 TTCTCTTCACTTTTATGTAATGAGAAAAAATATAAGTAATTCTATTGGTTGTTAATTTGC

Williams82 TTCTCTTCACTTTTATGTAATGAGAAAAAATATAAGTAATTCTATTGGTTGTTAATTTGC

PI594774 TTCTCTTCACTTTTATGTAATGAGAAAAAATATAAGTAATTCTATTGGTTGTTAATTTGC

Blackhawk TTCTCTTCACTTTTATGTAATGAGAAAAAATATAAGTAATTCTATTGGTTGTTAATTTGC

PI594891 TTCGGTTCCTATATAACATTTACATTCAAAAATGTTTGCTTCGGTTCCTATATAAGATTT

Williams82 TTCGGTTCCTATATAACATTTACATTCAAAAATGTTTGCTTCGGTTCCTATATAAGATTT

PI594774 TTCGGTTCCTATATAACATTTACATTAAAAAATGTTTGCTTCGGTTCCTATATAAGATTT

Blackhawk TTCGGTTCCTATATAACATTTACATTCAAAAATGTTTGCTTCGGTTCCTATATAAGATTT

PI594891 ACATACAAAAATGTTTGTAATTTATAAGATTTACGTGAACCCAAATTACTATAAATCATG

Williams82 ACATACAAAAATGTTTGTAATTTATAAGATTTACGTGAACCCAAATTACTATAAATCATG

PI594774 ACATACAAAAATGTTTGTAATTTATAAGATTTACGTGAACCCAAATTACTATAAATCATG

Blackhawk ACATACAAAAATGTTTGTAATTTATAAGATTTACGTGAACCCAAATTACTATAAATCATG

PI594891 TAAACTACTAAAATTCTTATAGCGAGGGTACATTGTCTGATGGGGAAAATGAGTGACTAC

Williams82 TAAACTACTAAAATTCTTATAGCGAGGGTACATTGTCTGATGGGGAAAATGAGTGACTAC

PI594774 TAAACTACTAAAATTCTTATAGCGAGGGTACATTGTCTGATGGGGAAAATGAGTGACTAC

Blackhawk TAAACTACTAAAATTCTTATAGCGAGGGTACATTGTCTGATGGGGAAAATGAGTGACTAC

PI594891 AGATTAATTAACTATCCGCAGTAGACAGTAGGACTGCCCTGTTAGTACTTTGTCACTGAT

Williams82 AGATTAATTAACTATCCGCAGTAGACAGTAGGACTGCCCTGTTAGTACTTTGTCACTGAT

PI594774 AGATTAATTAACTATCCGCAGTAGACAGTAGGACTGCCCTGTTAGTACTTTGTCACTGAT

Blackhawk AGATTAATTAACTATCCGCAGTAGACAGTGGGACTGCCCTGTTAGTACTTTGTCACTGAT

PI594891 TTTGTGGCTTTGGTGTTGTAATTTTCATGGTAAGGGTACATCAATAGCAAATCTGGAATT

Williams82 TTTGTGGCTTTGGTGTTGTAATTTTCATGGTAAAGGTACATCAATAGCAAATCTGGAATT

PI594774 TTTGTGGCTTTGGTGTTGTAATTTTCATGGTAAAGGTACATCAATAGCAAATCTGGAATT

Blackhawk TTTGTGGCTTTGGTGTTGTAATTTTCATGGTAAAGGTACATCAATAGCAAATCTGGAATT

PI594891 GCCAAAACATTCTCTTTGCCCTTAAAAAAAATCACCAAGAGTACCCACCAGCATCATATG

Williams82 GCCAAAACATTCTCTTTGCCCTTAAAAAAAATCACCAAGAGTACCCACCAGCATCATATG

PI594774 GCCAAAACATTCTCTTTGCCCTTAAAAAAAATCACCAAGAGTACCCACCAGCATCATATG

Blackhawk GCCAAAACATTCTCTTTGCCCTTAAAAAAAATCACCAAGAGTACCCACCAGCATCATATG

PI594891 AGTAATAGTATGTCATTGGCAAATGAGAACCTATATATTTTTATTATTTTCAATAAAAAA

Williams82 AGTAATAGTATGTCATTGGCAAATGAGAACCTATATATTTTTATTATTTTCAATAAAAAA

PI594774 AGTAATAGTATGTCATTGGCAAATGAGAACCTATATATTTTTATTATTTTCAATAAAAAA

Blackhawk AGTAATAGTATGTCATTGGCAAATGAGAACCTATATATTTTTATTATTTTCAATTAAAAA

PI594891 AAATTGTATGCCACTTTTGATTCCCCCCACCCCCGGCATAATTTAATAGAACTTTTTCTC

Williams82 AA-TTGTATGCCACTTTTGATTCCCCCCACCCCCGGCATAATTTAATAGAACTTTTTCTC

PI594774 AAATTGTATGCCACTTTTGATTCCCCCCACCCCCGGCATAATTTAATAGAACTTTTTCTC

Blackhawk AA-TTGTATGCCACTTTTGATTCCCCCCACCCCCGGCATAATTTAATAGAACTTTTTCTC

PI594891 CTTTTTAACGAGTGTGTTGTGTTTTTTGGTAATTTATAATTATGTTATTGATGCGAAGTA

Williams82 CTTTTTAACGAGTGTGTTGTGTTTTTTGGTAATTTATAATTATGTTATTGATGCGAAGTA

PI594774 CTTTTTAACGAGTGTGTTGTGTTTTTTGGTAATTTATAATTATGTTATTGATGCGAAGTA

Blackhawk CTTTTTAACGAGTGTGTTGTGTTTTTTGGTAATTTATAATTATGTTATTGATGCGAAGTA

PI594891 TATTTTTTATTATTTATCTATCTGTGTTATTAATAATTAATTTTTATTGAATGATTTATT

Williams82 TATTTTTTATTATTTATCTATCTGTGTTATTAATAATTAATTTTTATTGAATGATTTATT

PI594774 TATTTTTTATTATTTATCTATCTGTGTTATTAATAATTAATTTTTATTGAATGATTTATT

Blackhawk TATTTTTTATTATTTATCTATCTGTGTTATTAATAATTAATTTTTATTGAATGATTTATT

PI594891 TGATTACTTTTAGTAAAAAAAAATTATTCATGTTTCATTTTTCTATTAATAATGTTTGAA

Williams82 TGATTACTTTTAGTAAAAAAAAATTATTCATGTTTCATTTTTCTATTAATAATGTTTGAA

PI594774 TGATTACTTTTAGTAAAAAAAAATTATTCATGTTTCATTTTTCTATTAATAATGTTTGAA

Blackhawk TGATTACTTTTAGTAAAAAAAAATTATTCATGTTTCATTTTTCTATTAATAATGTTTGAA

PI594891 CTTAAGATCATGATTAAGAGATTCCAATTATTCAATTCTACTTAGATTAACAATGTATTA

Williams82 CTTAAGATCATGATGAAGAGAATCCAATTATTCAATTCTACTTAGATTAACAATGTATTA

PI594774 CTTAAGATCATGATTAAGAGATTCCAATTATTCAATTCTACTTAGATTATAAATGTATTA

Blackhawk CTTA------TGATTAAGAGATTCCAATTATTCAATTCTACTTAGATTAACAATGTATTA

PI594891 TTTT--AGGATTATTGTGTTTTATAGATAACTAAAATTATAGTATACATTGCAGATGATA

Williams82 TTTT--AGGATTATTGTGTTTTATAGATAACTAAAATTATAGTATACATTGCAGATGATA

PI594774 TTTT--AGGATTATTGTGTTTTATAGATAACTAAAATTATAGTATACATTGCAGATGATA

Blackhawk TTTTTTAGGATTATTGTGTTTTATAGATAACTAAAATTATAGTATACATTGCAGATGATA

PI594891 AATATGTATTTTTCTATAGTTAAAATTTATCATAAATAAGTTCTTTTTATAAATTATATT

Williams82 AATATGTATTTTTCTATAGTTAAAATTTATCATAAATAAGTTCTTTTTATAAATTATATT

PI594774 AATATGTATTTTTCTATAGTTAAAATTTATCATAAATAAGTTCTTTTTATAAATTATATT

Blackhawk AATATGTATTTTTCTATAGTTAAAATTTATCATAAATAAGTTCTTTTTATAAATTATATT

PI594891 ATAGATCAAATTCATAATAAATAAATATCTTTGAATATAAAATATATTTTAAATTTACAG

Williams82 ATAGATCAAATTCATAATAAATAAATATCTTTGAATATAAAATATATTTTAAATTTACAG

PI594774 ATAGATCAAATTCATAATAAATAAATATCTTTGAATATAAAATATATTTTAAATTTACAG

Blackhawk ATAGATCAAATTCATAATAAATAAATATCTTTGAATATAAAATATATTTTAAATTTACAG

PI594891 TGAACAATTTGAATAATGATATAAGATTATTTTTAATTACGTTGATAATATTTTTTTAAA

Williams82 TGAACAATTTGAATAATGATATAAGATTATTTTTAATTACGTTGATAATATTTTTTTAAA

PI594774 TGAACAATTTGAATAATGATATAAGATTATTTTTAATTACGTTGATAATATTTTTTTAAA

Blackhawk TGAACAATTTGAATAATGATATAAGATTATTCTTAATTACGTTGATAATATTTTTTTAAA

PI594891 AATATTGAATTTTAATTTAGAAATAGAAATAAAAATGATTACTAAATTAAAATAGACAGA

Williams82 AATATTGAATTTTAATTTAGAAATAGAAATAAAAATGATTACTAAATTAAAATAGACAGA

PI594774 AATATTGAATTTTAATTTAGAAATAGAAATAAAAATGATTACTAAATTAAAATAGACAGA

Blackhawk AATATTGAATTTTGATTTAGAAATAGAAATAAAAATGATTACTAAATTAAAATAGACAGA

PI594891 TTGTTAAGAAAATCAGATTTGCAAATAAAGATTAAAGAAAAGAAGTTTGGGGTGTTGAGA

Williams82 TTGTTAAGAAAATCAGATTTGCAAATAAAGATTAAAGAAAAGAAGTTTGGGGTGTTGAGA

PI594774 TTGTTAAGAAAATCAGATTTGCAAATAAAGATTAAAGAAAAGAAGTTTGGGGTGTTGAGA

Blackhawk TTGTTAAGAAAATCAGATTTGCAAATAAAGATTAAAGAAAAGAAGTTTGGGGTGTTGAGA

PI594891 AAGAATTTTTATTTTTTGGGTTTTTGCAAGAATAATACTAGAAAAGTGCATCTAAAAGAG

Williams82 AAGAATTTTTATTTTTTGGGTTTTTGCAAGAATAATACTAGAAAAGTGCATCTAAAAGAG

PI594774 AAGAATTTTTATTTTTTGGGTTTTTGCAAGAATAATACTAGAAAAGTGCATCTAAAAGAG

Blackhawk AAGAATTTTTATTTTTTGGGTTTTTGCAAGAATAATACTAGAAAAGTGCATCTAAAAGAG

PI594891 AAAATAAATATTAAAAATATCAAACTTAGTGAAGAAACTTCAAATTGTGTATTCTAATTT

Williams82 AAAATAAATATTAAAAATATCAAACTTAGTGAAGAAACTTCAAATTGTGTATTCTAATTT

PI594774 AAAATAAATATTAAAAATATCAAACTTAGTGAAGAAACTTCAAATTGTGTATTCTAATTT

Blackhawk AAAATAAATATTAAAAATATCAAACTTAGTGAAGAAACTTCAAATTGTGTATTCTAATTT

PI594891 GCAATATATTAAAATACTTCAAAGTTCAAATAATTCAAAGGTTCTCTTGGGGAGAAATTG

Williams82 GCAATATATTAAAATATTTCAAAGTTCAAATAATTCAAAGGTTCTCTTGGGGAGAAATTG

PI594774 GCAATATATTAAAATATTTCAAAGTTCAAATAATTCAAAGGTTCTCTTGGGGAGAAATTG

Blackhawk GCAATATATTAAAATACTTCAAAGTTCAAATAATTCAAAGGTTCTCTTGGGGAGAAATTG

PI594891 GTTGAAACAAAAATCACATGCTACTCATTACGTGTTCATAAGCAATTTATGAGCATTTGA

Williams82 GTTGAAACAAAAATCACATGCTACTCATTACGTGTTCATAAGCAATTTATGAGCATTTGA

PI594774 GTTGAAACAAAAATCACATGCTACTCATTACGTGTTCATAAGCAATTTATGAGCATTTGA

Blackhawk GTTGAAACAAAAATCACATGCTACTCATTACGTGTTCATAAGCAATTTATGAGCATTTGA

PI594891 GCACCTACGGTTGGTGCGAACTGGAAGTTGATGTGCAATTGTGCATCTTGAAACTATTTA

Williams82 GCACCTACGGTTGGTGCGAACTGGAAGTTGATGTGCAATTGTGCATCTTGAAACTATTTA

PI594774 GCACCTACGGTTGGTGCGAACTGGAAGTTGATGTGCAATTGTGCATCTTGAAACTATTTA

Blackhawk GCACCTACGGTTGGTGCGAACTGGAAGTTGATGTGCAATTGTGCATCTTGAAACTATTTA

PI594891 ACTGATATATGATTAGTGCACGACATACATACAATAGAACAATAATGAAATTTAGGTTAC

Williams82 ACTGATATATGATTAGTGCACGACATACATACAATAGAACAATAATGAAATTTAGGTTAC

PI594774 ACTGATATATGATTAGTGCACGACATACATACAATAGAACAATAATGAAATTTAGGTTAC

Blackhawk ACTGATATATGATTTGTGCACGACATACATAAAATAGAACAATAATGAAATTTAGGTTAC

PI594891 ATTTATTTGCTAAAAAATGCGGTTTAACCTAATAACGAATTGAACTTAGTTTTAAAGTTG

Williams82 ATTTATTTGCTAAAAAATGCGGTTTAACCTAATAACGAATTGAACTTAGTTTTAAAGTTG

PI594774 ATTTATTTGCTAAAAAATGCGGTTTAACCTAATAACGAATTGAACTTAGTTTTAAAGTTG

Blackhawk ATTTATTTGCTAAAAAATGCGGTTTAACCTAATAACGAATTGAACTTAGTTTTAAAGTTG

PI594891 GTATAATTCCTATCTAATTTTTAGTTTTTACCAGTACTCCTATCTTCATGTTTTTCATTT

Williams82 GTATAATTCCTATCTAATTTTTAGTTTTTACCAGTACTCCTATCTTCATGTTTTTCATTT

PI594774 GTATAATTCCTATCTAATTTTTAGTTTTTACCAGTACTCCTATCTTCATGTTTTTCATTT

Blackhawk GTATAATTCCTATCTAATTTTTAGTTTTTACCAGTACTCCTATCTTCATGTTTTGCATTT

PI594891 TTGTAATTATTTAAAAAGTAAATATTAAAAAAAAAATAGAACCTAATTCTTAATTAGTTT

Williams82 TTGTAATTATTTAAAAAGTAAATATTAAAAAAAA--TAGAACCTAATTCTTAATTAGTTT

PI594774 TTGTAATTATTTAAAAAGTAAATATTAAAAAAAAA-TAGAACCTAATTCTTAATTAGTTT

Blackhawk TTGTAATTATTTAAAAAGTAAATATTAAAAAAAAA-TAGAACCTAATTCTTAATTAGTTT

PI594891 GTATGTTTCTGACTTGAATTTGTGTTTCTGTGGTAACTTTGGACAAGGGTTACGTTTTCT

Williams82 GTATGTTTCTGACTTGAATTTGTGTTTCTGTGGTAACTTTGGACAAGGGTTACGTTTTCT

PI594774 GTATGTTTCTGACTTGAATTTGTGTTTCTGTGGTAACTTTGGACAAGGGTTACGTTTTCT

Blackhawk GTATGTTTCTGACTTGAATTTGTGTTTCTGTGGTAACTTTGGACAACGGTTA-----TCT

PI594891 AGGTTGAATTTGAGTTTAAGAGGTAAAGAAAAGTAATGAGATTTTGAGGTTTAGTTATTA

Williams82 AGGTTGAATTTGAGTTTAAGAGGTAAAGAAAAGTAATGAGATTTTGAGGTTTAGTTATTA

PI594774 AGGTTGAATTTGAGTTTAAGAGGTAAAGAAAAGTGATGAGATTTTGAGGTTTAGTTATTA

Blackhawk AGGTTGAATTTGAGTTTAAGAGGTAAAGAAAAGTAATGAGATTTTGAGGTTTAGTTATTA

PI594891 TTTTTTGTATCCTAATTGTGATTCATTTTGATGTATTGTTAGTAGTTAATTAGCTATGCA

Williams82 TTTTTTGTATCCTAATTCTGATTCATTTTGATGTATTGTTAGTAGTTAATTAGCTATGCA

PI594774 TTTTTTGTATCCTAATTGTGATTCATTTTGATGTATTGTTAGTAGTTAATTAGCTATGCA

Blackhawk TTTTTTGTATCCTAATTGTGATTCATTTTGATGTATTGTTAGTAGTTAATTAGCTATGCC

***************** *****************************************.

PI594891 AGAGTTTTCTTTTGCAATGATAAAAAAAAAA-GTTCATTGTTAATTAGCAAAATTCATTG

Williams82 AGAGTTTTCTTTTGCAATGATAAAAAAAAAA-GTTCATTGTTAATTAGCAAAATTCATTG

PI594774 AGAGTTTTCTTTTGCAATGATAAAAAAAAAAAGTTCATTGTTAATTAGCAAAATTCATTG

Blackhawk AGAGTTTTCTTTTGCAATGATAAGAAAAAAACGTTCATTGTTAATTAGCAAAATTCATTG

PI594891 TTTTGTAATTTGCAATTATTTAAAAAAAATGGGCTGAGTGAAGTCATAATGTTTATGTTT

Williams82 TTTTGTAATTTGCAATTATTTAAAAAAAATGGGCTGAGTGAAGTCATAATGTTTATGTTT

PI594774 TTTTGTAATTTGCAATTATTTAAAAAAAACGGGCTGAGTGAAGTCATAATGTTTATGTTT

Blackhawk TTTTGTAATTTGCAATGATTTAAAAAAA-TGGGCTGAGTGAAGTCATGATGTTTATGTTT

PI594891 TGTTAGGATTCTTGGCGCCAAAGAATTTAAATTAGATACTTAAAAAGAGAGTTATTTATT

Williams82 TGTTAGGATTCTTGGCGCCAAAGAATTTAAATTAGATACTTAAAAAGAGAGTTATTTATT

PI594774 TGTTAGGATTCTTGGCGCCAAAGAATTTAAATTAGATACTTAAAAAGAGAGTTATTTATT

Blackhawk TGTTAGGATTCTTGGCGCCAAAGAATTTAAATTAGATACTTAAAAAGAGAGTTATTTATT

PI594891 GAATCCAATTTTTAGTTGATTATCACTCAACTTAATCACGCGTGAATCTCTCTCTCATAA

Williams82 GAATCCAATTTTTAGTTGATTATCACTCAACTTAATCACGCGTGAATCTCTCTCTCATAA

PI594774 GAATCCAATTTTTAGTTGATTATCACTCAACTTAATCACGCGTGAATCTCTCTCTCATAA

Blackhawk GAATCCAATTTTTAGTTGATTATCACTCAACTTAATCACGCGTGAATCTCTCTCTCATAA

PI594891 GTAGGAGGCAGAAACATGTATGCCACGTGAGAGTTTAGTTTGAGTGCATGGCATTTATGG

Williams82 GTAGGAGGCAGAAACATGTATGCCACGTGAGAGTTTAGTTTGAGTGCATGGCATTTATGG

PI594774 GTAGGAGGCAGAAACATGTATGCCACGTGAGAGTTTAGTTTGAGTGCATGGCATTTATGG

Blackhawk GTAGGAGGCAGAAACATGTATGCCACGTGAGAGTTTAGTTTGAGTGCATGGCATTTATGG

PI594891 CTCACATTATTATTTGTTGTTAAGAGCGATGAAAAATTAATATTACATGGAGATATAATT

Williams82 CTCACATTATTATTTGTTGTTAAGAGCGATGAAAAATTAATATTACATGGAGATATAATT

PI594774 CTCACATTATTATTTGTTGTTAAGAGCGATGAAAAATTAATATTACATGGAGATATAATT

Blackhawk CTCACATTATTATTTGTTGTTAAGAGCGATGAAAAATTAATATTACATGGAGATATAATT

PI594891 AATATTAATCTTTAAAAAAAATATTTACACAAAAACTTTTTAGGATTAGAAATAAGAATT

Williams82 AATATTAATCTTTAAAAAAAATATTTACACAAAAACTTTTTAGGATTAGAAATAAGAATT

PI594774 AATATTAATCTTTAAAAAAAATATTTACACAAAAACTTTTTAGGATTAGAAATAAGAATT

Blackhawk AATATTAATCTTTAAAAAAAATATTTACACAAAAACTTTTTAGGATTAGAAATAAGAATT

PI594891 AACTAATAAATATATAATTATTAATTTGAATTTAAATTCTGGCAAATTTTATTCTATTTA

Williams82 AACTAATAAATATATAATTATTAATTTGAATTTAAATTCTGGCAAATTTTATTCTATTTA

PI594774 AACTAATAAATATATAATTATTAATTTGAATTTAAATTTTGGCAAATTTTATTCTATTTA

Blackhawk AACTAATAAATATATAATTATTAATTTGAATTTAAATTCTGGCAAATTTTATTCTATTTA

PI594891 TTTTATATATAAACAT---TATTATTATTCCAAAAAATACATAGCTCTATATCCATGTCC

Williams82 TTTTATATATAAACAT---TATTATTATTCCAAAAAATACATAGCTCTATATCCATGTCC

PI594774 TTTTATATATAAACATTATTATTATTATTCCAAAAAATACATAGCTCTATATCCATGTCC

Blackhawk TTTTATATATAAACAT---TATTATTATTCCAAAAAATACATAGCTCTATATCCATGTCC

PI594891 TTTTAACTAACTGTTTTGAATGAGGTTGATGTATGCCCATCAAATCAACTTATTCGATGA

Williams82 TTTTAACTAACTGTTTTGAATGAGGTTGATGTATGCCCATCAAATCAACTTATTCGATGA

PI594774 TTTTAACTAACTGTTTTGAATGAGGTTGATGTATGCCCATCAAATCAACTTATTCGATGA

Blackhawk TTTTAACTAACTGTTTTGAATGAGGTTGATGTATGCCCATCAAATCAACTTATTCGATGA

PI594891 CAACGTAACGTAGAGTGTGTACATAGCTATCCAATTTCTGCCCAAATAATGAAAAATTAA

Williams82 CAACGTAACGTAGAGTGTGTACATAGCTATCCAATTTCTGCCCAAATAATGAAAAATTAA

PI594774 CAAGGTAACGTAGAGTGTGTACGTAGCTATCCAATTTCTGCCCAAATAATGAAAAATTAA

Blackhawk CAACGTAACGTAGAGTGTGTACATAGCTATCCAATTTCTGCCCAAATAATGAAAATTTAA

PI594891 TGCTGAAGAAATGGAGCTATATATGCTATACTACTAAGCATTAAGGAATGTCCAGTCCCA

Williams82 TGCTGAAGAAATGGAGCTATATATGCTATACTACTAAGCATTAAGGAATGTCCAGTCCCA

PI594774 TGCTGAAGAAATGGAGCTATATATGCTATACTACTAAGCATTAAGGAATGTCCAGTCCCA

Blackhawk TGCTGAAGAAATGGAGCTATATATGCTATACTACTAAGCATTAAGGAATGTCCAGTCCCA

PI594891 ATTGTCTTTTTTATTTTTTACAGAAAAAAAATGTGTAAGTTTCATACTTTATATTCCATC

Williams82 ATTGTCTTTTTTATTTTTTACAGAAAAAAAATGTGTAAGTTTCATACTTTATATTCCATC

PI594774 ATTGTCTTTTTTATTTTTTACAGAAAAAAAATGT--AAGTTTCATACTTTATATTCTATC

Blackhawk ATTGTCTTTTTTATTTTTTACAGAAAAAAAATGT--AAGTTTCATACTTTATATTCTATC

PI594891 AAATGTTCCCTTGAAAATTCTTTTTATTCGCTGCA-ATATTCCTGATCAGTGTATATTAA

Williams82 AAATGTTCCCTTGAAAATTCTTTTTATTCGCTGCA-ATATTCCTGATCAGTGTATATTAA

PI594774 AAATGTTCCCTTGAAAATTCTTTTTATTCGCTGCATATATTCCTGATCAGTGTATATTAA

Blackhawk AAATGTTCCCTTGAAAATTCTTTTTATTCGCTGCATATATTCCTGATCAGTGTATATTAA

PI594891 ATTGTGACTTAAGTTAGATTAGATTTTCAACAGTACCCCTCAG------TAAAAAAAAAT

Williams82 ATTGTGACTTAAGTTAGATTAGATTTTCAACAGTACCCCTCAG------TAAAAAAAAAT

PI594774 ATTGTGACTTCAGTTAGATTAGATTTTCAACAGTACCCCTCAG-TAAAATAAAAAAAAAT

Blackhawk ATTGTGACTTCAGTTAGATTAGATTTTCAACAGTACCCCTCAGTAAAATAAAAAAAAAAT

PI594891 CATCAGTACCCCAACTCAATTCAAACTATCTTAATTTTATTATAATTTTTTATTTTATTT

Williams82 CATCAGTACCCCAACTCAATTCAAACTATCTTAATTTTATTATAATTTTTTATTTTATTT

PI594774 CAACAGTATCCCAACTCAATCCAAACTATCTTAATTTTATTATAATTTTTTATTTTATTT

Blackhawk CAACAGTATCCCAACTCAATCCAAACTATCTTAATTTTATTATAATTTTTTATTTTATTT

PI594891 TATTTTACTTAATATTTTAAAATTTCAATAAGAATATATAAATCTATGTGCAATAAGAAA

Williams82 TATTTTACTTAATATTTTAAAATTTCAATAAGAATATATAAATCTATGTGCAATAAGAAA

PI594774 TATTTTACTTAATATTTTAAAAATTCAATAAGAATATATAAATCTATGTGCAATAAGAAA

Blackhawk TATTTTACTTAATATTTTAAAAATTCAATAAGAATATATAAATCTATGTGCAATAAGAAA

PI594891 ATTTCTCTTTTTTTTTA-------TATTTTATAAAAATTTCTTTTGAAAATGTAATTATA

Williams82 ATTTCTCTTTTTTTTTA-------TATTTTATAAAAATTTATTTTGAAAATGTAATTATA

PI594774 ATTTCTCTTTTTACTTT-T-TTTTATATTTATAAACATTTCTTTTAAAAATTTAATTATA

Blackhawk ATTTCTCTTTTTACTTT-T-TTTTATATTTATAAACATTTCTTTTAAAAATTTAATTATA

PI594891 TGTATACTACGAAATTTAATCTTTTTATTTAACATTATTATGATTTTTATTAATATTTTC

Williams82 TGTATACTACGAAATTTAATCTTTTTATTTAACATTATTATGATTTTTATTAATATTTTC

PI594774 TGTATACTACAAAATTTAATCTTTTTATTTAACATTATTATGATTTTTATTAATATTTT-

Blackhawk TGTATACTACAAAATTTAATCTTTTTATTTAACATTATTATGATTTTTATTAATATTTT-

PI594891 TTTTAAAAAATTAAATCGTAAAATATGTATTAACCCGATGACTCAATTCTATCCAAACTA

Williams82 TTTTAAAAAATTAAATCGTAAAATATGTATTAACCCGATGACTCAATTCTATCCAAACTA

PI594774 TTTAAAAAAAATAAATCGTAAAATATGTATTAACCCGATGACTCAATTCCATCCAAATTA

Blackhawk TTTTAAAAAAATAAATCGTAAAATATGTATTAACCCGATGACTCAATTCCATCCAAATTA

PI594891 AATTCAATTCAAATCACTTAGTTTAAGTCAAATTAATTAAAATAATTTTGAAAAACCCAA

Williams82 AATTCAATTCAAATCACTTAGTTTAAGTCAAATTAATTAAAATAATTTTGAAAAACCCAA

PI594774 AATTCAATTCAAATCACTTAGTTCAAGTCAAATTAATTAAAATAATTTTGAAAAACACAA

Blackhawk AATTCAATTCAAATCACTTAGTTCAAGTCAAATTAATTAAAATAATTTTGAAAAACACAA

PI594891 ATAAATCCAACCTATAAATTTCATCCATTTATATTTTCTTTATCAAACCTACCGCATTCT

Williams82 ATAAATCCAACCTATAAATTTCATCCATTTATATTTTCTTTATCAAACCTACCGCATTCT

PI594774 ATAAATCCAACCTATAAATTTCATCAATTTATATTTTCTTTATCAAACCTACCGCATTTC

Blackhawk ATAAATCCAACCTATAAATTTCATCAATTTATATTTTCTTTATCAAACCTACCGCATTCT

PI594891 AACCTATAAACACCCTTATTAATTACACTAAATAATGTGATATCATTCCATAATTTGGT

Williams82 AACCTATAAACACCCTTATTAATTACACTAAATAATGTGATATCATTCCATAATTTGGT

PI594774 TACCTATAAACACCCTTATTAATTACACTAAATAATGTGATATCACTCCATAATTTGGT

Blackhawk AACCTATAAACACCCTTATTAATTACACTAAATAATGTGATATCATTCCATAATTTGGT

PI594891 ACAACTTCAAAAGGTTTTACATTGCAAAGTATTTTGCAAACATTGTTTGGTGTAAATTTG

Williams82 ACAACTTCAAAAGGTTTTACATTGCAAAGTATTTTGCAAACATTGTTTGGTGTAAATTTG

PI594774 ACAACTTCAAAAGGTTTTACATTGTAAAGTATTTTGCAAACATTGTTTGGTGTAAATTTG

Blackhawk ACAACTTCAAAAGGTTTTACATTGTAAAGTATTTTGCAAACATTGTTTGGTGTAAATTTG

PI594891 CATCTCATAATTTTAAGATTACAACATTGGATACATAAGGAAATTTTAATAACTATTTAG

Williams82 CATCTCATAATTTTAAGATTACAACATTGGATACATAAGGAAATTTTAATAACTATTTAG

PI594774 CATCTCATAATTTTACGATTACAACATTGGATACATAAGGAAATTTTAATAACTATTTAG

Blackhawk CATCTCATAATTTTACGATTACAACATTGGATACATAAGGAAATTTTAATAACTATTTAG

PI594891 CGGCATTCATTACTCTTTATCATGTCATTATTATTTTATTTCTTAAAATTAATATGATAC

Williams82 CGGCATTCATTACTCTTTATCATGTCATTATTATTTTATTTCTTAAAATTAATATGATAC

PI594774 CGGCATTCATTACTCTTTATCATGTCATTATTATTTTATTTCTTAAAATTAATATGATAC

Blackhawk CGGCATTCATTACTCTTTATCATGTCATTATTATTTTATTTCTTAAAATTAATATGATAC

PI594891 ATCTATTCGTTAAAAGAAGTCATATATACTCATTAAATATATTTAACCAATAATATTAAT

Williams82 ATCTATTCGTTAAAAGAAGTCATATATACTCATTAAATATATTTAACCAATAATATTAAT

PI594774 ATGTATTAGTTAAAAGAAGTCATATATACTCATTAAATATATTTAACCAATAATATTAAT

Blackhawk ATGTATTAGTTAAAAGAAGTCATATATACTCATTAAATATATTTAACCAATAATATTAAT

PI594891 TCAAAACGATATAAGTATTCCTAACAAAAAAATTAATTTCAAATTATAAAGTAAAAAAAT

Williams82 TCAAAACGATATAAGTATTCCTAACAAAAAAATTAATTTCAAATTATAAAGTAAAAAAAT

PI594774 TCAAAACGATATAAGTATTCCTAACAAAAAAATTGATTTCAAATTATAAAGTAAAAAAAT

Blackhawk TCAAAACGATATAAGTATTCCTAACAAAAAAATTGATTTCAAATTATAAAGTAAAAAAAT

PI594891 AATATTACACACACAATATTCAACGCTTAATTCAAACAAAATCTCTTTAAAAAAAATCAG

Williams82 AATATTACACACACAATATTCAACGCTTAATTCAAACAAAATCTCTTTAAAAAAAATCAG

PI594774 AATATTACACACGCAATATTCAACGCTTAATTCAAACAAAATCTCTTAAAAAAAAATCAG

Blackhawk AATATTACACACGCAATATTCAACGCTTAATTCAAACAAAATCTCTTTAAAAAAAATCAG

PI594891 ACTTCAT-AGCTCAGTAAAATAATATGAAATCCACACTTTCATATTCTATCTTAATTTAA

Williams82 ACTTCAT-AGCTCAGTAAAATAATATGAAATCCACACTTTCATATTCTATCTTAATTTAA

PI594774 ACTTCAT-AGCTCAGTAAAATAATATGAAATCCACACTTTTATATTCTATCTTAATTTAA

Blackhawk ACTTCATTAGCTCAGTAAAATAATATGAAATCCACACTTTTATATTCTATCTTAATTTAA

PI594891 TACTTTCAATTCAATTCACTTTATTTTTATTCTTTCCTCTCCACCAAACCTCCCTTAGAT

Williams82 TACTTTCAATTCAATTCACTTTATTTTTATTCTTTCCTCTCCACCAAACCTACCTTAGAT

PI594774 TACTTTCAATTCAATTCACTTTATTTTTATTCTTTCCTCTCCACCTAACCTACCTTAGAT

Blackhawk TACTTTCAATTCAATTCACTTTATTTTTATTCTTTCCTCTCCACCTAACCTACCTTAGAT

PI594891 AAATTGGCAACGAAAATGGCTTACTGAAGGGCCTTAAGAAAAAATTTGCGTACTAAAGGG

Williams82 AAATTGGCAACGACAATTGCTTACTGAAGGGCCTTAAGAAAAAATTTGCGTACTAAAGGG

PI594774 AAATTGGCAACGACAATTGCTTACTGAAGGGCCTTAAGAAAAAATTTGCGTACTAAAGGG

Blackhawk AAATTGGCAACGACAATTGCTTACTGAAGGGCCTTAAGAAAAAATTTGCGTACTAAAGGG

PI594891 TATTTTGGTCTGAGGAGTAAACATGGGTATGAGTCTACGTGTTCCCCACAGAAAATAAAG

Williams82 TATTTTGGTCTGAGGAGTAAACATGGGTATGAGTCTACGTGTTCCCCACAGAAAATAAAG

PI594774 TATTTTGGTCTGAGGAGTAAACATTGGTATGAGTCCACGTATTCCCCACAGAAAATAAAG

Blackhawk TATTTTGGTCTGAGGAGTAAACATTGGTATGAGTCCACGTATTCCCCACAGAAAATAAAG

PI594891 AAGTTGACAACAATATTGGATAATATTAAATATTAAATATTAAATAATAATAAAGAAGAT

Williams82 AAGTTGACAACAATATTGGATAATATTAAATATTAAATATTAAATAATAATAAAGAAGAT

PI594774 AAGTTGACAACAATATTGGATAATATTAAATATTAAATATTAAATAATAATAAAGAAGAT

Blackhawk AAGTTGACAACAATATTGGATAATATTAAATATTAAATATTAAATAATAATAAAGAAGAT

PI594891 TATTTCTGACTGTTGAATGATTGTTCATTTGTTCTTCTTCTTTCTTGGTTGGTTTGTTAA

Williams82 TATTTCTGACTGTTGAATGATTGTTCATTTGTTCTTCTTCTTTCTTGGTTGGTTTGTTAA

PI594774 TATTTCTGACTGTTGAATGATTGTTCATTTGTTCTTCTTCTTTCTTGGTTGGTTTGTTAA

Blackhawk TATTTCTGACTGTTGAATGATTGTTCATTTGTTCTTCTTCTTTCTTGGTTGGTTTGTTAA

PI594891 GAGCAGTTCAGTTTCCACTTCCAAAACTGAAACACAAATTCTCAGTTTGGTTCTGAAGTG

Williams82 GAGCAGTTCAGTTTCCACTTCCAAAACTGAAACACAAATTCTCAGTTTGGTTCTGAAGTG

PI594774 GAGCAGTTCAGTTTCCACTTCCAAAACTGAAACACAAATTCTCAGTTTGGTTCTGAAGTG

Blackhawk GAGCAGTTCAGTTTCCACTTCCAAAACTGAAACACAAATTCTCAGTTTGGTTCTGAAGTG

PI594891 AAGAAGCTGACCCAATTCCTCATTTGCTTCATTTGTGTGGAAAGGAACTTCATTTCT---

Williams82 AAGAAGCTGACCCAATTCCTCATTTGCTTCATTTGTGTGGAAAGGAACTTCATTTCTGCT

PI594774 AAGAAGCTGACCCAATTCCTCATTTGCTTCATTTGTGTGGAAAGGAACTTCATTTCTGCT

Blackhawk AAGAAGCTGACCCAATTCCTCATTTGCTTCATTTGTGTGGAAAGGAACTTCATTTCT---

PI594891 --ACAAGCTGCCTTTGCTGTTTCCCAACAACACTA-TGGCAAAGACAGGATACAAACTAC

Williams82 CCACAAGCTGCCTTTGCTGTTTCCCAACAACACTAATGGCAAAGACAGGATACAAACTAC

PI594774 CCACAAGCTGCCTTTGCTGTTTCCCAACAACACTAATGGCAAAGACAGGATACAAACTAC

Blackhawk --ACAAGCTGCCTTTGCTGTTTCCCAACAACACTA-TGGCAAAGACAGGATACAAACTAC

PI594891 GTATCCTTTTTTATATATATAAGTTTTGTGCCTCAGAAAATGCTTTCTGTTGCAATGTGG

Williams82 GTATCCTTTTTTATATATATAAGTTTTGTGCCTCAGAAAATGCTTTCTGTTGCAATGTGG

PI594774 GTATCCTTTTTTATATATATAAGTTTTGTGCCTCAGAAAATGCTTTCTGTTGCAATGTGG

Blackhawk GTATCCTTTTTTATATATATAAGTTTTGTGCCTCAGAAAATGCTTTCTGTTGCAATGTGG

PI594891 GTGAGTTGTGTCCTTGAAACTCTTGTTTTTTTTT--CTTTCTTATTTTATGGTTCGTTGG

Williams82 GTGAGTTGTGTCCTTGAAACTCTTGTTTTTTTTTTTCTTTCTTATTTTATGGTTCGTTGG

PI594774 GTGAGTTGTGTCCTTGAAACTCTTGTTTTTTTTTTTCTTTCTTATTTTATGGTTCGTTGG

Blackhawk GTGAGTTGTGTCCTTGAAACTCTTGTTTTTTTTTT-CTTTCTTATTTTATGGTTCGTTGG

PI594891 TTGTGGCAAGGTCTGGTGTAGGATTTGTGCTTTGTTTACTTCCTTTTTTTT-GGTGGGGG

Williams82 TTGTGGCAAGGTCTGGTGTAGGATTTGTGCTTTGTTTACTTCCTTTTTTTTTGGTGGGGG

PI594774 TTGTGGCAAGGTCTGGTGTAGGATTTGTGCTTTGTTTACTTCCTTTTTTTTGGGGGGGGG

Blackhawk TTGTGGCAAGGTCTGGTGTAGGATTTGTGCTTTGTTTACTTCCTTTTTTTT-GGTGGGGG

PI594891 GTGAGCAAATGTTGGAAGCTCATTAATAAATTGGAAGTTATGATGAAAAGTTCAGGTTTT

Williams82 GTGAGCAAATGTTGGAAGCTCATTAATAAATTGGAAGTTATGATGAAAAGTTCAGGTTTT

PI594774 GTGAGCAAATGTTGGAAGCTCATTAATAAATTGGAAGTTATGATGAAAAGTTCAGGTTTT

Blackhawk GTGAGCAAATGTTGGAAGCTCATTAATAAATTGGAAGTTATGATGAAAAGTTCAGGTTTT

PI594891 TTAGGCCTTTTCTCTATGTTTAGTGGCTGAGGAATTGTTAGAGGTTGGTGTAAACTTTCA

Williams82 TTAGGCCTTTTCTCTATGTTTAGTGGCTGAGGAATTGTTAGAGGTTGGTGTAAACTTTCA

PI594774 TTAGGCCTTTTCTCTATGTTTAGTGGCTGAGGAATTGTTAGAGGTTGGTGTAAACTTTCA

Blackhawk TTAGGCCTTTTCTCTATGTTTAGTGGCTGAGGAATTGTTAGAGGTTGGTGTAAACTTTCA

PI594891 AGTTGCTGTTTTTTATATTTGTGTTACTTCATTGTCTTTGTCTTGCTTTTGATAAGCTGA

Williams82 AGTTGCTGTTTTTTATATTTGTGTTACTTCATTGTCTTTGTCTTGCTTTTGATAAGCTGA

PI594774 AGTTGCTGTTTTTTATATTTGTGTTACTTCATTGTCTTTGTCTTGCTTTTGATAAGCTGA

Blackhawk AGTTGCTGTTTTTTATATTTGTGTTACTTCATTGTCTTTGTCTTGCTTTTGATAAGCTGA

PI594891 AGTACTCTGCTAGGGTAAAAAGAGAGGATATTTTAAGAAATGAAAATTTCTGGTTTAGCT

Williams82 AGTACTCTGCTAGGGTAAAAAGAGAGGATATTTTAAGAAATGAAAATTTCCGGTTTAGCT

PI594774 AGTACTCTGCTAGGGTAAAAAGAGAGGATATTTTAAGAAATGAAAATTTCCGGTTTAGCT

Blackhawk AGTACTCTGCTAGGGTAAAAAGAGAGGATATTTTAAGAAATGAAAATTTCTGGTTTAGCT

PI594891 TCAAACGCTGATTTTGTAATTTATTTGGGTCTTTGATTTATTGTTTTTTTTTCTTTCAAT

Williams82 TCAAACGCTGATTTTGTAATTTATTTGGGTCTTTGATTTATG-GTTTTTTTTTTTTCAAT

PI594774 TCAAACGCTGATTTTGTAATTTATTTGGGTCTTTGATTTATG-GTTTTTTTTTTTTCAAT

Blackhawk TCAAACGCTGATTTTGTAATTTATTTGGGTCTTTGATTTATTGTTTTTTTTTCTTTCAAT

PI594891 ATTAGTCTGTTATTGACTCTGGTGTGTAGATGTTTGATTGTTTTGATAATGTGCTTTCCT

Williams82 ATTAGTCTGTTATTGACTCTGGTGTGTAGATGTTTGATTGTTTTGATAATGTGCATTCCT

PI594774 ATTAGTCTGTTATTGACTCTGGTGTGTAGATGTTTGATTGTTTTGATAATGTGCATTCCT

Blackhawk ATTAGTCTGTTATTGACTCTGGTGTGTAGATGTTTGATTGTTTTGATAATGTGCTTTCCT

PI594891 GAGCCAATCAACTGTAGAGGAATTTGCAGCTCATTCAGGGAATGTAAATTGTTTAAAACT

Williams82 GAGCCAATCAACTGTAGAGGAATTTGCAGCTCATTCAGGGAATGTAAATTGTTTAAAACT

PI594774 GAGCCAATCAACTGTAGAGGAATTTGCAGCTCATTCAGGGAATGTAAATTGTTTAAAACT

Blackhawk GAGCCAATCAACTGTAGAGGAATTTGCAGCTCATTCAGGGAATGTAAATTGTTTAAAACT

PI594891 TGGAAGGAAGGCGAACCGCCTTTTCATTACAGGAGGGGATGATCACAGTGTCAATCTATG

Williams82 TGGAAGGAAGGCGAACCGCCTTTTCATTACAGGAGGGGATGATCACAGTGTCAATCTATG

PI594774 TGGAAGGAAGGCGAACCGCCTTTTCATTACAGGAGGGGATGATCACAGTGTCAATCTATG

Blackhawk TGGAAGGAAGGCGAACCGCCTTTTCATTACAGGAGGGGATGATCACAGTGTCAATCTATG

PI594891 GATGATTGGGAAACCAACCTCTTTAATGGTCAGTTGATTATTGCCTTTGTGTTTTAACAT

Williams82 GATGATTGGGAAACCAACCTCTTTAATGGTCAGTTGATTATTGCCTTTGTGTTTTAACAT

PI594774 GATGATTGGGAAACCAACCTCTTTAATGGTCAGTTGATTATTGCCTTTGTGTTTTAACAT

Blackhawk GATGATTGGGAAACCAACCTCTTTAATGGTCAGTTGATTATTGCCTTTGTGTTTTAACAT

PI594891 TTTGGTTGCAATATGACTAAGGCAGGATTGTTAGGCAGTAATGAAGGTCCATTTCTTGTG

Williams82 TTTGGTTGCAATATGACTAAGGCAGGATTGTTAGGCAGTAATGAAGGTCCATTTCTTGTG

PI594774 TTTGGTTGCAATATGACTAAGGCAGGATTGTTAGGCAGTAATGAAGGTCCATTTCTTGTG

Blackhawk TTTGGTTGCAATATGACTAAGGCAGGATTGTTAGGCAGTAATGAAGGTCCATTTCTTGTG

PI594891 TGCC--GTGTTGCAGCTTGCAAGTAATGTTATTGTAATGGTAGTGACTGAAGGATTTATG

Williams82 TGCCCTGTGTTGCAGCTTGCAAGTAATGTTATTGTAATGGTAGTGACTGAAGGATTTATG

PI594774 TGCCCTGTGTTGCAGCTTGCAAGTAATGTTATTGTAATGGTAGTGACTGAAGGATTTATG

Blackhawk TGCC--GTGTTGCAGCTTGCAAGTAATGTTATTGTAATGGTAGTGACTGAAGGATTTATG

PI594891 CTATTGCTCGTGACTGAAGGACTTATTTTCAATTCATATTAAATGATAAGCTTAAATTTG

Williams82 CTATTGCTCGTGACTGAAGGACTTATTTTCAATTCATATTAAATGATAAGCTTAAATTTG

PI594774 CTATTGCTCGTGACTGAAGGACTTATTTTCAATTCATATTAAATGATAAGCTTAAATTTG

Blackhawk CTATTGCTCGTGACTGAAGGACTTATTTTCAATTCATATTAAATGATAAGCTTAAATTTG

PI594891 GACTGAAATAGGCATGGTATAACGATGATGGAGTATGATAGGAAGCAAGATTTTTACTCA

Williams82 GACTGAAATAGGCATGGTATAACGATGATGGAGTATGATAGGAAGCAAGATTTTTACTCA

PI594774 GACTGAAATAGGCATGGTATAA-GATGATGGAGTATGATAGGAAGCAAGATTTTTACTCA

Blackhawk GACTGAAATAGGCATGGTATAACGATGATGGAGTATGATAGGAAGCAAGATTTTTACTCA

PI594891 AGTATTACAACTAAAGATATTATTTACCAAAATCAGGATCCTTTAAATCTATTAACCAAT

Williams82 AGTATTACAACTAAAGAAATTATTTACCAAAATCAGGATCCTTTAAATCTATTAACCAAT

PI594774 AGTATTACAACTAAAGAAATTATTTACCAAAATCAGGATCCTTTAAATCTATTAACCAAT

Blackhawk AGTATTACAACTAAAGATATTATTTACCAAAATCAGGATCCTTTAAATCTATTAACCAAT

PI594891 ATGCATCACTTTTTGCCACATCATCTTGCACATATTTTGTGGTATTCAAAGATCGTGTGG

Williams82 ATGCATCACTTTTTGCCACATCATCTTGCACATATTTTGTAGTATTCAAAGATCGTGTGG

PI594774 ATGCATCACTTTTTGCCACATCATCTTGCACATATTTTGTGGTATTCAAAGATCGTGTGG

Blackhawk ATGCATCACTTTTTGCCACATCATCTTGCACATATTTTGTGGTATTCAAAGATCGTGTGG

PI594891 GCACGTGGGATTGTACTAACTACATCGACACAATCCAGTGAGATGGTGTCAGCATGGTTA

Williams82 GCACTTGGGATTGTACTAACTACATCGACACAATCCAGTGAGATGGTGTCAGCATGGTTA

PI594774 GCACGTGGGATTGTACTAACTACATCGACACAATCCAGTGAGATGGTGTCAGCATGGTTA

Blackhawk GCACGTGGGATTGTACTAACTACATCGACACAATCCAGTGAGATGGTGTCAGCATGGTTA

PI594891 ACACAACCAACACACCTAGGAAGAAAAGTAGCAGCAACCTACCAAGATGCCAAAATTGCT

Williams82 ACACAACCAACACACCTAGGAAGCAAAGTAGCAGCAACCTACCAAGATGCCAAAATTGCT

PI594774 ACACAACCAACACACCTAGGAAGCAAAGTAGCAGCAACCTACCAAGATGCCAAAATTGCT

Blackhawk ACACAACCAACACACCTAGGAAGAAAAGTAGCAGCAACCTACCAAGATGCCAAAATTGCT

PI594891 TTTTTACCAAAACAATTTTCCTTAATGACCAATTCAATGATCATTAATCAAACAAGTCTG

Williams82 TTTTTACCAAAACAATTTTCCTTAATGACCAATTCAATGATCATTAATCAAACAAGTCTG

PI594774 TTTTTACCAAAACAATTTTCCTTAATGACCAATTCAATGATCATTAATCAAACAAGTCTG

Blackhawk TTTTTACCAAAACAATTTTCCTTAATGACCAATTCAATGATCATTAATCAAACAAGTCTG

PI594891 ATGCCGACATTGTTTTTTTGCCACATAACTAGCTTGGAAGTTGCAAATGAAAATTTTGAA

Williams82 ATGCCAACATTGTTTTTTTGCCACATAACTAGCTTGGAAGTTGCAAATGAAAATTTTGAA

PI594774 ATGCCAACATTGTTTTTTTGCTACATAACTAGCTTGGAAGTTGCAAATGAAAATTTTGAA

Blackhawk ATGCCAACATTGTTTTTTTGCCACATAACTAGCTTGGAAGTTGCAAATGAAAATTTTGAA

PI594891 TCAAACCAAGATGAATGAACATTGAACTGTGTATGATCTCAAACATGCTAGCAATGAAAA

Williams82 TCAAACCAAGATGAATGAACATTGAACTGTGTATGATCTCAAACATGCTAGCAATGAAAA

PI594774 TCAAACCAAGATGAATGAACATTGAACTGTGTATGATCTCAAACATGCTAGCAATGAAAA

Blackhawk TCAAACCAAGATGAATGAACATTGAACTGTGTATGATCTCAAACATGCTAGCAATGAAAA

PI594891 GTGAAAAAATTCTTGAAACTTCCTACCATACATGGATCATAATAATCTATTATCTGTACC

Williams82 GTGAAAAAATTCTTGAAACTTCCTACCATACATGGATCATAATAATCTATTATCTGTACC

PI594774 GTGAAAAAATTCTTGAAACTTCCTACCATACATGGATCATAATAATCTATTATCTGTACC

Blackhawk GTGAAAAAATTCTTGAAACTTCCTACCATACATGGATCATAATAATCTATTATCTGTACC

PI594891 CTTTTTGCTGATTGGGTTCCGTTTGTGACATGGGTTTGACTTTCTTATGATAGCAGCAGA

Williams82 CTTTTTGCTGATTGGGTTCCCTTTGTGACATGGGTTTGACTTTCTTATGATAGCAGCAGA

PI594774 CTTTTTGCTGATTGGGTTCCCTTTGTGACATGGGTTTGACTTTCTTATGATAGCAGCAGA

Blackhawk CTTTTTGCTGATTGGGTTCCCTTTGTGACATGGGTTTGACTTTCTTATGATAGCAGCAGA

PI594891 GCCATGTTGAGCTTTAAACCTAACAGTGCTATCTTACTAAAAATGTTATGCATTATGCAG

Williams82 GCCATGTTGAGCTTTAAACCTAACAGTGCTATCTTACTAAAAATGTTATGCATTATGCAG

PI594774 GCCATGTTGAGCTTTAAACCTAACGGTGCTATCTTACTAAAAATGTTATGCATTATGCAG

Blackhawk GCCATGTTGAGCTTTAAACCTAACAGTGCTATCTTACTAAAAATGTTATGCATTATGCAG

PI594891 AGTTTGTGTGGTCACACTAGTTCAGTAGAATCAGTGACTTTTGACTCAGCAGAAGTGTTG

Williams82 AGTTTGTGTGGTCACACTAGTTCAGTAGAATCAGTGACTTTTGACTCAGCAGAAGTGTTG

PI594774 AGTTTGTGTGGTCACACTAGTTCAGTAGAATCAGTGACTTTTGACTCAGCAGAAGTGTTG

Blackhawk AGTTTGTGTGGTCACACTAGTTCAGTAGAATCAGTGACTTTTGACTCAGCAGAAGTGTTG

PI594891 ATTCTTTCTGGAGCATCGTCAGGGGTAATAAAGCTTTGGGATTTGGAAGAAGCAAAGAGT

Williams82 ATTCTTTCTGGAGCATCGTCAGGGGTAATAAAGCTTTGGGATTTGGAAGAAGCAAAGAGT

PI594774 ATTCTTTCTGGAGCATCGTCAGGGGTAATAAAGCTTTGGGATTTGGAAGAAGCAAAGAGT

Blackhawk ATTCTTTCTGGAGCATCGTCAGGGGTAATAAAGCTTTGGGATTTGGAAGAAGCAAAGAGT

PI594891 AAGTTAATTAGTATATTCTGAAGTCATGGACTCTATAGTATATAATGATGTTATTTATAT

Williams82 AAGTTAATTAGTATATTCTGAAGTCATGGACTCTATAATATATAATGATGTTATTTATAT

PI594774 AAGTTAATTAGTATATTCTGAAGTCATGGACTCTATAATATATAATGATGTTATTTATAT

Blackhawk AAGTTAATTAGTATATTCTGAAGTCATGGACTCTATAGTATATAATGATGTTATTTATAT

PI594891 GGCTTAAGACTTTTATGCTAGAGAAGAAGTGCAAACTTGCTGATCATAAACAGACAATTA

Williams82 GGCTTAAGACTTTTATGCTAGAGAAGAAGTGCAAACTTGCTGATCATAAACTGACAATTA

PI594774 GGCTTAAGACTTTTATGCTAGAGAAGAAGTGCAAACTTGCTGATCATAAACTGACAATTA

Blackhawk GGCTTAAGACTTTTATGCTAGAGAAGAAGTGCAAACTTGCTGATCATAAACAGACAATTA

PI594891 GTTGATTTAAGAATTGAGTTAAGCTATCTACTTTGCATGCATGATATTATAAGGGCATCT

Williams82 GTTGATTTAAGAATTGAGTTAAGCTATCTACTTTGCATGCATGAGATTATAAGGGCATCT

PI594774 GTTGATTTAAGAATTGAGTTAAGCTATCTACTTTGCATGCATGAGATTATAAGGGCATCT

Blackhawk GTTGATTTAAGAATTGAGTTAAGCTATCTACTTTGCATGCATGATATTATAAGGGCATCT

PI594891 TGGAGAACTATTATATATCCAAGTTAATCTTTTTGAATTGCCGGTGAAATGTAAGGTTCA

Williams82 TGGAGAACTATTATAAATCCAAGTTAATCTTTTTGAATTGCCGGTGAAATGTAAGGTTCA

PI594774 TGGAGAACTATTATAAATCCAAGTTAATCTTTTTGAATTGCCGGTGAAATGTAAGGTTCA

Blackhawk TGGAGAACTATTATATATCCAAGTTAATCTTTTTGAATTGCCGGTGAAATGTAAGGTTCA

PI594891 TGCATTTTCCAATACACCACTAATATGTGTGTAATTGTTCTTGAAGTATAGTGGTTCGCA

Williams82 TGCATTTTCCAATACACCACTAATATGTGTGTAATTGTTCTTGAAGTATAGTGGTTCGCA

PI594774 TGCATTTTCCAATACACCACTAATATGTGTGTAATTGTTCTTGAAGTATAGTGGTTCGCA

Blackhawk TGCATTTTCCAATACACCACTAATATGTGTGTAATTGTTCTTGAAGTATAGTGGTTCGCA

PI594891 CTCTTACCGGACACAGATTGAATTGCACTGCTGTTGAGTTTCATCCATTTGGTGAGTTTT

Williams82 CTCTTACCGGACACAGATTGAATTGCACTGCTGTTGAGTTTCATCCATTTGGTGAGTTTT

PI594774 CTCTTACCGGACACAGATTGAATTGCACTGCTGTTGAGTTTCATCCATTTGGTGAGTTTT

Blackhawk CTCTTACCGGACACAGATTGAATTGCACTGCTGTTGAGTTTCATCCATTTGGTGAGTTTT

PI594891 TTGCATCCGGCTCCTTGGATACTAATCTAAATATTTGGGATATCCGAAAAAAAGGATGCA

Williams82 TTGCATCCGGCTCCTTGGATACTAATCTAAATATTTGGGATATCCGAAAAAAAGGATGCA

PI594774 TTGCATCCGGCTCCTTGGATACTAATCTAAATATTTGGGATATCCGAAAAAAAGGATGCA

Blackhawk TTGCATCCGGCTCCTTGGATACTAATCTAAATATTTGGGATATCCGAAAAAAAGGATGCA

PI594891 TTCAAACATACAAGGGTCATAGCCAGGGCATCAGTACTATCAAATTCAGTCCTGATGGCC

Williams82 TTCAAACATACAAGGGTCATAGCCAGGGCATCAGTACTATCAAATTCAGTCCTGATGGCC

PI594774 TTCAAACATACAAGGGTCATAGCCAGGGCATCAGTACTATCAAATTCAGTCCTGATGGCC

Blackhawk TTCAAACATACAAGGGTCATAGCCAGGGCATCAGTACTATCAAATTCAGTCCTGATGGCC

PI594891 GTTGGGTTGTTTCTGGTGGATTTGATAATGTTGTGAAGGTAAATAGCATGGAGTTTGTTG

Williams82 GTTGGGTTGTTTCTGGTGGATTTGATAATGTTGTGAAGGTAAATAGCATGGAGTTTGTTG

PI594774 GTTGGGTTGTTTCTGGTGGATTTGATAATGTTGTGAAGGTAAATAGCATGGAGTTTGTTG

Blackhawk GTTGGGTTGTTTCTGGTGGATTTGATAATGTTGTGAAGGTAAATAGCATGGAGTTTGTTG

PI594891 TGGAATAGCTGAATAGCTATTAATTGTATATAACTTTTACAATTCTTCTGTGAATAAATT

Williams82 TGGAATAGCTGAATAGCTATTAATTGTATATAACTTTTACAATTCTTCTGTGAATAAATT

PI594774 TGGAATAGCTGAATAGCTATTAATTGTATATAACTTTTACAATTCTTCTGTGAATAAATT

Blackhawk TGGAATAGCTGAATAGCTATTAATTGTATATAACTTTTACAATTCTTCTGTGAATAAATT

PI594891 ATATGGATCTAACTTACCAATGTACAGGTCTGGGATCTAACAGGTGGAAAGCTCCTGCAT

Williams82 ATATGGATCTAACTTACCAATGTACAGGTCTGGGATCTAACAGGTGGAAAGCTCCTGCAT

PI594774 ATATGGATCTAACTTACCAATGTACAGGTCTGGGATCTAACAGGTGGAAAGCTCCTGCAT

Blackhawk ATATGGATCTAACTTACCAATGTACAGGTCTGGGATCTAACAGGTGGAAAGCTCCTGCAT

PI594891 GACTTCAAGTTCCATGAAGGACACATTAGATCCCTAGATTTTCATCCTCTTGAGTTTCTT

Williams82 GACTTCAAGTTCCATGAAGGACACATTAGATCCCTAGATTTTCATCCTCTTGAGTTTCTT

PI594774 GACTTCAAGTTCCATGAAGGACACATTAGATCCCTAGATTTTCATCCTCTTGAGTTTCTT

Blackhawk GACTTCAAGTTCCATGAAGGACACATTAGATCCCTAGATTTTCATCCTCTTGAGTTTCTT

PI594891 ATGGCTACAGGTGAGCTAGTGTACCTTAGAGGAGCTTGGAATGGTAGATGCTCATGCATG

Williams82 ATGGCTACAGGTGAGCTAGTGTACCTTAGAGGAGCTTGGAATGGTAGATGCTCATGCATG

PI594774 ATGGCTACAGGTGAGCTAGTGTACCTTAGAGGAGCTTGGAATGGTAGATCCTCATGCATG

Blackhawk ATGGCTACAGGTGAGCTAGTGTACCTTAGAGGAGCTTGGAATGGTAGATGCTCATGCATG

PI594891 TGCAATAAATTTGTAAAGATGCTTGTTTCATAATTATAATGTCATTTCAAGTGCATCAAA

Williams82 TGCAATAAATTTGTAAAGATCCTTGTTTCATAATTATAATGTCATTTCAAGTGCATCAAA

PI594774 TGCAATAAATTTGTAAAGATCCTTGTTTCATAATTATAATGTCATTTCAAGTGCATCAAA

Blackhawk TGCAATAAATTTGTAAAGATGCTTGTTTCATAATTATAATGTCATTTCAAGTGCATCAAA

PI594891 TGAGAATTTACTTAGGTTACATAGTTGGACAAGCTTAATGGAAATTTATGCAACAATGTT

Williams82 TGAGAATTTACTTAGGTTACATAGTTGGACAAGCTTAATGGAAATTTATGCAACAATGTT

PI594774 TGAGAATTTACTTAGGTTACATAGTTGGACAAGCTTAATGGAAATTTATGCAACAATGTT

Blackhawk TGAGAATTTACTTAGGTTACATAGTTGGACAAGCTTAATGGAAATTTATGCAACAATGTT

PI594891 CTATATAATATGCTACATCTGTCATCTGTGGAGGGACTAAGGAAATCTTTGAGCATCTTC

Williams82 CTATATAATATGCTACATCTTTCATCTGTGGAGGGACTAAGGAAATCTTTGAGCATCTTC

PI594774 CTATATAATATGCTACATCTTTCATCTGTGGAGGGACTAAGGAAATCTTTGAGCATCTTC

Blackhawk CTATATAATATGCTACATCTGTCATCTGTGGAGGGACTAAGGAAATCTTTGAGCATCTTC

PI594891 AAAAACTCA----GAGATTATCTAGAATGTATTCTGATCTAGCCCCCATAATGCTTTGTG

Williams82 AAAAACTCAATTAGAGATTATCTAGAATGTATTCTGGTCTAGCCCCCATAATGCTTTGTG

PI594774 AAAAACTCAATTAGAGATTATCTAGAATGTATTCTGGTCTAGCCCCCATAATGCTTTGTG

Blackhawk AAAAACTCA----GAGATTATCTAGAATGTATTCTGATCTAGCCCCCATAATGCTTTGTG

PI594891 GGTTGTATCAAATCATGTTTTGCTAACAATCCAAATTGCATGCATGCTCGGGTATAGACT

Williams82 GGTCGTATCAAATCATGTTTTGCTAACAATCCAAATTGCATGCATGCTCGGGTATAGACT

PI594774 GGTCGTATCAAATCATGTTTTGCTAACAATCCAAATTGCATGCATGCTCGGGTATAGACT

Blackhawk GGTTGTATCAAATCATGTTTTGCTAACAATCCAAATTGCATGCATGCTCGGGTATAGACT

PI594891 CTAGAGTGCATTTTTGGAGAGAAAAAAAGTTCTAAATATTTTACTGAAAATGCCATCTTA

Williams82 CTAGAGTGCATTTTTGGAGAGAAAAAAAGTTCTAAATATTTTACTGAAAATGCCATCTTA

PI594774 CTAGAGTGCATTTTTGGAGAGAAAAAAAGTTCTAAATATTTTACTGAAAATGCCATCTTA

Blackhawk CTAGAGTGCATTTTTGGAGAGAAAAAAAGTTCTAAATATTTTACTGAAAATGCCATCTTA

PI594891 GAAAAATTCATTTTTGGTGTAATTAACTAATATGTCTCTGTTGAAGTTTGTATATTTACT

Williams82 GAAAAATTCATTTTTGGTGTAATTAACTAATATGTCTCTGTTGAAGTTTGTATATTTACT

PI594774 GAAAAATTCATTTTTGGTGTAATTAACTAATATGTCTCTGTTGAAGTTTGTATATTTACT

Blackhawk GAAAAATTCATTTTTGGTGTAATTAACTAATATGTCTCTGTTGAAGTTTGTATATTTACT

PI594891 GATTTACACCAAACATAAAACATTGTTATTCAGCAAAAGAATAACAGTTCACAATTTGAT

Williams82 GATTTACACCAAACATAAAACATTGTTATTCAGCAAAACAATAACAGTTCACAATTTGAT

PI594774 GATTTACACCAAACATAAAACATTGTTATTCAGCAAAAGAATAACAGTTCACAATTTGAT

Blackhawk GATTTACACCAAACATAAAACATTGTTATTCAGCAAAAGAATAACAGTTCACAATTTGAT

PI594891 TTGCAGGTTCAGCAGATAGAACAGTGAAATTTTGGGATTTAGAAACCTTTGAACTGATTG

Williams82 ATGCAGGTTCAGCAGATAGAACAGTGAAATTTTGGGATTTAGAAACCTTTGAACTGATTG

PI594774 ATGCAGGTTCAGCAGATAGAACAGTGAAATTTTGGGATTTAGAAACCTTTGAACTGATTG

Blackhawk TTGCAGGTTCAGCAGATAGAACAGTGAAATTTTGGGATTTAGAAACCTTTGAACTGATTG

PI594891 GATCTACCAGACATGAGGTATCTAAACTTGCTGAAGCTTTCTTTTTCTGTTTGAAAGGAA

Williams82 GATCTACCAGACATGAGGTATCTAAACTTGCTGAAGCTTTCTTTTTCTGTTTGAAAGGAA

PI594774 GATCTACCAGACATGAGGTATCTAAACTTGCTGAAGCTTTCTTTTTCTGTTTGAAAGGAA

Blackhawk GATCTACCAGACATGAGGTATCTAAACTTGCTGAAGCTTTCTTTTTCTGTTTGAAAGGAA

PI594891 TCAATTATACCCATTAATATACCATAAATTCTGCTTCCAAATCTGCCTCTTATCAATTGA

Williams82 TCAATTATACCCATTAATATACCATAAATTCTGCTTCCAAATCTGCCTCTTATCAATTGA

PI594774 TCAATTATACCCATTAATATACCATAAATTCTGCTTCCAAATCTGCCTCTTATCAATTGA

Blackhawk TCAATTATACCCATTAATATACCATAAATTCTGCTTCCAAATCTGCCTCTTATCAATTGA

PI594891 ATTTTAGTTTTAATACATTTAGATCAGTTCTATTTACCACTACTATCTTTCTTGGAATCT

Williams82 ATTTTAGTTTTAATACATTTAGATCAGTTTTATTTACCACTACTATCTTTCTTGGAATCT

PI594774 ATTTTAGTTTTAATACATTTAGATCAGTTTTATTTACCACTACTATCTTTCTTGGAATCT

Blackhawk ATTTTAGTTTTAATACATTTAGATCAGTTCTATTTACCACTACTATCTTTCTTGGAATCT

PI594891 CTTTGGTAACCTTTTCAATGGATAACCCTGCTTGACAGTTGACACTTTTTATTTTACCAG

Williams82 CTTTGGTAACCTTTTCAATGGATAACCCTGCTTGACAGTTGACACTTTTTATTTTACCAG

PI594774 CTTTGGTAACCTTTTCAATGGATAACCCTGCTTGACAGTTGACACTTTTTATTTTACCAG

Blackhawk CTTTGGTAACCTTTTCAATGGATAACCCTGCTTGACAGTTGACACTTTTTATTTTACCAG

PI594891 TTTCCCTTTGCCATCTCATTGTTGTGGATAGTTTCCAAATGCAAAATGATTAGTTTAGTT

Williams82 TTTCCCTTTGCCATCACATTGTTGTGGATAGTTTCCAAATGCAAAATGATTAGTTTAGTT

PI594774 TTTCCCTTTGCCATCACATTGTTGTGGATAGTTTCCAAATGCAAAATGATTAGTTTAGTT

Blackhawk TTTCCCTTTGCCATCTCATTGTTGTGGATAGTTTCCAAATGCAAAATGATTAGTTTAGTT

PI594891 GTGACCTAGAACATTTCTGTAACTTTAAATTGCTAGTGTATCCTGCCCTGTTTTGTATCT

Williams82 GTGACCTAGAACATTTCTGTAACTTTAAATTGCTAGTGTATCCTGCCCTGTTTTGTATCT

PI594774 GTGACCTAGAACATTTCTGTAACTTTAAATTGCTAGTGTATCCTGCCCTGTTTTGTATCT

Blackhawk GTGACCTAGAACATTTCTGTAACTTTAAATTGCTAGTGTATCCTGCCCTGTTTTGTATCT

PI594891 TACAGATGCATAAAATGTTAATGTGTTTTTGTGTTTTAATTCATATACTATGATGTAGCT

Williams82 TACAGATGCATAAAATGTTGATGTGTTTTTGTGTTTTAAGTCATATACTATGATGTAGCT

PI594774 TACAGATGCATAAAATGTTAATGTGTTTTTGTGTTTTAAGTCATATACTATGATGTAGCT

Blackhawk TACAGATGCATAAAATGTTAATGTGTTTTTGTGTTTTAATTCATATACTATGATGTAGCT

PI594891 TAGTTGTCATTGTAGCATGGTAGGTATAGCATGTTGCTTTTTCTTCTAATGTAAGCCTGT

Williams82 TAGTCGTCATTGTAGCATGGTAGGTATAGCATGTTGCTTTTTCTTCTAATGTAAGCCTGT

PI594774 TAGTCGTCATTGTAGCATGGTAGGTATAGCATGTTGCTTTTTCTTCTAATTTAAGCCTGC

Blackhawk TAGTTGTCATTGTAGCATGGTAGGTATAGCATGTTGCTTTTTCTTCTAATGTAAGCCTGT

PI594891 AAATTTACATGAAATAGTAAACTCTAATAAGTATAAATTACTTTGTATTAGGTTTCAGGG

Williams82 AAATTTACATGAAATAGTAAACTCTAATAAGTATAAATTACTTTGTATTAGGTTTCGGGG

PI594774 AAATTTACATGAAATAGTAAACTCTAATAAGTATAAATTACTTTGTATTAGGTTTCGGGG

Blackhawk AAATTTACATGAAATAGTAAACTCTAATAAGTATAAATTACTTTGTATTAGGTTTCAGGG

PI594891 GTACGCTCAATTGCTTTTCATCCTGATGGACAGATCCTATTTGCTGGACTTGAGGATAGT

Williams82 GTACGCTCAATTGCTTTTCATCCTGATGGACAGATCCTATTTGCTGGATTTGAGGATAGT

PI594774 GTACGCTCAATTGCTTTTCATCCTGATGGACAGATCCTATTTGCTGGACTTGAGGATAGT

Blackhawk GTACGCTCAATTGCTTTTCATCCTGATGGACAGATCCTATTTGCTGGACTTGAGGATAGT

PI594891 TTGAAGGTACAGATTGTTTGTTTCAATAATTAGATATGATGAACAACTTAAACAGGATAA

Williams82 TTGAAGGTACAGATTGTTTGTTTCAATAATTAGATATGATGAACAACTTAAACAGGATAA

PI594774 TTGAAGGTACAGATTGTTTGTTTCAATAATTAGATATGATGAACAACTTAAACAGGATAA

Blackhawk TTGAAGGTACAGATTGTTTGTTTCAATAATTAGATATGATGAACAACTTAAACAGGATAA

PI594891 AGATATCATATCTAATGCTCATGTATTGCAGGTTTATTCATGGGAACCTGTTATATGTCA

Williams82 AGATATCATATCTAATGCTCATGTATTGCAGGTTTATTCGTGGGAACCTGTTATATGTCA

PI594774 AGATATCATATCTAATGCTCATGTATTGCAGGTTTATTCATGGGAACCTGTTATATGTCA

Blackhawk AGATATCATATCTAATGCTCATGTATTGCAGGTTTATTCATGGGAACCTGTTATATGTCA

PI594891 TGATGCTGTTGACATGGGATGGACAACACTTGGTGACCTATGTATTCATGACGGGATGCT

Williams82 TGATGCTGTTGACATGGGATGGACAACACTTGGTGACCTATGTATTCATGACGGGATGCT

PI594774 TGATGCTGTTGACATGGGATGGACAACACTTGGTGACCTATGTATTCATGACGGGATGCT

Blackhawk TGATGCTGTTGACATGGGATGGACAACACTTGGTGACCTATGTATTCATGACGGGATGCT

PI594891 TTTGGGTTGCTCGTTCTACAGTAATTCTGTGGGAGTATGGGTGTCAGATATTTCAGTAGG

Williams82 TTTGGGTTGCTCGTTCTACAGTAATTCTGTGGGAGTATGGGTGTCAGATATTTCAGTAGG

PI594774 TTTGGGTTGCTCGTTCTACAGTAATTCTGTGGGAGTATGGGTGTCAGATATTTCAGTAGG

Blackhawk TTTGGGTTGCTCGTTCTACAGTAATTCTGTGGGAGTATGGGTGTCAGATATTTCAGTAGG

PI594891 ACTCACTATCACTATATTAGTTAATATATTTATTTCTCAATTTATAACCTGATATCTGTG

Williams82 ACTCACTATCACTATATTAGTTAATATATTTATTTCTCAATTTATAACCTGATATCTGTG

PI594774 ACTCACTATCACTATATTAGTTAATATATTTATTTCTCAATTTATAACCTGATATCTGTG

Blackhawk ACTCACTATCACTATATTAGTTAATATATTTATTTCTCAATTTATAACCTGATATCTGTG

PI594891 TTAATTATATTTAGCTCAAAAGGTATGATTGTAGAATTACTACTTATGTGGTTGCTATTA

Williams82 TTAATTATATTTAGCTCAAAAGGTATGATTGTAGAATTACTACTTATGTGGTTGCTATTA

PI594774 TTAATTATATTTAGCTCAAAAGGTATGATTGTAGAATTACTACTTATGTGGTTGCTATTA

Blackhawk TTAATTATATTTAGCTCAAAAGGTATGATTGTAGAATTACTACTTATGTGGTTGCTATTA

PI594891 TCTTAATTGCTAGTTTTAAATTCAAAGCCACCATTTCTGTGGTCCTCCTACATGTCAACA

Williams82 TCTTAATTGCTAGTTTTAAATTCAAAGCCACCATTTCTGTGGTCCTCCTACATGTCAACA

PI594774 TCTTAATTGCTAGTTTTAAATTCAAAGCCACCATTTCTGTGGTCCTCCTACATGTCAACA

Blackhawk TCTTAATTGCTAGTTTTAAATTCAAAGCCACCATTTCTGTGGTCCTCCTACATGTCAACA

PI594891 ATGAATGTCATGACTACATGGCTTCCATATTAGTCTGTGAACAACAATAACTAATTTTGA

Williams82 ATGAATGTCATGACTACATGGCTTCCATATTAGTCTGTGAACAACAATAACTAATTTTGA

PI594774 ATGAATGTCATGACTACATGGCTTCCATATTAGTCTGTGAACAACAATAATTAATTTTGA

Blackhawk ATGAATGTCATGACTACATGGCTTCCATATTAGTCTGTGAACAACAATAACTAATTTTGA

PI594891 GATGCTTTTCCTGTAAATATGTTCTTTGTTAGCTAGCTTATCATTCTGATTGTTATTTTT

Williams82 GATGCTTTTCCTGTAAATAAGTTCTTTGTTAGCTAGCTTATCATTCTGATTGTTATTTTT

PI594774 GATGCTTTTCCTGTAAATAAGTTCTTTGTTAGCTAGCTTATCATTCTGATTGTTATTTTT

Blackhawk GATGCTTTTCCTGTAAATATGTTCTTTGTTAGCTAGCTTATCATTCTGATTGTTATTTTT

PI594891 AATGCATCAGCTTATAGAGCCATATAATGGTGGCTTGGAAACTGAGAAAAAGGAAAGCAC

Williams82 AATGCATCAGCTTATAGAGCCATATAATGGTGGCTTGGAAACTGAGAAAAAGGAAAGCAC

PI594774 AATGCATCAGCTTATAGAGCCATATAATGGTGGCTTGGAAACTGAGAAAAAGGAAAGCAC

Blackhawk AATGCATCAGCTTATAGAGCCATATAATGGTGGCTTGGAAACTGAGAAAAAGGAAAGCAC

PI594891 AAAACAGAAACTTAGTCTCCAGGGAAGACAAATGGAGAAAGTAGAAGCTGGTGTGGGACC

Williams82 AAAGCAGAAACTTAGTCTCCAGGGAAGACAAATGGAGAAAGTAGAAGCTGGTGTGGGACC

PI594774 AAAGCAGAAACTTAGTCTCCAGGGAAGACAAATGGAGAAAGTAGAAGCTGGTGTGGGACC

Blackhawk AAAACAGAAACTTAGTCTCCAGGGAAGACAAATGGAGAAAGTAGAAGCTGGTGTGGGACC

PI594891 GGCTTTTGGGTTGTGCAGTATGTCTGCTGACAATGAGTCAAAAGAGATAAAGAATATATA

Williams82 GGCTTTTGGGTTGTGCAGTATGTCTGCTGACAATGAGTCAAAAGAGATAAAGAATATATA

PI594774 GGCTTTTGGGTTGTGCAGTATGTCTGCTGACAATGAGTCAAAAGAGATAAAGAATATATA

Blackhawk GGCTTTTGGGTTGTGCAGTATGTCTGCTGACAATGAGTCAAAAGAGATAAAGAATATATA

PI594891 TATAGACTGTAAGCTACGGACCTTTTTGTCTACTATTGATATTAGACACGGCTTTATTAA

Williams82 TATAGACTGTAAGCTACGGACCTTTTTGTCTACTATTGATATTAGACACGGCTTTATTAA

PI594774 TATAGACTGTAAGCTACGGACCTTTTTGTCTACTATTGATATTAGACACGGCTTTATTAA

Blackhawk TATAGACTGTAAGCTACGGACCTTTTTGTCTACTATTGATATTAGACACGGCTTTATTAA

PI594891 AATTCTGAACAAATTCAGCTTTCAAAATAAAAAAAATTCCATAAAGTACATCTGAGCTCA

Williams82 AATTCTGAACAAATTCAGCTTTCAAAATAAAAAAAATTCCATAAAGTACATCTGAGCTCA

PI594774 AATTCTGAACAAATTCAGCTTTCAAAATAAAAAAAATTCCATAAAGTACATCTGAGCTCA

Blackhawk AATTCTGAACAAATTCAGCTTTCAAAATAAAAAAAATTCCATAAAGTACATCTGAGCTCA

PI594891 TTAATAGGTAGGATGCTGCAATTGCATCAGTGGATTGTTTGGTGAAGAATCTTCAGACAA

Williams82 TTAATAGGTAGGATGCTACAATTGCATCAGTGGATTGTTTGGTGGAGAATCTTCAGACAA

PI594774 TTAATAGGTAGGATGCTGCAATTGCATCAGTGGATTGTTTGGTGGAGAATCTTCAGGCAA

Blackhawk TTAATAGGTAGGATGCTGCAATTGCATCAGTGGATTGTTTGGTGAAGAATCTTCAGACAA

PI594891 TTTAATTTAACTGAATCAATCTGGAAATTTAATTATACTAGCATATATGTACTGAAGTTC

Williams82 TTTAATTTAACTGAATCAATCTGGAAATTTAATTATACTAGCATATATGTACTGAAGTTC

PI594774 TTTAATTTAACTGAATCAATCTGGAAATTTAATTATACTAGCATATATGTACTGAAGTTC

Blackhawk TTTAATTTAACTGAATCAATCTGGAAATTTAATTATACTAGCATATATGTACTGAAGTTC

PI594891 TTGACACGATGAATGTAAATTAAGATTATGGATAAGGCCTCTCTAAAGTGAATAAGTAGG

Williams82 TTGACACGATGAATGTAAATTAAGATTATGGATAAGGCCTCTCTAAAGTGAATAAGTAGG

PI594774 TTGACACGATGAATGTAAATTAAGATTGTGGATAAGGCCTCTCTAAAGTGAATAAGTAGG

Blackhawk TTGACACGATGAATGTAAATTAAGATTATGGATAAGGCCTCTCTAAAGTGAATAAGTAGG

PI594891 GCTATAATGCTATGTCCACCATGGAATTTATTGATGTGCATCATAATCATAACCCACAAA

Williams82 GCTATAATGCTATATCCACCATGGAATTTATTGATGTGCATCATAATCTTAACCCACAAA

PI594774 GCTATAATGCTATATCCACCATGGAATTTATTGATGTGCTTCATAATCTTAACCCACAAA

Blackhawk GCTATAATGCTATGTCCACCATGGAATTTATTGATGTGCATCATAATCATAACCCACAAA

PI594891 AATCTAAAGACGGGGCATCCTTACCTACTCACTTTAGAGGAGCTTTTTGTAAGATTATAT

Williams82 AATCTAAAGACGGGGCATCCTTACCTACTCACTTTAGAGGAGCTTTTTGTAAGATTATAT

PI594774 AATCTAAAGACGGGGCATCCTTACCTACTCACTTTAGAGGAGCTTTTTGTAAGATTATAT

Blackhawk AATCTAAAGACGGGGCATCCTTACCTACTCACTTTAGAGGAGCTTTTTGTAAGATTATAT

PI594891 ACGTTTTCCATTGTCAATTTTTGTGTATTTCTTTAATACTGTCCCAATGATGTATTGAAG

Williams82 ACGTTTTCCATCGTCAATTTTTGTGTAATTCTTTAATACTGTCCCAATGATGTATTGAAG

PI594774 ACGTTTTCCATCGTCAATTTTTGTGTAATTCTTTAATACTGTCCCAATGATGTATTGAAG

Blackhawk ACGTTTTCCATTGTCAATTTTTGTGTATTTCTTTAATACTGTCCCAATGATGTATTGAAG

PI594891 AACTTTTTTATTTTTATTTTTGCATATAAAGCTTCTGGAGGGAATCCTGATACCTTAATA

Williams82 AACTTTTTT--TTTTATTTTTGCATATAAAGCTTCTGGAGGGAATCCTGATACCTTAATA

PI594774 AACTTTTT-ATTTTTATTTTTGCATATAAAGCTTCTGGAGGGAATCCTGATACCTTAATA

Blackhawk AACTTTTTTATTTTTATTTTTGCATATAAAGCTTCTGGAGGGAATCCTGATACCTTAATA

PI594891 AGATCCAGGTCTTATAATTCTCCAAAAGTAGATCTTCCTGAAGAATCCAAAGAAATGCTA

Williams82 AGATCCAGGTCTTATAATTCTCCAAAAGTAGATCTTCCTGAAGAATCCAAAGAAATGCTA

PI594774 AGATCCAGGTCTTATAATTCTCCAAAAGTAGATCTTCCTGAAGAATCCAAAGAAATGCTA

Blackhawk AGATCCAGGTCTTATAATTCTCCAAAAGTAGATCTTCCTGAAGAATCCAAAGAAATGCTA

PI594891 AACTGGAGTCCTGCAACAAGAGCTCATGCAAAACAAAATGAACAAACACTTAGAAAGTCT

Williams82 AACTGGAGTCCTGCAACAAGAGCTCATGCAAAACAAAATGAACAAACACTTAGAAAGTCT

PI594774 AACTGGAGTCCTGCAACAAGAGTTCATGCAAAACAAAATGAACAAACACTTAGAAAGTCT

Blackhawk AACTGGAGTCCTGCAACAAGAGCTCATGCAAAACAAAATGAACAAACACTTAGAAAGTCT

PI594891 TACATTATGCCAAACTTTGTACCTCGGGATATTGTCAATGGTAAGAACTCAGCAACCTT

Williams82 TACATTATGCCAAACTTTGTACCTCGGGATATTGTCAATGGTAAGAACTCAGCAACCTT

PI594774 TACATTATGCCAAACTTTGTACCTCGGGATATTGTCAATGGTAAGAACTCAGCAACCTT

Blackhawk TACATTATGCCAAACTTTGTACCTCGGGATATTGTCAATGGTAAGAACTCAGCAACCTT

PI594891 TTCAAAGACTAAACCTGGGATGTTGCTTAAGCCAGTTCATGTTCAGGGAGCATCCACTGA

Williams82 TTCAAAGACTAAACCTGGAATGTTGCTTAAGCCAGTTCATGTTCAGGGAGCATCCACTGA

PI594774 TTCAAAGACTAAACCTGGGATGTTGCTTAAGCCAGTTCATGTTCAGGGAGCATCCACTGA

Blackhawk TTCAAAGACTAAACCTGGGATGTTGCTTAAGCCAGTTCATGTTCAGGGAGCATCCACTGA

PI594891 CATACTTGATGTTGATGGGTTTTCATCAGATTTGGATTCCAGAACATTTTGTGATACTGG

Williams82 CATACTTGATGTTGATGGGTTTTCATCAGATTTGGATTCCAGAACATTTTGTGATACTGG

PI594774 CATACTTGATGTTGATGGGTTTTCATCAGATTTGGATTCCAGAACATTTTGTGATACTGG

Blackhawk CATACTTGATGTTGATGGGTTTTCATCAGATTTGGATTCCAGAACATTTTGTGATACTGG

PI594891 AAGCAAATCTGATAGTGCAAAAGATCCTAACTTCCAAATGAAGCTTGGATCTCAGAATGA

Williams82 AAGCAAATCTGATAGTCCAAAAGATCCTAACTTCCAAATGAAGCTTGGATCTCAGAATGA

PI594774 AAGCAAATCTGATAGTCCAAAAGATCCTAACTTCCAAATGAAGCTTGGATCTCAGAATGA

Blackhawk AAGCAAATCTGATAGTGCAAAAGATCCTAACTTCCAAATGAAGCTTGGATCTCAGAATGA

PI594891 AGTCAGAGAATCTATTGAGGATAAGCATCCTATCAAAAGTGTCACAGAGAAGTTTGAAAA

Williams82 AGTCAGAGAATCTATTGAGGATAAGCATCCTATCAAAAGTGTCACAGAGAAGTTTGAAAA

PI594774 AGTCAGAGAATCTATTGAGGATAAGCATCCTATCAAAAGTGTCACAGAGAAGTTTGAAAA

Blackhawk AGTCAGAGAATCTATTGAGGATAAGCATCCTATCAAAAGTGTCACAGAGAAGTTTGAAAA

PI594891 AACTTTAACACCAGACAGATTTTCTGACCAAGATAAATGTAAGTTTTTTTCTGCCAAATC

Williams82 AACTTTAACACCAGACAGATTTTCTGACCAAGATAAATGTAAGTTTTTTTCTGCCAAATC

PI594774 AACTTTAACACCAGACAGATTTTCTGACCAAGATAAATGTAAGTTTTTTTCTGCCAAATC

Blackhawk AACTTTAACACCAGACAGATTTTCTGACCAAGATAAATGTAAGTTTTTTTCTGCCAAATC

PI594891 CCTTTATAGGTCAAATGCAAGTCATTGCTGATTTCTGGAATTGGCTCCTCTAAAATGATT

Williams82 CCTTTATAGGTCAAATGCAAGTCATTGCTGATTTCTGGAATTGGCTCCTCTAAAATGATT

PI594774 CCTTTATAGGTCAAATGCAAGTCATTGCTGATTTCTGGAATTGGCTCCTCTAAAATGATT

Blackhawk CCTTTATAGGTCAAATGCAAGTCATTGCTGATTTCTGGAATTGGCTCCTCTAAAATGATT

PI594891 AGTTCACTTTCAAGAAAAGTGTCACATCTCAAGTTCTCAACAATACTTGCAATTTCCTTT

Williams82 AGTTCACTTTCGAGCAAAGTGTCACATCTCAAGTTCTCAACAATACTTGCAATTTCCTTT

PI594774 AGTTCACTTTCGAGCAAAGTGTCACATCTCAAGTTCTCAACAATACTTGCAATTTCCTTT

Blackhawk AGTTCACTTTCAAGAAAAGTGTCACATCTCAAGTTCTCAACAATACTTGCAATTTCCTTT

PI594891 CTCAGTATACATTTCCACTCGTACAAAAAATGAGCTAGCTGCCCCACCATTCTCTTCTCT

Williams82 CTTAGTATACTTTTCCACTCGTACAAAAAATGAGCTAGCTGCCCCACCATTCTCTTCTCT

PI594774 CTTAGTATACTTTTCCACTCGTACAAAAAATGAGCTAGCTGCCCCACCATTCTCTTCTCT

Blackhawk CTCAGTATACATTTCCACTCGTACAAAAAATGAGCTAGCTGCCCCACCATTCTCTTCTCT

PI594891 ATTCTTAGTTATAGTGTGGGCTAAATTAGTGGCTAACATCATTTCCCTGAACTATATTTG

Williams82 ATTCTTAGTTATAGTGTGGGCTAAATTAGTGGCTAACATCATTTCCCTGAACTATATTTG

PI594774 ATTCTTAGTTATAGTGTGGGCTAAATTAGTGGCTAACATCATTTCCCTGAACTATATTTG

Blackhawk ATTCTTAGTTATAGTGTGGGCTAAATTAGTGGCTAACATCATTTCCCTGAACTATATTTG

PI594891 GCTTCTTATTGGTTGAATAACTGCTTTTCACTTTGATCCCATACCTATAGACTTGAATAA

Williams82 GCTTCTTATTAGTTGAATAACTGCTTTTCACTTTGATCCCATACCTATAGACTTGAATAA

PI594774 GCTTCTTATTGGTTGAATAACTGCTTTTCACTTTGATCCCATACCTATAGACTTGAATAA

Blackhawk GCTTCTTATTGGTTGAATAACTGCTTTTCACTTTGATCCCATACCTATAGACTTGAATAA

PI594891 GCCAAGTTCTAATCACATTGTGTTTCTCATCCATTCATTATATCTAAAATATCATACATT

Williams82 GCCAAGTTCTAATCACATTGTGTTTCTCTTCCATTCATTATATCTAAAATATCATACATT

PI594774 GCCAAGTTCTAATCACATTGTGTTTCTCTTCCATTCATTATATCTAAAATATCATACATT

Blackhawk GCCAAGTTCTAATCACATTGTGTTTCTCATCCATTCATTATATCTAAAATATCATACATT

PI594891 TGGGTCTTGCTCGACAAATCTGTGGAGATTTATACTGAACTTCTATGTATGCTTCTTATT

Williams82 TGGGTCTTGCTTGACAAATCTGTGGAGATTTATACTGAACTTCTATGTATGCTTCTTATT

PI594774 TGGGTCTTGCTTGACAAATCTGTGGAGATTTATACTGAACTTCTATGTATGCTTCTTATT

Blackhawk TGGGTCTTGCTTGACAAATCTGTGGAGATTTATACTGAACTTCTATGTATGCTTCTTATT

PI594891 CTATCAGAAAAAAATATGCTTGTTATTTCTGACTTCAATCTCCTGAAAAATATTGAATAA

Williams82 CTATAAGAAAAAAATATGCTTGTTATTTCTGACTTCAATCTCCTGAAAAATATTGAATAA

PI594774 CTATAAGAAAAAAATATGCTTGTTATTTCTGACTTCAATCTCCTGAAAAATATTGAATAA

Blackhawk CTATCAGAAAAAAATATGCTTGTTATTTCTGACTTCAATCTCCTGAAAAATATTGAATAA

PI594891 ACTGTTGGCCAACACTCTAGAGTCTTGACAGATAAACTCTTATAAAGATGTTAATAAACT

Williams82 ACTGTTGGCCAACACTCTTGAGTCTTGACAGATAAACTCTTACAAAGATGTTAATAAACT

PI594774 ACTGTTGGCCAACACTCTTGAGTCTTGACAGATAAACTCTTACAAAGATGTTAATAAACT

Blackhawk ACTGTTGGCCAACACTCTAGAGTCTTGACAGATAAACTCTTATAAAGATGTTAATAAACT

PI594891 GTGATTCTGACTTGTAGTTTATGGATATTTTAGTTCTACTTGTGTAAAAAGTTGGCATGC

Williams82 GTGATTCTGACTTGTAGTTTATGGATATTTTAGTTCTACTTGTGTAAAAAGTTGGCATGC

PI594774 GTGATTCTGACTTGTAGTTTATGGATATTTTAGTTCTACTTGTGTAAAAAGTTGGCATGC

Blackhawk GTGATTCTGACTTGTAGTTTATGGATATTTTAGTTCTACTTGTGTAAAAAGTTGGCATGC

PI594891 CATGTGCTTTAGGTCATTAATATGGGAAAATAAAATATTATCATCCATGGTTAATATGTG

Williams82 CATGTGCTTTAGGTCATTAATATGGGAAAATAAAATATTATCATCCATGGTTAATATGTG

PI594774 CATGTGCTTTAGGTCATTAATATGGGAAAATAAAATATTATCATCCATGGTTAATATGTG

Blackhawk CATGTGCTTTAGGTCATTAATATGGGAAAATAAAATATTATCATCCATGGTTAATATGTG

PI594891 ATCATCTTTCAGGTAATCAATCCTCTCCATACAGCAAGGAGATGAGTCCAGTTAAATATG

Williams82 ATCATCTTTCAGGTAATCAATCCTCTCCATACAGCAAGGAGATGAGTCCAGTTAAATATG

PI594774 ATCATCTTTCAGGTAATCAATCCTCTCCATACAGCAAGGAGATGAGTCCAGTTAAATATG

Blackhawk ATCATCTTTCAGGTAATCAATCCTCTCCATACAGCAAGGAGATGAGTCCAGTTAAATATG

PI594891 TCAATGGAGGTTTGCCATAGCTCATCAATGTTGTTTTATTATGCATTAAGAAAGCCCCTG

Williams82 TCAATGGAGGTTTGCCATAGCTCATCAATGTTGTTTTATTATGCACTAAGAAAGCCCCTG

PI594774 TCAATGGAGGTTTGCCATAGCTCATCAATGTTGTTTTATTATGCACTAAGAAAGCCCCTG

Blackhawk TCAATGGAGGTTTGCCATAGCTCATCAATGTTGTTTTATTATGCATTAAGAAAGCCCCTG

PI594891 GAAACTGAATAGTTTTAAATCCTGCTTTTGGCAGTTGCTGTTTTACAAGGAAGGACCCGT

Williams82 GAAACTGAATAGTTTTAAATCCTGCTTTTGGCAGTTGCTGTTTTACAAGGAAGGACCCGT

PI594774 GAAACTGAATAGTTTTAAATCCTGCTTTTGGCAGTTGCTGTTTTACAAGGAAGGACCCGT

Blackhawk GAAACTGAATAGTTTTAAATCCTGCTTTTGGCAGTTGCTGTTTTACAAGGAAGGACCCGT

PI594891 TCTTTGGTTGAGAGGTTTGAAAGACGGGAAAGAACTCCCACCGATGAGAATCAAGCTAAT

Williams82 TCTTTGGTTGAGAGGTTTGAAAGAAGGGAAAGAACTCCCACCGATGAGAATCAAGCTAAT

PI594774 TCTTTGGTTGAGAGGTTTGAAAGAAGGGAAAGAACTCCCACCGATGAGAATCAAGCTAAT

Blackhawk TCTTTGGTTGAGAGGTTTGAAAGACGGGAAAGAACTCCCACCGATGAGAATCAAGCTAAT

PI594891 GCAACCCCCGCCACAATATTCGAAAATAAGGAAAAAATTCTCAAAGAAGATCAAACTAAT

Williams82 GCAACCCCCACCACAATATTCGAAAATAAGGAAAAAATTCTCAAAGAAGATCAAACTAAT

PI594774 GCAACCCCCACCACAATATTCGAAAATAAGGAAAAAATTCTCAAAGAAGATCAAACTAAT

Blackhawk GCAACCCCCGCCACAATATTCGAAAATAAGGAAAAAATTCTCAAAGAAGATCAAACTAAT

PI594891 GCATCCCCCATCACCAGCATAACATCTGAGAAGGGTGAAAGATCTCCTTTCGGCGATGAT

Williams82 GCATCCCCCATCACCAGCATAACATCTGAGAAGGGTGAAAGATTTCCTTTCGGCGATGAT

PI594774 GCATCCCCCATCACCAGCATAACATCTGAGAAGGGTGAAAGATTTCCTTTCGGCGATGAT

Blackhawk GCATCCCCCATCACCAGCATAACATCTGAGAAGGGTGAAAGATCTCCTTTCGGCGATGAT

PI594891 CAAAACAATATGCCCAATGTTCCCAACACAACCAGTGAAACTGACAAGTCTGCTAACTTC

Williams82 CAAAACAATATGCCCAATGTTCCCAACACAACCAGTGAAACTGACAAGTCTGCTAACTTC

PI594774 CAAAACAATATGCCCAATGTTCCCAACACAACCAGTGAAACTGACAAGTCTGCTAACTTC

Blackhawk CAAAACAATATGCCCAATGTTCCCAACACAACCAGTGAAACTGACAAGTCTGCTAACTTC

PI594891 CTGGTAAAAATCTAGCTTGTAACAAGATATAGTTTAGTCAAGCTGGATTTTTTTGGGTCA

Williams82 CTGGTAAAAATCTAGCTTGTAACAAGATATAGTTTAGTCAAGCTGGATTTTTTTGGGTCA

PI594774 CTGGTAAAAATCTAGCTTGTAACAAGATATAGTTTAGTCAAGCTGGATTTTTTTGGGTCA

Blackhawk CTGGTAAAAATCTAGCTTGTAACAAGATATAGTTTAGTCAAGCTGGATTTTTTTGGGTCA

PI594891 TCTTGCTATCACTTTTTACGATTTGTTGCATTGGTTTAGTTGAACTTACTCAACTATATC

Williams82 TCTTGCTATCACTTTTTACGATTTGTTGCATTGGTTTAGTTGAACTTACTCAACTATATC

PI594774 TCTTGCTATCACTTTTTACGATTTGTTGCATTGGTTTAGTTGAACTTACTCAACTATATC

Blackhawk TCTTGCTATCACTTTTTACGATTTGTTGCATTGGTTTAGTTGAACTTACTCAACTATATC

PI594891 TTGTTATATAATGTCAGAAAGTTGAGCCTCAAGTTCTTGGAAGCAATTCAAACTCTGCAA

Williams82 TTGTTATATAATGTCAGAAAGTTGAGCCTCAAGTTCTTGGAAGCGATTCAAACTCTGCAA

PI594774 TTGTTATATAATGTCAGAAAGCTGAGCCTCAAGTTCTTGGAAGCGATTCAAACTCTGCAA

Blackhawk TTGTTATATAATGTCAGAAAGTTGAGCCTCAAGTTCTTGGAAGCAATTCAAACTCTGCAA

PI594891 ATGATGGAGAGATAATTGAAGGGCTGATGCAAACTCATGATGTAACATTAAGTAATCTCC

Williams82 ATGATGGAGAGATAATTGAAGGGCTGATGCAAACTCATGATGTAACATTAAGTAATCTCC

PI594774 ATGATGGAAAGATAATTGAAGGGCTGATGCAAACTCATGATGTAACATTAAGTAATCTCC

Blackhawk ATGATGGAGAGATAATTGAAGGGCTGATGCAAACTCATGATGTAACATTAAGTAATCTCC

PI594891 GTTCACGTTTGACAAAATTACAGGTATTTTAGCTAGTCAGATATCAATATCATTTGCTTT

Williams82 GTTCACGTTTGACAAAATTACAGGTATTTTAGCTAGTCAGATATCAATATCATTTGCTTT

PI594774 GTTCACGTTTGACAAAATTACAGGTATTTTAGCTAGTCAGATATCAATATCATTTGCTTT

Blackhawk GTTCACGTTTGACAAAATTACAGGTATTTTAGCTAGTCAGATATCAATATCATTTGCTTT

PI594891 CAGACAAAGCCCATGTTTAGAGTTGTAATCCCAATATCACTAAGTATTAATTTTAGACAT

Williams82 TAGATGAAGCCCGTGTTTAGACTTGTAATCCCAGTATCACTAAGTATTAATTTTAGACAT

PI594774 TAGATGAAGCCCGTGTTTAGACTTGTAATCCCAGTATCACTAAGTATTAATTTTAGACAT

Blackhawk CAGACAAAGCCCATGTTTAGAGTTGTAATCCCAATATCACTAAGTATTAATTTTAGACAT

PI594891 AAAACTTATAACAGTGTTTGGCTATAACTTATATGCTGAGATGTAAGGGTGCATTTTTGT

Williams82 AAAACTTATAACAGTGTTTGGCTATAACTTATATGCTGAGATGTAAGGGTGCATTTTTGT

PI594774 AAAACTTATAACAGTGTTTGGCTATAACTTATATGCTGAGATGTAAGGGTGCATTTTTGT

Blackhawk AAAACTTATAACAGTGTTTGGCTATAACTTATATGCTGAGATGTAAGGGTGCATTTTTGT

PI594891 TTAAGTTTTCTTAACAGGGTTATAGAAAGAAAGGATCCAGCTCCCCTAAAGTGAGCAGTT

Williams82 TTAAGTTTTCTTAACAGGGTTATAGAAAGAAAGGATCCGGCTCCCCTAAAGTGAGCAATT

PI594774 TTAAGTTTTCTTAACAGGGTTATAGAAAGAAAGGATCCGGCTCCCCTAAAGTGAGCAATT

Blackhawk TTAAGTTTTCTTAACAGGGTTATAGAAAGAAAGGATCCAGCTCCCCTAAAGTGAGCAGTT

PI594891 CACTTTAGAGGGTAAAGTAATATCAGTCATTGACAAGAAAATTAACAGTCGATGTTACTT

Williams82 CACTTTAGAGGGTAAAGTAATATCAGTCATTGACAAGAAAATTAACAGACGATGTTACTT

PI594774 CACTTTAGAGGGTAAAGTAATATCAGTCATTGACAAGAAAATTAACAGACGATGTTACTT

Blackhawk CACTTTAGAGGGTAAAGTAATATCAGTCATTGACAAGAAAATTAACAGTCGATGTTACTT

PI594891 TATCTATACTCACATTAAAGAAGCCAAATCCAGAAAGAAAATGACAAAAGCTGGAACAAT

Williams82 TATCTATACTCACATTAAAGAAGCCAAATCCAGAAAGAAAATGACAAAAGCTGGAACAAT

PI594774 TATCTATACTCACATTAAAGAAGCCAAATCCAGAAAGAAAATGACAAAAGCTGGAACAAT

Blackhawk TATCTATACTCACATTAAAGAAGCCAAATCCAGAAAGAAAATGACAAAAGCTGGAACAAT

PI594891 TTCGTTCCCCAATTGGTTCTAAGTAAGGTGCATTTGCAGGTAGTGCAGCATTTTTGGGAG

Williams82 TTCGTTCCCCAATTGGTTCTAAGTAAGGTGCATTTGCAGGTAGTGCAGCATTTTTGGGAG

PI594774 TTCGTTCCCCAATTGGTTCTAAGTAAGGTGCATTTGCAGGTAGTGCAGCATTTTTGGGAG

Blackhawk TTCGTTCCCCAATTGGTTCTAAGTAAGGTGCATTTGCAGGTAGTGCAGCATTTTTGGGAG

PI594891 CGGAATGATGCTAAAGGTGCCATCAGTGCTTTGAGAAAGTTGCCTGATCAATCTGTGCGT

Williams82 CGGAATGATACTAAAGGTGCCATCAGTGCTTTGAGAAAGTTGCCTGATCAATCTGTGCG-

PI594774 CGGAATGATACTAAAGGTGCCATCAGTGCTTTGAGAAAGTTGCCTGATCAATCTGTGCG-

Blackhawk CGGAATGATGCTAAAGGTGCCATCAGTGCTTTGAGAAAGTTGCCTGATCAATCTGTGCGT

PI594891 ATTTTTTTTATATTAAGTGGTCATGTGTCACTTGTTCGGTCTTTGTTTGTTCTGTATTAA

Williams82 TATTTTTTTATATTAAGTGGTCATGTGTCACTTGTTCGGTCTTTGTTTGTTCTGTATTAA

PI594774 TATTTTTTTATATTAAGTGGTCATGTGTCACTTGTTCGGTCTTTGTTTGTTCTGTATTAA

Blackhawk ATTTTTTTTATATTAAGTGGTCATGTGTCACTTGTTCGGTCTTTGTTTGTTCTGTATTAA

PI594891 ATGCTTTAACTAGTTATTTATGGTTTTGTTCTTTAATATATATTCCTGAAGATAACTTCC

Williams82 ATGCTTTAACTAGTTATTTATGGTTTTGTTCTTTAATATATAGTCCTGAAGATAACTTCC

PI594774 ATGCTTTAACTAGTTATTTATGGTTTTGTTCTTTAATATATAGTCCTGAAGATAACTTCC

Blackhawk ATGCTTTAACTAGTTATTTATGGTTTTGTTCTTTAATATATATTCCTGAAGATAACTTCC

PI594891 TCTGGATGTAGGTTCAAGCTGATGTTATCAGTGTCCTTGTTGATAAGATGGAGATTCTCA

Williams82 TCTGAATGTAGGTTCAAGCTGATGTTATCAGTGTCCTTGTTGATAAGATGGAGATTATCA

PI594774 TCTGAATGTAGGTTCAAGCTGATGTTATCAGTGTCCTTGTTGATAAGATGGAGATTATCA

Blackhawk TCTGGATGTAGGTTCAAGCTGATGTTATCAGTGTCCTTGTTGATAAGATGGAGATTCTCA

PI594891 CCTTAGATTTATTTTCTTGTTTACTTCCTGTGCTCACAGGTTTGTTGGATAGCAACATAG

Williams82 CCTTAGATTTATTTTCTTGTTTACTTCCTGTGCTCACAGGTTTGTTGGATAGCAACATAG

PI594774 CCTTAGATTTATTTTCTTGTTTACTTCCTGTGCTCACAGGTTTGTTGGATAGCAACATAG

Blackhawk CCTTAGATTTATTTTCTTGTTTACTTCCTGTGCTCACAGGTTTGTTGGATAGCAACATAG

PI594891 AAAGGTAATCCTTTATTAACCTGGTGTTAGGAAAATTCATGCTGGAAGTTAAAGTATGTG

Williams82 AAAGGTAATCCTTTATTAACATGGTGTTAGGAAAATTCATGCTGGAAGTTAAAGTATGTG

PI594774 AAAGGTAATCCTTTATTAACATGGTGTTAGGAAAATTCATGCTGGAAGTTAAAGTATGTG

Blackhawk AAAGGTAATCCTTTATTAACCTGGTGTTAGGAAAATTCATGCTGGAAGTTAAAGTATGTG

PI594891 AGGCCTCATATTTGCTTTCATCTAGTGTGCAACTTATAGTTTGTAGGCCGTGACCTCACA

Williams82 AGGCCTCATATTTGCTTTCATCTAGTGTGCAACTTATAGTTTGTAGGCCGTGACCTCACA

PI594774 AGGCCTCATATTTGCTTTCATCTAGTGTGCAACTTATAGTTTGTAGGCCGTGACCTCACA

Blackhawk AGGCCTCATATTTGCTTTCATCTAGTGTGCAACTTAGAGTTTGTAGGCCGTGACCTCACA

PI594891 AAAGCAGTTTAAATTGTCATGCATGTTCCTACTGATGGTCTTGCATATCACTGGGGATTT

Williams82 GAAGCAGTTTAAATTGTCATGCATGTTCCTACTGATGGTCTTGCATATCACTGGGGATTT

PI594774 GAAGCAGTTTAAATTGTCATGCATGTTCCTACTGATGGTCTTGCATATCACTGGGGATTT

Blackhawk AAAGCAGTTTAAATTGTCATGCATGTTCCTACTGATGGTCTTGCATATCACTGGGGATTT

PI594891 GGTCTTTAATCGAACTCAATGACGAGTGTGATCCACATAATCAACCTCACCTTGTAGGAT

Williams82 GGTCTTTAATTGAACTCAATGACGAGTGTGATCCACATAATCAACCTCACCTTGTAGGAT

PI594774 GGTCTTTAATTGAACTCAATGACGAGTGTGATCCACATAATCAACCTCACCTTGTAGGAT

Blackhawk GGTCTTTAATCGAACTCAATGACGAGTGTGATCCACATAATCAACCTCACCTTGTAGGAT

PI594891 TTGTTGTTCCTACTGATGGTGGCCTTTGCAGAAAATCACAATGTGGTTTTGGTAGACATT

Williams82 TTGTTGTTCCTACTGATGGTGGCCTTTGCAGAAAATCACAATGTGGTTTCGGTAGACATT

PI594774 TTGTTGTTCCTACTGATGGTGGCCTTTGCAGAAAATCACAATGTGGTTTCGGTAGACATT

Blackhawk TTGTTGTTCCTACTGATGGTGGCCTTTGCAGAAAATCACAATGTGGTTTTGGTAGACATT

PI594891 AACTCTGTTCTTACTGTGCAGACATGTGAAGGTGTCACTGGATATGCTGCTGAAGCTTGT

Williams82 AACTCTGTTCTTACTGTGCAGACATGTGAAGGTGTCACTGGATATGCTGCTGAAGCTTGT

PI594774 AACTCTGTTCTTACTGTGCAGACATGTGAAGGTGTCACTGGATATGCTGCTGAAGCTTGT

Blackhawk AACTCTGTTCTTACTGTGCAGACATGTGAAGGTGTCACTGGATATGCTGCTGAAGCTTGT

PI594891 GGCAGTTTTTGGTCCAACAATACGTTCAACCATTTCAGCACCTCCCTCTGTTGGGATTGA

Williams82 GGCAGTTTTTGGTCCAACAATACGTTCAACCATTTCAGCACCTCCCTCTGTTGGGATTGA

PI594774 GGCAGTTTTTGGTCCAACAATACGTTCAACCATTTCAGCACCTCCCTCTGTTGGGATTGA

Blackhawk GGCAGTTTTTGGTCCAACAATACGTTCAACCATTTCAGCACCTCCCTCTGTTGGGATTGA

PI594891 TCTACATGCAGAGCAAAGGTATCCTCCATCTTCACCTAATGTGGCTTTCGCTATCTCATA

Williams82 TCTACATGCAGAGCAAAGGTATCCTCCATCTTCACCTAATGTGGCTTTCGCTATCTCATA

PI594774 TCTACATGCAGAGCAAAGGTATCCTCCATCTTCACCTAATGTGGCTTTCGCTATCTCATA

Blackhawk TCTACATGCAGAGCAAAGGTATCCTCCATCTTCACCTAATGTGGCTTTCGCTATCTCATA

PI594891 AAGTTTATATGTTAATGATCCTAATTTATACAAAATCTTGAGAACAGGCGAGAATGCTCC

Williams82 AAGTTTATATGTTAATGATCCTAATTTATACAAAATCTTGAGAACAGGCGAGAATGCTCC

PI594774 AAGTTTATATGTTAATGATCCTAATTTATACAAAATCTTGAGAACAGGCGAGAATGCTCC

Blackhawk AAGTTTATATGTTAATGATCCTAATTTATACAAAATCTTGAGAACAGGCGAGAATGCTCC

PI594891 AATCAGTGCTTTATGCAACTGCAAAAGATCCGAATGATTCTTCCAATATTAATAAGGTAA

Williams82 AATCAGTGCTTTATGCAACTGCAAAAGATCCGAATGATTCTTCCAATATTAATAAGGTAA

PI594774 AATCAGTGCTTTATGCAACTGCAAAAGATCCGAATGATTCTTCCAATATTAATAAGGTAA

Blackhawk AATCAGTGCTTTATGCAACTGCAAAAGATCCGAATGATTCTTCCAATATTAATAAGGTAA

PI594891 ACATTTCATCTTTAGTGTTGCCAACTCATGCATACTGAAGTCAATCTTGCAGACTGATGA

Williams82 ACATTTCATCTTTAGTGTTGCCAACTCATGCATACTGAAGTCAATCTTGCAGACTGATGA

PI594774 ACATTTCATCTTTAGTGTTGCCAACTCATGCATACTGAAGTCAATCTTGCAGACTGATGA

Blackhawk ACATTTCATCTTTAGTGTTGCCAACTCATGCATACTGAAGTCAATCTTGCAGACTGATGA

PI594891 TTTTAAAATAGAAAACAAAAATAGTCCTTGCCAGTTTCTATTTCTTTATTTTGAACTTGT

Williams82 TTTTAAAATAGAAAACAAAAATAGTCCTTGCCAGTTTCTATTTCTTTATTTTGAACTTGT

PI594774 TTTTAAAATAGAAAACAAAAATAGTCCTTGCCAGTTTCTATTTCTTTATTTTGAACTTGT

Blackhawk TTTTAAAATAGAAAACAAAAATAGTCCTTGCCAGTTTCTATTTCTTTATTTTGAACTTGT

PI594891 CCTTGTATGACAAGATTTGCATGCCTTTGAAAAATTAGAGAAGTAGCATTTGCAACTATA

Williams82 CCTTGTATGACAAGATTTGCATGCCTTTGAAAAATTAGAGAAGTAGCATTTGCAACTATA

PI594774 CCTTGTATGACAAGATTTGCATGCCTTTGAAAAATTAGAGAAGTAGCATTTGCAACTATA

Blackhawk CCTTGTATGACAAGATTTGCATGCCTTTGAAAAATTAGAGAAGTAGCATTTGCAACTATA

PI594891 TATTCCTATGATAAAAAAAAAGGTATGTTAAACAATTTGCATTGCGTTTTGGGCTT

Williams82 TATTCCTATGAT--AAAAAAAGGTATGTTAAACAATTTGCATTGCGTTTTGGGCTT

PI594774 TATTCCTATGAT--AAAAAAAGGTATGTTAAACAATTTGCATTGCGTTTTGGGCTT

Blackhawk TATTCCTATGAT-AAAAAAAAGGTATGTTAAACAATTTGCATTGCATTTTGGGCTT

PI594891 TTGGCAGATAATGCATGTTTCTGTTGAATTGCTTCTCATTTTAATAAAGCATTAATTCCT

Williams82 TTGGCAGATAATGCATGTTTCTGTTGAATTGCTTCTCATTTTAATAAAGCAGTAATTCCT

PI594774 TTGGCAGATAATGCATGTTTCTGTTGAATTGCTTCTCATTTTAATAAAGCATTAATTCCT

Blackhawk TTGGCAGATAATGCATGTTTCTGTTGAATTGCTTCTCATTTTAATAAAGCATTAATTCCT

PI594891 CTCTCAGATTTACTAAACAACTTAATGCTTTTGATTCTTAACCTGTTTCTTTAGATATCA

Williams82 CTCTCAGATTTACTAAACAACTTAATGCTGTCAATTCTTAACCTGTTTCTTTAAATATCA

PI594774 CTCTCAGATTTACTAAACAACTTAATGCTGTCAATTCTTAACCTGTTTCTTTAAATATCA

Blackhawk CTCTCAGATTTACTAAACAACTTAATGCTGTTGATTCTTAACCTGTTTCTTTAAATATCA

PI594891 CTCTGACATTTATTTACATCTACATACAATCAAGAGTCGC--G--GTTTCACTTTTCCCC

Williams82 CTCTGACATTCATTTACATCTACATACAATCAAGAGTCGCTCGCTGTTTCACTTCTCCCC

PI594774 CTCTGACATTCATTTACATCTACATACAATCAAGAGTCGCTCGCTGTTTCACTTCTCCCC

Blackhawk CTCTGACATTTATTTACATCTACATACAATCAAGAGTCGC--G--GTTTCACTTTTCCCC

PI594891 TGCTTTTTGTTGCAGGAGGGGTGGTATATTGGCCAAGTCTGCCCAAGAGCTGAATTTAGT

Williams82 TGCTTTTTGTTGCAGGAGGGGTGGTATATTGGCCAAGTCTGCCCAAGAGCTGAATTTAGT

PI594774 TGCTTTTTGTTGCAGGAGGGGTGGTATATTGGCCAAGTCTGCCCAAGAGCTGAATTTAGT

Blackhawk TGCTTTTTGTTGCAGGAGGGGTGGTATATTGGCCAAGTCTGCCCAAGAGCTGAATTTAGT

PI594891 TCTTCAACAACCCTAACTAAAGGTGTTGTTTTATGAACTTCATCCAACTCACGAATGAGT

Williams82 TCTTCAACAACCCTAACTAAAGGTGTTGTTTTATGAACTTCATCCAACTCACGAATGAGT

PI594774 TCTTCAACAACCCTAACTAAAGGTGTTGTTTTATGAACTTCATCCAACTCACGAATGAGT

Blackhawk TCTTCAACAACCCTAACTAAAGGTGTTGTTTTATGAACTTCATCCAACTCACGAATGAGT

PI594891 GACTTGGCTTAACATGCTGCTTAGCAGGATTGTGTACAAAACATGAAGCCTTCATGTTGG

Williams82 GACTTGGCTTAACATGCTGCTTAGCAGGATTGTGTACAAAACATGAAGCCTTCATGTTGG

PI594774 GACTTGGCTTAACATGCTGCTTAGCAGGATTGTGTACAAAACATGAAGCCTTCATGCTGG

Blackhawk GACTTGGCTTAACATGCTGCTTAGCAGGATTGTGTACAAAACATGAAGCCTTCATGTTGG

PI594891 TCTTTTTCATCCACAGCCAACAATTTCCTTTCGAAGATTTGCACTAGCGACGGTTGTCAT

Williams82 TCTTTTTCATCCACAGCCAACAATTTCCTTTCGAAGATTTGCACTAGCGACGGTTGTCAT

PI594774 TCTTTTTCATCCACAGCCAACAATTTCCTTTCGAAGATTTGCACTAGCGACGGTTGTCAT

Blackhawk TCTTTTTCATCCACAGCCAACAATTTCCTTTCGAAGATTTGCACTAGCGACGGTTGTCAT

PI594891 CAGCTTTGTTTAATGGCAGAAAATTTTAGCCAAGTAAGATATGTGTGAGATATCTGAATG

Williams82 CAGCTTTGTTTAATGGCAGAAAATTTTAGCCAAGTAAGATATGTGTGAGATATCTGAATG

PI594774 CAGCTTTGTTTAATGGCAGAAAATTTTAGCCAAGTAAGATATGTGTGAGATATCTGAATG

Blackhawk CAGCTTTGTTTAATGGCAGAAAATTTTAGCCAAGTAAGATATGTGTGAGATATCTGAATG

PI594891 AATTATTAGACCTTGTGCTTAATTGCTTGAAAAGTATCTCAGTCTTCAACTGAGTGAATG

Williams82 AATTATTAGACCTTGTGCTTAATTGCTTGAAAAGTATCTCAGTCTTCAACTGAGTGAATG

PI594774 AATTATTAGACCTTGTGCTTAATTGCTTGAAAAGTATCTCAGTCTTCAACTGAGTGAATG

Blackhawk AATTATTAGACCTTGTGCTTAATTGCTTGAAAAGTATCTCAGTCTTCAACTGAGTGAATG

PI594891 TATCAGTCTGCTGTATTTAGTAGTTGTGCAAAATTCCTTGTATGTGCAAATATCCAATGA

Williams82 TATCAGTCTGCTGTATTTAGTAGTTGTGCAAAATTCCTTGTATGTGCAAATATCCAATGA

PI594774 TATCAGTCTGCTGTATTTAGTAGTTGTGCAAAATTCCTTGTATGTGCAAATATCCAATGA

Blackhawk TATCAGTCTGCTGTATTTAGTAGTTGTGCAAAATTCCTTGTATGTGCAAATATCCAATGA

PI594891 AATGTATCTTTTTTCTGGGGTCAAAGTACTCATGCTTCTTTTGTTAAAATAATTGGAGTG

Williams82 AATGTATCTTTTTTCTGGGGTCAAAGTACTCATGCTTCTTTTGTTAAAATAATTGGAGTG

PI594774 AATGTATCTTTTTTCTGGGGTCAAAGTACTCATGCTTCTTTTGTTAAAATAATTGGAGTG

Blackhawk AATGTATCTTTTTTCTGGGGTCAAAGTACTCATGCTTCTTTTGTTAAAATAATTGGAGTG

PI594891 GTCAGAACTTACAAATATGATGTTCACTGTACATTTAGATCATAGAATGCTCAATTTGAA

Williams82 GTCAGAACTTACAAATATGATGTTCACTGTACATTTAGATCATAGAATGCTCAATTTGAA

PI594774 GTCAGAACTTACAAATATGATGTTCACTGTACATTTAGATCATAGAATGCTCAATTTGAA

Blackhawk GTCAGAACTTACAAATATGATGTTCACTGTACATTTAGATCATAGAATGCTCAATTTGAA

PI594891 TACTGGGCAGATTAAGATTGTCATTACCAATTACCAAAGCTGCTCTTGGATTCTATCAAA

Williams82 TACTGGGCAGATTAAGATTGTCATTACCAATTACCAAAGCTGCTCTTGGATTCTATCAAA

PI594774 TACTGGGCAGATTAAGATTGTCATTACCAATTACCAAAGCTGCTCTTGGATTCTATCAAA

Blackhawk TACTGGGCAGATTAAGATTGTCATTACCAATTACCAAAGCTGCTCTTGGATTCTATCAAA

PI594891 ATAAATTTGTATTAAGGAGTAAGGACGATGATACTTGTGTTGACTTTATCATTATATAAG

Williams82 GTAAATTTGTATTAAGGAGTAAGGACGATGATACTTGTGTTGACTTTATCATTATATAAG

PI594774 GTAAATTTGTATTAAGGAGTAAGGACGATGATACTTGTGTTGACTTTATCATTATATAAG

Blackhawk GTAAATTTGTATTAAGGAGTAAGGACGATGATACTTGTGTTGACTTTATCATTATATAAG

PI594891 AGTTCGGCGAATAGGTTTTTTT

Williams82 AGTTCGGCGAATAGGTTTTTTT

PI594774 AGTTCGGCGAATAGGTTTTTTT

Blackhawk AGTTCGGCGAATAGGTTTTTTT

1. Glyma13g25350 protein alignment

PI594891 MAKTGYKLQEFAAHSGNVNCLKLGRKANRLFITGGDDHSVNLWMIGKPTSLMSLCGHTSS

W82 MAKTGYKLQEFAAHSGNVNCLKLGRKANRLFITGGDDHSVNLWMIGKPTSLMSLCGHTSS

PI594774 MAKTGYKLQEFAAHSGNVNCLKLGRKANRLFITGGDDHSVNLWMIGKPTSLMSLCGHTSS

Blackhawk MAKTGYKLQEFAAHSGNVNCLKLGRKANRLFITGGDDHSVNLWMIGKPTSLMSLCGHTSS

PI594891 VESVTFDSAEVLILSGASSGVIKLWDLEEAKMVRTLTGHRLNCTAVEFHPFGEFFASGSL

W82 VESVTFDSAEVLILSGASSGVIKLWDLEEAKMVRTLTGHRLNCTAVEFHPFGEFFASGSL

PI594774 VESVTFDSAEVLILSGASSGVIKLWDLEEAKMVRTLTGHRLNCTAVEFHPFGEFFASGSL

Blackhawk VESVTFDSAEVLILSGASSGVIKLWDLEEAKMVRTLTGHRLNCTAVEFHPFGEFFASGSL

PI594891 DTNLNIWDIRKKGCIQTYKGHSQGISTIKFSPDGRWVVSGGFDNVVKVWDLTGGKLLHDF

W82 DTNLNIWDIRKKGCIQTYKGHSQGISTIKFSPDGRWVVSGGFDNVVKVWDLTGGKLLHDF

PI594774 DTNLNIWDIRKKGCIQTYKGHSQGISTIKFSPDGRWVVSGGFDNVVKVWDLTGGKLLHDF

Blackhawk DTNLNIWDIRKKGCIQTYKGHSQGISTIKFSPDGRWVVSGGFDNVVKVWDLTGGKLLHDF

PI594891 KFHEGHIRSLDFHPLEFLMATGSADRTVKFWDLETFELIGSTRHEVSGVRSIAFHPDGQI

W82 KFHEGHIRSLDFHPLEFLMATGSADRTVKFWDLETFELIGSTRHEVSGVRSIAFHPDGQI

PI594774 KFHEGHIRSLDFHPLEFLMATGSADRTVKFWDLETFELIGSTRHEVSGVRSIAFHPDGQI

Blackhawk KFHEGHIRSLDFHPLEFLMATGSADRTVKFWDLETFELIGSTRHEVSGVRSIAFHPDGQI

PI594891 LFAG**L**EDSLKVYSWEPVICHDAVDMGWTTLGDLCIHDGMLLGCSFYSNSVGVWVSDISLI

W82 LFAGFEDSLKVYSWEPVICHDAVDMGWTTLGDLCIHDGMLLGCSFYSNSVGVWVSDISLI

PI594774 LFAG**L**EDSLKVYSWEPVICHDAVDMGWTTLGDLCIHDGMLLGCSFYSNSVGVWVSDISLI

Blackhawk LFAG**L**EDSLKVYSWEPVICHDAVDMGWTTLGDLCIHDGMLLGCSFYSNSVGVWVSDISLI

PI594891 EPYNGGLETEKKESTKQKLSLQGRQMEKVEAGVGPAFGLCSMSADNESKEIKNIYIDSSG

W82 EPYNGGLETEKKESTKQKLSLQGRQMEKVEAGVGPAFGLCSMSADNESKEIKNIYIDSSG

PI594774 EPYNGGLETEKKESTKQKLSLQGRQMEKVEAGVGPAFGLCSMSADNESKEIKNIYIDSSG

Blackhawk EPYNGGLETEKKESTKQKLSLQGRQMEKVEAGVGPAFGLCSMSADNESKEIKNIYIDSSG

PI594891 GNPDTLIRSRSYNSPKVDLPEESKEMLNWSPATRAHAKQNEQTLRKSYIMPNFVPRDIVN

W82 GNPDTLIRSRSYNSPKVDLPEESKEMLNWSPATRAHAKQNEQTLRKSYIMPNFVPRDIVN

PI594774 GNPDTLIRSRSYNSPKVDLPEESKEMLNWSPATR**V**HAKQNEQTLRKSYIMPNFVPRDIVN

Blackhawk GNPDTLIRSRSYNSPKVDLPEESKEMLNWSPATRAHAKQNEQTLRKSYIMPNFVPRDIVN

PI594891 GKNSATFSKTKPGMLLKPVHVQGASTDILDVDGFSSDLDSRTFCDTGSKSDS**A**KDPNFQM

W82 GKNSATFSKTKPGMLLKPVHVQGASTDILDVDGFSSDLDSRTFCDTGSKSDSPKDPNFQM

PI594774 GKNSATFSKTKPGMLLKPVHVQGASTDILDVDGFSSDLDSRTFCDTGSKSDSPKDPNFQM

Blackhawk GKNSATFSKTKPGMLLKPVHVQGASTDILDVDGFSSDLDSRTFCDTGSKSDS**A**KDPNFQM

PI594891 KLGSQNEVRESIEDKHPIKSVTEKFEKTLTPDRFSDQDKCNQSSPYSKEMSPVKYVNGVA

W82 KLGSQNEVRESIEDKHPIKSVTEKFEKTLTPDRFSDQDKCNQSSPYSKEMSPVKYVNGVA

PI594774 KLGSQNEVRESIEDKHPIKSVTEKFEKTLTPDRFSDQDKCNQSSPYSKEMSPVKYVNGVA

Blackhawk KLGSQNEVRESIEDKHPIKSVTEKFEKTLTPDRFSDQDKCNQSSPYSKEMSPVKYVNGVA

PI594891 VLQGRTRSLVERFERRERTPTDENQANATP**A**TIFENKEKILKEDQTNASPITSITSEKGE

W82 VLQGRTRSLVERFERRERTPTDENQANATPTTIFENKEKILKEDQTNASPITSITSEKGE

PI594774 VLQGRTRSLVERFERRERTPTDENQANATPTTIFENKEKILKEDQTNASPITSITSEKGE

Blackhawk VLQGRTRSLVERFERRERTPTDENQANATP**A**TIFENKEKILKEDQTNASPITSITSEKGE

PI594891 RSPFGDDQNNMPNVPNTTSETDKSANFLKVEPQVLGSNSNSANDGEIIEGLMQTHDVTLS

W82 RFPFGDDQNNMPNVPNTTSETDKSANFLKVEPQVLGSDSNSANDGEIIEGLMQTHDVTLS

PI594774 RFPFGDDQNNMPNVPNTTSETDKSANFLKAEPQVLGSDSNSANDGKIIEGLMQTHDVTLS

Blackhawk RSPFGDDQNNMPNVPNTTSETDKSANFLKVEPQVLGSNSNSANDGEIIEGLMQTHDVTLS

PI594891 NLRSRLTKLQVVQHFWERND**A**KGAISALRKLPDQSVQADVISVLVDKMEI**L**TLDLFSCLL

W82 NLRSRLTKLQVVQHFWERNDTKGAISALRKLPDQSVQADVISVLVDKMEIITLDLFSCLL

PI594774 NLRSRLTKLQVVQHFWERNDTKGAISALRKLPDQSVQADVISVLVDKMEIITLDLFSCLL

Blackhawk NLRSRLTKLQVVQHFWERND**A**KGAISALRKLPDQSVQADVISVLVDKMEI**L**TLDLFSCLL

PI594891 PVLTGLLDSNIERHVKVSLDMLLKLVAVFGPTIRSTISAPPSVGIDLHAEQRRECSNQCF

W82 PVLTGLLDSNIERHVKVSLDMLLKLVAVFGPTIRSTISAPPSVGIDLHAEQRRECSNQCF

PI594774 PVLTGLLDSNIERHVKVSLDMLLKLVAVFGPTIRSTISAPPSVGIDLHAEQRRECSNQCF

Blackhawk PVLTGLLDSNIERHVKVSLDMLLKLVAVFGPTIRSTISAPPSVGIDLHAEQRRECSNQCF

PI594891 MQLQKIRMILPILIRRGGILAKSAQELNLVLQQP

W82 MQLQKIRMILPILIRRGGILAKSAQELNLVLQQP

PI594774 MQLQKIRMILPILIRRGGILAKSAQELNLVLQQP

Blackhawk MQLQKIRMILPILIRRGGILAKSAQELNLVLQQP
